# Multiple Sclerosis-Associated Changes in the Composition and Immune Functions of Spore-Forming Bacteria

**DOI:** 10.1128/mSystems.00083-18

**Published:** 2018-11-06

**Authors:** Egle Cekanaviciute, Anne-Katrin Pröbstel, Anna Thomann, Tessel F. Runia, Patrizia Casaccia, Ilana Katz Sand, Elizabeth Crabtree, Sneha Singh, John Morrissey, Patrick Barba, Refujia Gomez, Rob Knight, Sarkis Mazmanian, Jennifer Graves, Bruce A. C. Cree, Scott S. Zamvil, Sergio E. Baranzini

**Affiliations:** a UCSF Weill Institute for Neurosciences, Department of Neurology, University of California, San Francisco, California, USA; b Institute for Human Genetics, University of California, San Francisco, California, USA; c Graduate Program for Biomedical Informatics, University of California, San Francisco, California, USA; d Icahn School of Medicine at Mount Sinai, New York, New York, USA; e Advanced Science Research Center at The Graduate Center of City University New York, New York, New York, USA; f University of California San Diego, San Diego, California, USA; g California Institute of Technology, Pasadena, California, USA; University of Colorado Denver

**Keywords:** immune mechanisms, multiple sclerosis, spore-forming bacteria

## Abstract

Multiple sclerosis (MS) is an autoimmune disease of the central nervous system characterized by adaptive and innate immune system dysregulation. Recent work has revealed moderate alteration of gut microbial communities in subjects with MS and in experimental, induced models. However, a mechanistic understanding linking the observed changes in the microbiota and the presence of the disease is still missing. Chloroform-resistant, spore-forming bacteria, which primarily belong to the classes *Bacilli* and *Clostridia* in the phylum *Firmicutes*, have been shown to exhibit immunomodulatory properties *in vitro* and *in vivo*, but they have not yet been characterized in the context of human disease. This study addresses the community composition and immune function of this bacterial fraction in MS. We identify MS-associated spore-forming taxa (primarily in the class *Clostridia*) and show that their presence correlates with impaired differentiation of IL-10-secreting, regulatory T lymphocytes *in vitro*. Colonization of antibiotic-treated mice with spore-forming bacteria allowed us to identify some bacterial taxa favoring IL-10^+^ lymphocyte differentiation and others inducing differentiation of proinflammatory, IFN-γ^+^ T lymphocytes. However, when fed into antibiotic-treated mice, both MS and control-derived spore-forming bacteria were able to induce similar IL-10-expressing Treg immunoregulatory responses, thus ameliorating symptoms of experimental allergic encephalomyelitis (EAE). Our analysis also identified Akkermansia muciniphila as a key organism that may interact either directly or indirectly with spore-forming bacteria to exacerbate the inflammatory effects of MS-associated gut microbiota. Thus, changes in the spore-forming fraction may influence T lymphocyte-mediated inflammation in MS. This experimental approach of isolating a subset of microbiota based on its functional characteristics may be useful to investigate other microbial fractions at greater depth.

**IMPORTANCE** To address the impact of microbiome on disease development, it is essential to go beyond a descriptive study and evaluate the physiological importance of microbiome changes. Our study integrates computational analysis with *in vitro* and *in vivo* exploration of inflammatory properties of spore-forming microbial communities, revealing novel functional correlations. We specifically show that while small differences exist between the microbiomes of MS patients and healthy subjects, these differences are exacerbated in the chloroform-resistant fraction. We further demonstrate that, when purified from MS patients, this fraction is correlated with impaired immunomodulatory responses *in vitro*.

**Author Video**: An author video summary of this article is available.

## Abstract (precis)

To address the impact of microbiome on disease development, it is essential to go beyond a descriptive study and evaluate the physiological importance of microbiome changes. Our study integrates computational analysis with *in vitro* and *in vivo* exploration of inflammatory properties of spore-forming microbial communities, revealing novel functional correlations. We specifically show that while small differences exist between the microbiomes of MS patients and healthy subjects, these differences are exacerbated in the chloroform-resistant fraction. We further demonstrate that, when purified from MS patients, this fraction is correlated with impaired immunomodulatory responses *in vitro*.

## INTRODUCTION

The human gut microbiota is emerging as a major immune regulator in health and disease, particularly in relation to autoimmune disorders. Most human microbiota studies to date have been based on unbiased exploration of complete microbial communities. However, limited sequencing depth, combined with high community richness and natural sample heterogeneity, might hinder the discovery of physiologically relevant taxonomical differences. Thus, targeted studies of specific microbial populations with defined characteristics may serve as a complementary approach to investigate disease-associated changes in gut microbiome.

Spore-forming bacteria constitute a subset of Gram-positive bacteria that are resistant to 3% chloroform treatment (1, 2) as well as other harsh conditions and show lower variability between humans compared to the total bacterial fraction (3). Both human and mouse spore-forming bacteria have immunoregulatory functions (4, 5). Mouse spore-forming bacteria include segmented filamentous bacteria and *Clostridia* species, which have been shown to induce gut T helper lymphocyte responses (4, 6). More recently, human spore-forming bacteria from a healthy subject were also reported to induce Tregs *in vitro* and in gnotobiotic mice (5). However, whether the composition and functions of spore-forming bacteria are altered in immune-mediated diseases is unknown.

Multiple sclerosis (MS) is a chronic disease of the central nervous system, characterized by autoimmune destruction of myelin. MS pathogenesis is in part mediated by effector T lymphocytes, and counterbalanced by Tregs, which limit the autoimmune damage inflicted by the former population (7, 8) and potentially promote remyelination (9). Recent studies, including our own, associated MS with moderate changes in the relative amounts of gut microbiota that exacerbate T lymphocyte-mediated inflammation *in vitro* and *in vivo* by stimulating pro-inflammatory IFN-γ^+^ Th1 and inhibiting IL-10^+^ regulatory T lymphocytes (10, 11).

We hypothesized that these MS-associated changes in gut microbial communities may involve spore-forming bacteria, thus altering their overall immunoregulatory properties. To address this hypothesis, we isolated spore-forming bacteria from untreated patients with relapsing-remitting MS (RRMS) and matched controls to analyze their structural composition by 16S rRNA gene sequencing. Furthermore, we also analyzed their immunoregulatory functions both *in vitro* and in the experimental autoimmune encephalomyelitis (EAE) mouse model.

## RESULTS

### MS-associated differences in microbial community composition are more evident in the spore-forming fraction.

**FIG 1:**
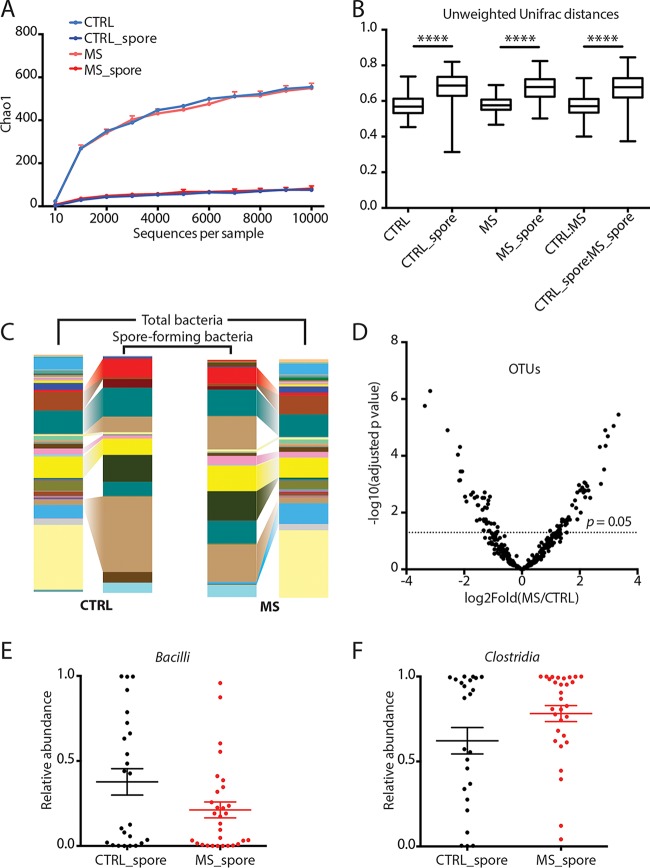
Differences in community composition of spore-forming bacterial fraction in MS patients and healthy controls. (A to C) Comparison of microbial community composition of spore-forming bacterial subset and total stool bacteria in untreated MS patients (*n* = 25) and controls (*n* = 24). (A) Chao1 metric of alpha diversity. (B) Median and range of distances (unweighted UniFrac distance matrix) within and between sample groups. (C) Mean relative abundance of microbial genera. (D to F) Comparison of relative abundances of individual microbial taxa in untreated MS patients (*n* = 25) and controls (*n* = 24). (D) Volcano plot of relative abundance distribution of microbial OTUs. *x* axis, log_2_ fold of relative abundance ratio between MS patients and controls after variance-stabilizing transformation. *y* axis, negative log_10_ of *P* value, negative binomial Wald test, Benjamini-Hochberg correction for multiple comparisons. (E and F) Relative abundances of bacterial classes *Bacilli* (E) and *Clostridia* (F) within phylum *Firmicut*es out of spore-forming bacteria from controls and MS patients. Error bars, mean ± SEM. CTRL, total stool bacteria from controls. CTRL_spore, spore-forming bacteria from controls. MS, total stool bacteria from MS patients. MS_spore, spore-forming bacteria from MS patients.

We isolated the spore-forming bacterial fraction from stool samples of 25 untreated MS patients and 24 controls and tested their relative abundance by amplicon sequencing of 16S rRNA V4 gene sequences. As expected, the observed overall complexity of each community was reduced (3) and no major differences in community richness between patients and controls were identified (Chao1 metric of alpha diversity, Fig. 1A) (Tables S2 and S3 in the supplemental material list the different OTUs detected after chloroform extraction in controls and cases, respectively). However, when bacterial abundances in the spore-forming fraction were analyzed at the OTU level, clear differences between cases and controls emerged (Fig. 1B). Specifically, 22.43% (135 out of 602 total) of OTUs were significantly different between cases and controls (*P* = 0.05, negative binomial Wald test, Benjamini-Hochberg correction) (Fig. 1D and Table S1). These taxonomical differences were noticeable even at the class level in which *Bacilli* were significantly overrepresented in controls (Fig. 1E), and *Clostridia* (including Clostridium perfringens) were significantly overrepresented in MS patients (Fig. 1F and Fig. S1).

10.1128/mSystems.00083-18.1 FIG S1 Relative abundance of Clostridium perfringens OTUs in spore-forming bacteria of MS patients and controls. *n* = 30 patients, 24 controls. *x* axis, OTU IDs taken from Greengenes 13.8 database. *y* axis, relative abundances after rarefaction to 10,000 reads. Download FIG S1, TIF file, 1.1 MB. Copyright © 2018 Cekanaviciute et al. 2018 Cekanaviciute et al. This content is distributed under the terms of the Creative Commons Attribution 4.0 International license.

10.1128/mSystems.00083-18.2 TABLE S1 Spore-forming OTUs that were significantly different between MS patients and controls. Negative binomial Wald test with Benjamini-Hochberg correction for multiple comparisons. Download Table S1, XLSX file, 0.01 MB. Copyright © 2018 Cekanaviciute et al. 2018 Cekanaviciute et al. This content is distributed under the terms of the Creative Commons Attribution 4.0 International license.

10.1128/mSystems.00083-18.3 TABLE S2 Different OTUs between spore-forming and total bacteria (controls). Negative binomial Wald test with Benjamini-Hochberg correction for multiple comparisons. Download Table S2, PDF file, 0.2 MB. Copyright © 2018 Cekanaviciute et al. 2018 Cekanaviciute et al. This content is distributed under the terms of the Creative Commons Attribution 4.0 International license.

10.1128/mSystems.00083-18.4 TABLE S3 Different OTUs between spore-forming and total bacteria (MS). Negative binomial Wald test with Benjamini-Hochberg correction for multiple comparisons. Download Table S3, PDF file, 0.2 MB. Copyright © 2018 Cekanaviciute et al. 2018 Cekanaviciute et al. This content is distributed under the terms of the Creative Commons Attribution 4.0 International license.

### Spore-forming bacteria from MS patients fail to induce anti-inflammatory T lymphocytes *in vitro*.

**FIG 2:**
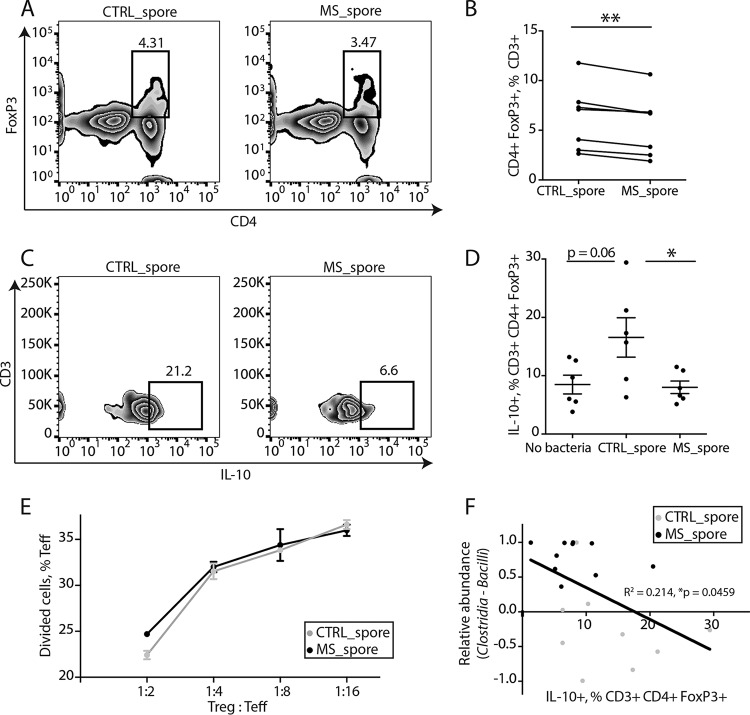
Spore-forming bacteria from MS patients inhibit IL-10^+^ Treg differentiation *in vitro*. (A and B) Representative flow cytometry plots (A) and quantification (B) of CD4^+^ FoxP3^+^ Tregs within CD3^+^ lymphocytes differentiated in response to spore-forming bacteria isolated from controls or untreated MS patients. *n* = 7 PBMC donors; each dot represents an average response from PBMC donor to isolates from 6 control or MS bacterial donors. **, *P* < 0.01, two-tailed repeated measures *t* test. (C and D) Representative flow cytometry plots (C) and quantification (D) of IL-10^+^ lymphocyte population within CD3^+^ CD4^+^ FoxP3^+^ Tregs differentiated in response to spore-forming bacteria isolated from controls or untreated MS patients. *n* = 6 bacterial donors per group. *, *P* < 0.05, two-tailed *t* test. Error bars, mean ± SEM. The experiment was repeated with nonoverlapping PBMC and bacterial donors and gave the same results. (E) Quantification of T effector cell proliferation in response to Tregs differentiated in the presence of spore-forming bacteria from MS patients or controls. *n* = 3 bacterial donors per group, each representing an average of 3 technical replicates. (F) Linear correlation between IL-10^+^ population within CD3^+^ CD4^+^ FoxP3^+^ Tregs and *Clostridia-Bacilli* relative abundances. *R*^2^ = 0.214, *P* = 0.0459. Black dots, MS patients. Light gray dots, controls.

To investigate whether MS-associated differences in community composition of spore-forming bacteria were sufficient to alter the immune functions of primary blood mononuclear cells (PBMCs) from healthy human donors, we exposed human PBMCs to extracts of spore-forming bacteria isolated either from unrelated controls or from MS patients and used flow cytometry to evaluate T lymphocyte differentiation under different polarizing conditions (12–14). A comparison of the PBMC response to extracts of spore-forming bacteria from controls or from MS patients identified lower conversion into CD4^+^ FoxP3^+^ Tregs (Fig. 2A and C), including the IL-10-expressing Treg population (Fig. 2B and D) in the PBMCs exposed to the MS-derived spore-forming bacteria. These data suggest that spore-forming bacteria from MS patients are significantly less effective at inducing Treg differentiation. Of note, the small population of Tregs that still differentiated in response to MS bacteria retained their suppressive capacities *in vitro* (Fig. 2E), thereby indicating that this was a functionally active population. Interestingly, the percentage of IL-10^+^ Tregs induced by extracts of spore-forming bacteria positively correlated with the relative abundance of *Bacilli* and negatively correlated with the relative abundance of *Clostridia* (Fig. 2F, expressed as *Clostridia-Bacilli* difference). Thus, the community composition of spore-forming bacteria (i.e., high *Clostridia*, low *Bacilli*) associated with MS was also correlated with an inhibition of their respective immunoregulatory functions.

### Antibiotic-treated and recolonized mouse models reveal associations between individual bacterial taxa and T lymphocyte responses.

**FIG 3:**
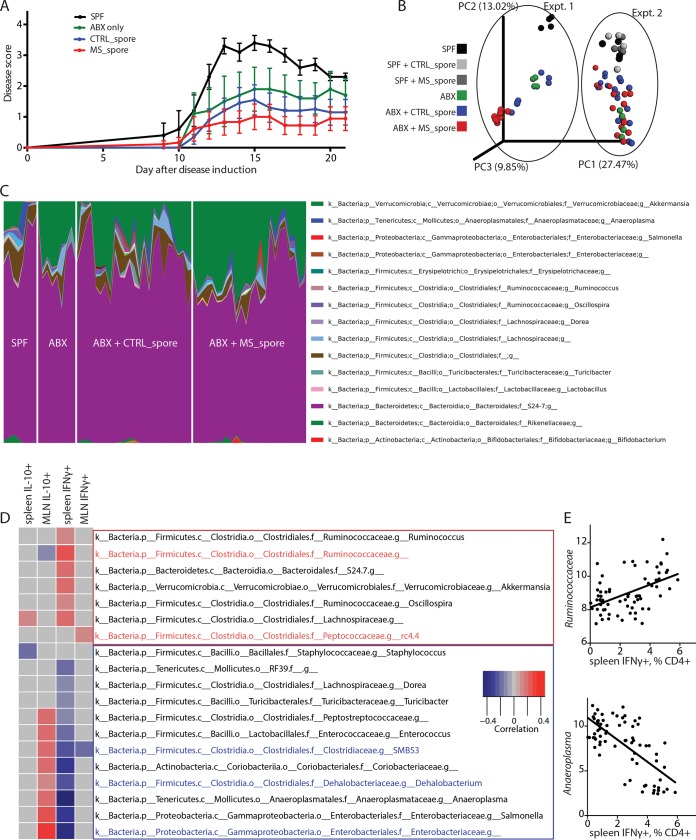
Spore-forming bacterial composition is correlated with T lymphocyte phenotypes *in vivo*. (A) Clinical EAE scores of mice that after antibiotic treatment had been colonized with spore-forming bacteria from controls (CTRL_spore) or MS patients (MS_spore) for 2 weeks or kept on antibiotics (ABX) or under SPF conditions as controls, prior to induction of EAE at 9 to 10 weeks of age. *n* = 5 to 10 mice per group. (B and C) Principal coordinate plot of beta diversity (PCoA; unweighted UniFrac) (B) and genus-level taxonomical distribution (C) of mouse fecal microbiota at 2 weeks of colonization with spore-forming bacteria, 2 separate experiments. (D) Bacterial genera whose abundance is correlated with changes in immune cell differentiation in antibiotic-treated and recolonized mice are shown. The linear correlation between relative abundances of bacterial genera and the percentage of IL-10^+^ regulatory and IFN-γ^+^ Th1 out of CD4^+^ Th lymphocytes from both spleens and mesenteric lymph nodes (MLN) of mice colonized with spore-forming bacteria is depicted as a heat map. Same samples as in panels B and C. Only the genera that show significant linear correlation with immune parameters (*P* > 0.05 after Benjamini-Hochberg adjustment for multiple comparisons) are included in the heat map. Red rectangle, putative proinflammatory subset. Blue rectangle, putative anti-inflammatory subset. Red font, taxa significantly increased in mice colonized with spore-forming bacteria from MS patients compared to controls. Blue font, taxa significantly reduced in mice colonized with spore-forming bacteria from MS patients compared to controls. (E) Examples of positive and negative correlation between bacteria and Th lymphocyte differentiation from panel D.

To determine whether the MS-associated reduction in the ability of spore-forming bacteria to stimulate Treg differentiation was physiologically significant, we colonized a group of female antibiotic-treated mice (15) with spore-forming bacteria from either controls (*n* = 2) or MS subjects (*n* = 2) and measured the course and severity of experimental allergic encephalomyelitis (EAE). We observed a significant reduction in disease severity in all mice whose GI tracts were reconstituted with spore-forming bacteria. However, this reduction was independent of whether the spore-forming fraction was isolated from MS or controls (Fig. 3A). This indicated that while MS-derived spore-forming bacteria could be functionally distinguished *in vitro*, these differences were not sufficient to induce a phenotype *in vivo* in our experimental setting.

We next analyzed whether spore-forming bacteria regulated T lymphocyte responses *in vivo*. To this end, we colonized antibiotic-treated mice with spore-forming bacteria from 3 controls and 3 MS patients and analyzed the resulting changes in bacterial composition and T lymphocyte differentiation. Principal coordinate analysis (PCoA) of the beta diversity of gut microbiota separated SPF mice from antibiotic-treated and recolonized mice. While no major shifts in community composition based on disease state of the donor were observed (Fig. 3B), multiple microbial taxa were differentially abundant (Fig. 3C; Tables S4 and S5), including an increase in *Akkermansia* (3 OTUs corresponding to *A. muciniphila*) (Table S5) in mice colonized with spore-forming bacteria from MS patients. Further investigation identified individual taxa that were classified as either putatively proinflammatory or anti-inflammatory based on the correlation between their relative abundance in mouse stool samples and their ability to alter differentiation of IFN-γ^+^ Th1 or IL-10^+^ regulatory lymphocytes from either spleen or mesenteric lymph nodes (MLN) *in vitro* (Fig. 3D and E). The putative proinflammatory category (Fig. 3D, red rectangle) included taxa significantly increased in mice colonized with spore-forming bacteria from MS patients compared to controls (highlighted in red), while the putative anti-inflammatory category (mostly evident in splenocytes; blue rectangle) contained taxa significantly reduced in mice colonized with spore-forming bacteria from MS patients (highlighted in blue).

10.1128/mSystems.00083-18.5 TABLE S4 Genera that were significantly different between antibiotic-treated mice colonized with spore-forming bacteria from MS patients and controls. Negative binomial Wald test with Benjamini-Hochberg correction for multiple comparisons. Download Table S4, PDF file, 0.1 MB. Copyright © 2018 Cekanaviciute et al. 2018 Cekanaviciute et al. This content is distributed under the terms of the Creative Commons Attribution 4.0 International license.

10.1128/mSystems.00083-18.6 TABLE S5 OTUs that were significantly different between antibiotic-treated mice colonized with spore-forming bacteria from MS patients and controls. Negative binomial Wald test with Benjamini-Hochberg correction for multiple comparisons. Download Table S5, PDF file, 0.1 MB. Copyright © 2018 Cekanaviciute et al. 2018 Cekanaviciute et al. This content is distributed under the terms of the Creative Commons Attribution 4.0 International license.

**FIG 4:**
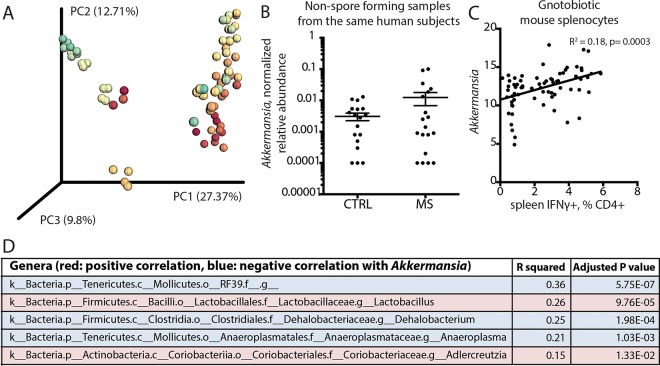
Increased *Akkermansia* is linked with MS-associated changes in spore-forming bacteria and proinflammatory T lymphocytes. (A) Principal coordinate plot of beta diversity (PCoA; unweighted UniFrac) of mouse fecal microbiota excluding *Akkermansia* at 2 weeks of colonization with spore-forming bacteria, 2 separate experiments, colored by *Akkermansia* presence (red to green: low to high). *P* < 0.001, significant contribution of *Akkermansia* presence to determining distance variation (Adonis method for continuous variables). (B) Relative abundance of *Akkermansia* in controls and MS patients used for isolation of spore-forming bacteria. *P* = 1.5E−09, negative binomial Wald test, Benjamini-Hochberg correction for multiple comparisons (across all 144 species detected in the data set). (C) Linear correlation of relative abundance of *Akkermansia* with IFN-γ^+^ Th1 lymphocyte differentiation in spleens of mice colonized with spore-forming bacteria. *R*^2^ = 0.18, *P* = 0.0003. (D) Bacterial genera significantly correlated with *Akkermansia in vivo*.

The increase in Akkermansia muciniphila, a non-spore-forming bacterium, in antibiotic-treated mice colonized with spore-forming bacteria from MS patients led to the hypothesis that spore-forming bacteria may regulate *Akkermansia* levels. The correlation between spore-forming community composition and relative abundance of *Akkermansia* is shown in Fig. 4A. The increase in *Akkermansia* was present not only in the mice colonized with spore-forming bacteria from MS donors but also in MS donors themselves (*P* = 1.5E−09, negative binomial Wald test) (Fig. 4B). Of interest, we and others (10, 11) recently reported the increased abundance of *Akkermansia* in untreated MS patients and identified this bacterium as sufficient for driving T lymphocyte differentiation into the proinflammatory IFN-γ^+^ Th1 phenotype *in vitro* (11). Consistent with this result, we also observed a significant positive correlation between the relative abundance of *Akkermansia* and IFN-γ^+^ Th1 lymphocyte differentiation (Fig. 4C) in antibiotic-treated and recolonized mice. While other taxa also correlated with *Akkermansia* levels and T lymphocyte differentiation (Fig. 4D), our data suggest that the observed immunological effects may be mediated by *Akkermansia* either directly, by shifting immune responses toward a Th1 phenotype (10), or indirectly, by affecting mucosal thickness and therefore stool transit time, potentially altering the growth of other communities with proinflammatory characteristics.

## DISCUSSION

The spore-forming fraction of gut bacteria has been associated with immunoregulatory properties (5). Here we examined the structural composition and immunological effects of the culturable spore-forming fraction of gut microbiota from subjects with MS compared to controls. MS-associated differences in bacterial community composition were correlated with impaired anti-inflammatory functions, as evidenced by a reduction in their ability to drive T lymphocyte differentiation into IL-10^+^ Tregs *in vitro*. Furthermore, colonizing antibiotic-treated mice with spore-forming bacteria allowed us to identify specific taxa correlated with T lymphocyte differentiation into IFN-γ^+^ and IL-10^+^ subtypes *in vivo*.

Our results contribute to the evidence supporting the immunoregulatory functions of spore-forming bacteria and show that these functions may be compromised in the context of autoimmunity. Some of the previous studies on spore-forming bacteria had been conducted by isolating this fraction from a single healthy donor (5, 16). This approach allowed focusing on donor-specific bacterial strains, but provided limited information about the “baseline” composition and variability of this bacterial community in healthy humans. Another recent study has compared multiple donors and discovered that spore-forming bacteria have reduced variability between subjects and respond to shared environmental signals, and in particular, dietary fatty acids, that likely mediate colonization of recently disturbed human guts (3). Here we used multiple healthy control donors to establish the baseline community composition of spore-forming bacteria, and compared these healthy profiles with those from patients with MS. MS is significantly more prevalent in women than in men; as a result there is always a gender disparity between cases and controls. However, at baseline there are few differences in microbiome between genders (17).

Our data corroborate previous findings that spore-forming bacteria, almost exclusively belonging to the phylum *Firmicutes*, and classes *Clostridia* and *Bacilli*, induce anti-inflammatory T lymphocytes *in vitro* and protect from autoimmune inflammation *in vivo* (5, 6). We also show that the taxonomical distribution and immunoregulatory functions of spore-forming bacteria are altered in MS patients. While we identified putative proinflammatory and anti-inflammatory taxa, their physiological functions remain to be determined, for example, by mouse monocolonization experiments as recently reported (18) While we were able to show that these differences have functional consequences *in vitro*, they were not sufficient to alter the course of EAE using antibiotic-treated mice. One possible explanation for this counterintuitive finding is that since our mice were treated with antibiotics, they were not germfree prior to colonization. As a consequence, unexpected interactions among antibiotic-resistant communities and the spore-forming fraction may have influenced the course of EAE. In addition, the fact that EAE immunization uses a microbial adjuvant (Mycobacterium tuberculosis) may have reduced the impact of microbiome on the immune response. We recognize that using GF mice for these experiments could address some of these concerns. However, raising GF animals is still a highly specialized enterprise available only at select institutions. Further studies of gene expression and metabolic output of spore-forming bacteria may provide therapeutic targets for regulating T lymphocyte responses to reduce autoimmune inflammation.

The mechanisms by which spore-forming bacteria regulate host T lymphocyte differentiation remain to be discovered. Interestingly, an overlapping subset of bacterial taxa has recently been shown to inhibit host proteases, including cathepsins (19), which mediate adaptive immune responses by increasing Th17 (20) and limiting Treg differentiation (21). Although future studies are needed to establish this firmly, it is possible that spore-forming bacteria from controls, but not MS patients, are able to stimulate Treg responses via cathepsin inhibition.

Furthermore, healthy human spore-forming bacteria both respond to fatty acid presence in the environment and produce short-chain fatty acids (SCFAs), including butyrate and acetate (22), which have been observed to stimulate Treg and inhibit Th1 differentiation *in vitro* and *in vivo* (23, 24). Either pure butyrate or butyrate-producing spore-forming bacteria from healthy humans have been shown to be sufficient for Treg induction (25) in mice. Thus, human T lymphocyte differentiation into Tregs may be driven by a yet-undiscovered SCFA-synthesizing subset of spore-forming bacteria that is present in controls and absent in MS patients.

Akkermansia muciniphila has previously been reported to be increased in MS patients compared to controls (10, 11, 26) and to have proinflammatory functions *in vitro* (11). The proinflammatory functions of *Akkermansia* may stem from its ability to induce thinning of intestinal mucosa. Indeed, MS patients present multiple gastrointestinal symptoms (27), which may be associated with differences in microbiome community composition, including the increase in *Akkermansia*. Mucosal disturbances have been previously reported to be sufficient to induce both microbial dysbiosis and immune impairments (28), which may account for an indirect proinflammatory effect of increased *Akkermansia*.

In addition, *Akkermansia* has been shown to be resistant to broad-spectrum antibiotics (29), which in part may explain its persistence in mice colonized with spore-forming bacteria. The fact that high levels of *Akkermansia* were seen only in mice colonized with MS chloroform-resistant bacteria suggests that its population is normally regulated by commensals that are depleted in MS, thus enabling *Akkermansia* overgrowth.

Our finding that Clostridium perfringens is more abundant in the spore-forming bacterial fraction of MS patients is consistent with the association of C. perfringens with neuromyelitis optica (NMO), another demyelinating autoimmune disease (30–32). Putative mechanisms of C. perfringens-mediated autoimmunity include molecular mimicry between C. perfringens peptide and a self-antigen in the human host (30) and toxin-mediated increase in neuronal damage (31, 33).

Due to the high variability of spore-forming bacteria across donors, mouse colonization with samples from additional donor pairs would be required to assess whether MS-associated reduction in regulatory T lymphocyte differentiation *in vitro* can be reliably reproduced *in vivo*. However, a major advantage of gnotobiotic as well as antibiotic-treated and recolonized mouse models is the ability to assess the association between immune responses and microbial abundance within experimental communities. The identification of additional taxa capable of inducing clear differentiation paths in immune cells will further contribute to our understanding of their role in immune regulation. For example, our findings corroborate the anti-inflammatory functions of relatively unknown bacterial genera such as *Anaeroplasma* and *Dehalobacterium* in mouse models of inflammation (34, 35).

In conclusion, we have investigated the immune functions of the spore-forming fraction of human gut microbiota in health and disease, using MS as a model of autoimmune inflammation. We identified novel bacterial taxa associated with MS as well as with T lymphocyte differentiation into both proinflammatory and regulatory phenotypes. Further studies of spore-forming bacteria and other experimentally defined bacterial populations may reveal specific immunoregulatory mechanisms in MS and other diseases that may be targeted by therapeutic interventions.

## MATERIALS AND METHODS

### Isolation of spore-forming bacteria from human fecal samples.

Fecal samples were collected from 25 adult patients with RRMS who had not received disease-modifying or steroid treatment for at least 3 months prior to the time of collection and 24 subjects without MS or any other autoimmune disorder (controls) at the University of California, San Francisco (UCSF) (Table 1). The inclusion criteria specified no use of antibiotics or oncologic therapeutics in 3 months prior to the study. All individuals signed a written informed consent in accordance with the sampling procedure approved by the local Institutional Review Board. Samples were stored in collection vials (Fisher no. NC9779954) at −80°C until bacterial isolation.

**TABLE 1:** Subject characteristics

Feature	Cases	Controls
*n*	25	24
Proportion female (%)	80.0	12.5
Mean age, yr (SD)	44.0 (±13.0)	49.3 (±12.0)
Average BMI (SD)	23.8 (±4.7)	24.2 (±4.2)
Average disease duration, yr (SD)	13.5 (±11.9)	N/A
Proportion off-therapy (%)	28	N/A
Proportion therapy naive (%)	72	N/A

Spore-forming bacteria were isolated based on their resistance to chloroform as described previously (5). Briefly, total bacteria were isolated from stool samples by suspending ∼0.5 mg stool sample in 1.5 ml PBS, passing it three times through a 70-μm cell strainer and washing twice with 1.5 ml PBS by spinning at 8,000 rpm. The resulting suspension was diluted in 5 ml PBS, mixed with chloroform to the final concentration of 3%, and incubated on a shaker for 1 h at room temperature. After incubation, chloroform was removed from the solution by bubbling nitrogen (N_2_) gas for 30 min. Chloroform-treated bacteria were then cultured on OxyPRAS brucella blood agar plates (Oxyrase no. P-BRU-BA) for 96 h followed by brucella broth (Anaerobe Systems no. AS-105) for 48 h and isolated for sequencing, *in vitro* experiments and *in vivo* experiments.

### 16S rRNA amplicon sequencing and computational analysis.

DNA was extracted from mouse fecal or human chloroform-resistant bacterial culture samples using the MoBio Power Fecal DNA extraction kit (MoBio no. 12830) according to the manufacturer’s instructions. For each sample, PCR targeting the V4 region of the prokaryotic 16S rRNA gene was completed in triplicate using the 515/806 primer pair, and amplicons were sequenced on NextSeq at the Microbiome Profiling Services core facility at UCSF using the sequencing primers and procedures described in the Earth Microbiome Project standard protocol (36). Analysis was performed using QIIME v1.9 as described (37). Essentially, amplicon sequences were quality-filtered and grouped to “species-level” OTUs via the SortMeRNA method (38), using the Greengenes v.13.8 97% data set for closed reference. Sequences that did not match reference sequences in the Greengenes database were dropped from analysis. Taxonomy was assigned to the retained OTUs based on the Greengenes reference sequence, and the Greengenes tree was used for all downstream phylogenetic community comparisons. OTUs were filtered to retain only OTUs present in at least 5% of samples and covering at least 100 total reads. After filtering, samples were rarefied to 10,000 sequences per sample. Alpha diversity was calculated using the Chao1 method (39). For analysis of beta diversity, pairwise distance matrices were generated using the phylogenetic metric unweighted UniFrac (40) and used for principal coordinate analysis (PCoA). For comparison of individual taxa, samples were not rarefied. Instead, OTU and taxa distributions were compared based on raw counts using the Wald negative binomial test from R software package DESeq2 as described previously (41, 42) with Benjamini-Hochberg correction for multiple comparisons. For visualization purposes, variance-stabilizing transformation was applied with local fit type. Linear correlations between bacterial taxa and lymphocyte proportions were computed after variance-stabilizing transformation of bacterial abundances (41). Human sample sequencing was performed in two batches, and they were used as a covariate for calculation.

### Mouse colonization with microbiota.

Female littermates, 5-week-old C57BL/6J mice (JAX no. 000664), cohoused at 5 mice per cage, were treated with a 1% solution of amphotericin B in drinking water for 3 days, followed by 2 weeks of a solution composed of 1% amphotericin B, 1 mg/ml ampicillin, 1 mg/ml neomycin, 1 mg/ml metronidazole and 0.5 mg/ml vancomycin in drinking water. Cages were changed weekly throughout the experiment using sterile technique. After 2 weeks, the drinking solution was replaced by sterile water and mice were gavaged with specific bacteria of interest at 2 × 10^8^ CFU in 100 μl per mouse every 2 days for 2 weeks (7 total gavages). Bacterial colonization was followed by either the induction of EAE or immunophenotyping of mesenteric and cervical lymph nodes.

To induce EAE, mice were immunized in both flanks with 0.1 ml MOG_35-55_ emulsion (1.5 mg/ml) mixed with complete Freund’s adjuvant (CFA) and killed Mycobacterium tuberculosis H37Ra (2 mg/ml), followed by two 0.1-ml intraperitoneal injections of pertussis toxin (2 µg/ml) immediately and at 48 h after MOG/CFA injections. Mice were scored daily in a blinded fashion for motor deficits as follows: 0, no deficit; 1, limp tail only; 2, limp tail and hind limb weakness; 3, complete hind limb paralysis; 4, complete hind limb paralysis and at least partial forelimb paralysis; 5, moribund. At the time of euthanasia, mouse mesenteric lymph nodes and spleens were dissected and processed by grinding tissues through a 70-µm cell strainer. Entire mesenteric and cervical lymph nodes and 10^7^ splenocytes per mouse were stimulated for 4 to 5 h with 20 ng/ml PMA and 1 µg/ml ionomycin in the presence of protein transport inhibitor (GolgiPlug, BD no. 51-2301KZ) and used immediately for immunophenotyping, while the remaining splenocytes were stored for *in vitro* bacterial stimulations. All animal research was approved by the institutional animal care and use committee (IACUC) at UCSF.

### Bacterial stimulation of human immune cells.

Human peripheral blood mononuclear cells were isolated from healthy volunteers and stored at −80°C in cryovials at a 10^7^-cell/ml concentration in FBS containing 10% DMSO. Before plating, cells were washed in PBS twice, recounted, and plated at a 10^6^-cell/ml concentration in RPMI medium supplemented with 10% FBS and 1% penicillin-streptomycin-glutamine. Cells were stimulated for 3 days as described previously (12) with anti-human CD3 (BD no.555336, 0.3 µg/ml), anti-human CD28 (BD no.555725, 2 µg/ml) and recombinant human TGF-β1 (R&D no. 240B002, 2.5 ng/ml).

Bacteria isolated from human chloroform-resistant cultures were resuspended in PBS supplemented with protease inhibitor (Roche no. 4693159001) and phosphatase inhibitor (Roche no. 4906845001), heat-inactivated at 65°C for 1 h and sonicated for 10 min as described previously (14). Protein concentration in the resulting suspension was measured using the Pierce BCA protein assay kit (Thermo Scientific no. 23227). Bacterial extracts were added to PBMCs at 1 µg/ml 1 h after plating as described previously (13). PBS with the same protease inhibitor and phosphatase inhibitor was added as the no-bacterium control. Each human *in vitro* experiment contained at least 6 independent donor bacterial samples and was repeated at least twice.

### Immunostaining, flow cytometry and FACS of human immune cells.

Human PBMCs were immunostained using standard protocols. Live/dead cell gating was achieved using the Live/Dead Fixable Aqua kit (ThermoFisher no. L34957). The FoxP3/transcription factor staining buffer set (eBioscience no. 00-5523-00) was used for staining of intracellular and intranuclear cytokines. The following antibodies were used for human PBMC staining: anti-CD3-PE.Cy7 (BD no. 563423), anti-CD4-PerCP.Cy5.5 (BioLegend no. 300530), anti-CD25-APC (BD no. 555434), anti-FoxP3-Alexa Fluor 488 (BD no. 560047) and anti-IL-10-PE (eBioscience no. 12-7108).

Flow cytometry was performed on a BD Fortessa cell analyzer and results were analyzed using FlowJo software (TreeStar). Cells were gated to identify the lymphocyte population based on forward and side scatter, followed by gating for single-color and live cell populations. Fluorescence minus one (FMO) was used for gating. Unstained, single-color and fluorescence-minus-one controls were used to identify stained populations. For T lymphocyte suppression assay, control CD4^+^ CD25^+^ lymphocytes were sorted from PBMC cultures incubated with extracts from unrelated control or MS spore-forming bacteria under Treg-differentiating conditions on an Aria III cell sorter (BD Biosciences) and cultured with CD4^+^ CD25^−^ cells from the same donor preloaded with a CFSE cell division tracker kit. Statistical significance of expression changes in markers of T lymphocyte differentiation and proliferation was determined using two-tailed Student’s *t* test to compare samples from different donors and two-tailed repeated measures *t* test to compare samples from the same donor. GraphPad Prism 6 software was used to analyze and plot the data. *P* < 0.05 was considered statistically significant.

### Data availability.

Raw and processed data are available at the UCSF datashare (DASH) platform (https://doi.org/10.7272/Q6FB5136).
