# Supplementary figures and images for: Multiple Sclerosis-Associated Changes in the Composition and Immune Functions of Spore-Forming Bacteria
Source: mSystems. 2018 Nov 6;3(6):e00083-18. doi: 10.1128/mSystems.00083-18 (PMC6222044; doi:10.1128/mSystems.00083-18)

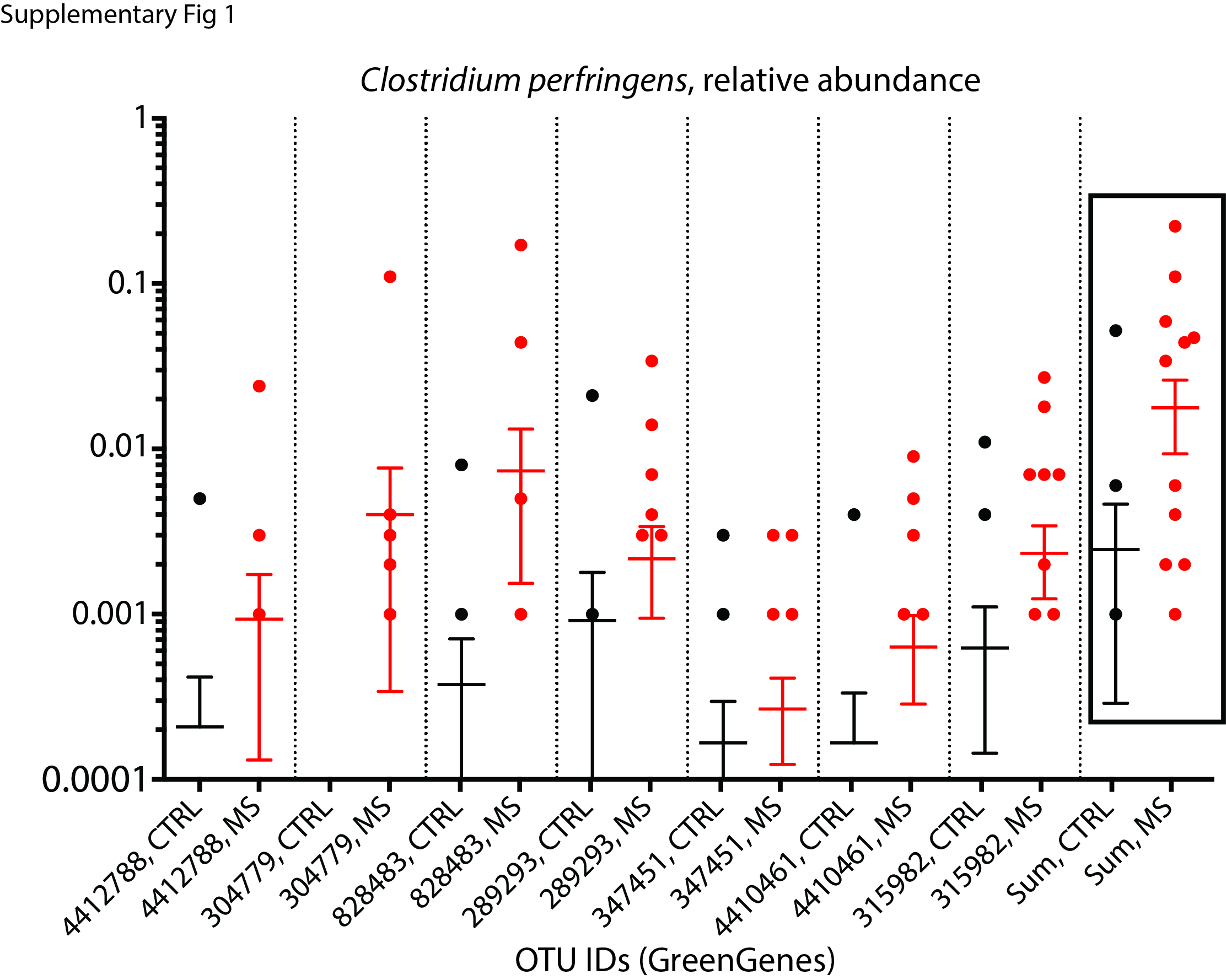

Supplement: FIG S1 [file sys006182286sf1.tif]
