# Supplementary material for: Multiple Sclerosis-Associated Changes in the Composition and Immune Functions of Spore-Forming Bacteria
Source: mSystems. 2018 Nov 6;3(6):e00083-18. doi: 10.1128/mSystems.00083-18 (PMC6222044; doi:10.1128/mSystems.00083-18)
Supplement: TABLE S2 [file sys006182286st2.pdf]

Supplementary Table 2. Different OTUs between spore-forming and total bacteria_CONTROLS

OTU (GreenGenes v.13.8)	taxonomy (blue: more in total bacteria, red: more in chloroform-resistant spore-forming fraction)	log2fold(Spore/Total)	adjusted p value
368490	k__Bacteria; p__Firmicutes; c__Bacilli; o__Turicibacterales; f__Turicibacteraceae; g__Turicibacter; s__	-13.47527926	7.95E-291
4468234	k__Bacteria; p__Bacteroidetes; c__Bacteroidia; o__Bacteroidales; f__Bacteroidaceae; g__Bacteroides; s__	8.774389403	4.53E-91
4481131	k__Bacteria; p__Firmicutes; c__Clostridia; o__Clostridiales; f__Ruminococcaceae; g__Faecalibacterium; s__prausnitzii	8.862100226	2.08E-70
4457438	k__Bacteria; p__Firmicutes; c__Clostridia; o__Clostridiales; f__Lachnospiraceae; g__; s__	8.383400752	7.13E-70
4478125	k__Bacteria; p__Firmicutes; c__Clostridia; o__Clostridiales; f__Ruminococcaceae; g__Faecalibacterium; s__prausnitzii	8.152202046	3.64E-63
4447072	k__Bacteria; p__Bacteroidetes; c__Bacteroidia; o__Bacteroidales; f__Bacteroidaceae; g__Bacteroides; s__	8.170295051	1.06E-62
4453609	k__Bacteria; p__Bacteroidetes; c__Bacteroidia; o__Bacteroidales; f__Rikenellaceae; g__; s__	7.676664088	4.86E-56
4467447	k__Bacteria; p__Bacteroidetes; c__Bacteroidia; o__Bacteroidales; f__Bacteroidaceae; g__Bacteroides; s__	7.78327333	9.26E-54
289734	k__Bacteria; p__Firmicutes; c__Clostridia; o__Clostridiales; f__Lachnospiraceae; g__; s__	-5.661963315	7.01E-53
347529	k__Bacteria; p__Firmicutes; c__Bacilli; o__Turicibacterales; f__Turicibacteraceae; g__Turicibacter; s__	-7.423346352	3.95E-52
4381430	k__Bacteria; p__Firmicutes; c__Clostridia; o__Clostridiales; f__Ruminococcaceae; g__Faecalibacterium; s__prausnitzii	7.482106591	1.17E-49
4416570	k__Bacteria; p__Firmicutes; c__Clostridia; o__Clostridiales; f__Lachnospiraceae; g__; s__	7.023944094	1.04E-47
4481427	k__Bacteria; p__Firmicutes; c__Clostridia; o__Clostridiales; f__Lachnospiraceae; g__Roseburia; s__	7.611020621	2.90E-45
4449054	k__Bacteria; p__Bacteroidetes; c__Bacteroidia; o__Bacteroidales; f__Bacteroidaceae; g__Bacteroides; s__	7.336941063	1.89E-43
4474380	k__Bacteria; p__Firmicutes; c__Clostridia; o__Clostridiales; f__Lachnospiraceae; g__Blautia; s__	6.346480056	4.40E-42
4457872	k__Bacteria; p__Bacteroidetes; c__Bacteroidia; o__Bacteroidales; f__Bacteroidaceae; g__Bacteroides; s__	6.826399488	2.63E-40
4426438	k__Bacteria; p__Firmicutes; c__Clostridia; o__Clostridiales; f__Ruminococcaceae; g__; s__	6.970926553	2.63E-40
191718	k__Bacteria; p__Firmicutes; c__Erysipelotrichi; o__Erysipelotrichales; f__Erysipelotrichaceae; g__; s__	6.919880101	2.23E-39
1952	k__Bacteria; p__Bacteroidetes; c__Bacteroidia; o__Bacteroidales; f__Porphyromonadaceae; g__Parabacteroides; s__	6.324170018	3.94E-38
4449236	k__Bacteria; p__Proteobacteria; c__Betaproteobacteria; o__Burkholderiales; f__Alcaligenaceae; g__Sutterella; s__	6.450573765	7.78E-38
185420	k__Bacteria; p__Bacteroidetes; c__Bacteroidia; o__Bacteroidales; f__Bacteroidaceae; g__Bacteroides; s__	6.606739527	1.36E-36
197072	k__Bacteria; p__Bacteroidetes; c__Bacteroidia; o__Bacteroidales; f__Bacteroidaceae; g__Bacteroides; s__uniformis	6.176648809	5.92E-36
4478815	k__Bacteria; p__Firmicutes; c__Clostridia; o__Clostridiales; f__Lachnospiraceae; g__Coprococcus; s__	6.253724694	6.90E-36
3576174	k__Bacteria; p__Firmicutes; c__Clostridia; o__Clostridiales; f__Clostridiaceae; g__; s__	-4.598270919	6.64E-34
4443172	k__Bacteria; p__Firmicutes; c__Clostridia; o__Clostridiales; f__Lachnospiraceae; g__; s__	6.852991293	1.46E-33
4365130	k__Bacteria; p__Bacteroidetes; c__Bacteroidia; o__Bacteroidales; f__Porphyromonadaceae; g__Parabacteroides; s__distasonis	6.154586804	9.46E-33
4414476	k__Bacteria; p__Firmicutes; c__Clostridia; o__Clostridiales; f__Ruminococcaceae; g__; s__	6.338693216	6.37E-32
4381553	k__Bacteria; p__Bacteroidetes; c__Bacteroidia; o__Bacteroidales; f__Bacteroidaceae; g__Bacteroides; s__	6.823534173	2.32E-31
4352657	k__Bacteria; p__Firmicutes; c__Clostridia; o__Clostridiales; f__Lachnospiraceae; g__Blautia; s__	5.924435213	1.36E-30
2582660	k__Bacteria; p__Firmicutes; c__Clostridia; o__Clostridiales; f__Lachnospiraceae; g__Blautia; s__	5.818787857	1.50E-30
4392188	k__Bacteria; p__Firmicutes; c__Clostridia; o__Clostridiales; f__Lachnospiraceae; g__Roseburia; s__faecis	5.949573054	6.86E-29
193233	k__Bacteria; p__Bacteroidetes; c__Bacteroidia; o__Bacteroidales; f__Bacteroidaceae; g__Bacteroides; s__	5.485888879	3.99E-28
176306	k__Bacteria; p__Firmicutes; c__Clostridia; o__Clostridiales; f__Lachnospiraceae; g__; s__	5.370798096	5.98E-27
4445673	k__Bacteria; p__Firmicutes; c__Clostridia; o__Clostridiales; f__Clostridiaceae; g__Clostridium; s__	-3.962703199	2.40E-26
4346675	k__Bacteria; p__Firmicutes; c__Clostridia; o__Clostridiales; f__Ruminococcaceae; g__; s__	6.23787943	4.39E-26
3472078	k__Bacteria; p__Bacteroidetes; c__Bacteroidia; o__Bacteroidales; f__Bacteroidaceae; g__Bacteroides; s__	5.30972222	1.39E-25
161423	k__Bacteria; p__Bacteroidetes; c__Bacteroidia; o__Bacteroidales; f__Bacteroidaceae; g__Bacteroides; s__	5.862003203	1.75E-25
4454586	k__Bacteria; p__Bacteroidetes; c__Bacteroidia; o__Bacteroidales; f__[Odoribacteraceae]; g__Odoribacter; s__	5.384914056	7.80E-25
198449	k__Bacteria; p__Bacteroidetes; c__Bacteroidia; o__Bacteroidales; f__Bacteroidaceae; g__Bacteroides; s__	5.045934636	1.05E-24
174818	k__Bacteria; p__Firmicutes; c__Clostridia; o__Clostridiales; f__Ruminococcaceae; g__; s__	5.051964564	1.30E-24
4477861	k__Bacteria; p__Bacteroidetes; c__Bacteroidia; o__Bacteroidales; f__Bacteroidaceae; g__Bacteroides; s__	6.336377245	2.61E-24
4471854	k__Bacteria; p__Firmicutes; c__Clostridia; o__Clostridiales; f__Lachnospiraceae; g__Coprococcus; s__	5.695952758	1.22E-23
173969	k__Bacteria; p__Firmicutes; c__Clostridia; o__Clostridiales; f__Lachnospiraceae; g__Coprococcus; s__	5.467275876	1.34E-22
4464173	k__Bacteria; p__Firmicutes; c__Clostridia; o__Clostridiales; f__Lachnospiraceae; g__; s__	5.41501533	5.61E-22
4447188	k__Bacteria; p__Bacteroidetes; c__Bacteroidia; o__Bacteroidales; f__Bacteroidaceae; g__Bacteroides; s__uniformis	6.205688762	6.67E-22

4424063	k__Bacteria; p__Firmicutes; c__Clostridia; o__Clostridiales; f__Lachnospiraceae; g__Dorea; s__formicigenerans	4.801313706	1.46E-21
193968	k__Bacteria; p__Firmicutes; c__Clostridia; o__Clostridiales; f__Ruminococcaceae; g__; s__	5.447677412	2.37E-21
4325096	k__Bacteria; p__Firmicutes; c__Clostridia; o__Clostridiales; f__Lachnospiraceae; g__; s__	5.472877032	2.97E-21
3138798	k__Bacteria; p__Firmicutes; c__Clostridia; o__Clostridiales; f__Veillonellaceae; g__Phascolarctobacterium; s__	5.76492041	5.14E-21
174840	k__Bacteria; p__Firmicutes; c__Clostridia; o__Clostridiales; f__Ruminococcaceae; g__; s__	5.151768908	7.04E-21
180999	k__Bacteria; p__Firmicutes; c__Clostridia; o__Clostridiales; f__Lachnospiraceae; g__; s__	5.310057404	3.83E-20
4472174	k__Bacteria; p__Firmicutes; c__Clostridia; o__Clostridiales; f__Lachnospiraceae; g__[Ruminococcus]; s__	5.211719863	6.86E-20
4472202	k__Bacteria; p__Firmicutes; c__Clostridia; o__Clostridiales; f__Lachnospiraceae; g__[Ruminococcus]; s__	5.11784575	1.27E-19
242298	k__Bacteria; p__Firmicutes; c__Clostridia; o__Clostridiales; f__Peptostreptococcaceae; g__; s__	-3.559808182	1.27E-19
798581	k__Bacteria; p__Firmicutes; c__Clostridia; o__Clostridiales; f__Ruminococcaceae; g__Ruminococcus; s__bromii	5.154423354	1.97E-19
4409730	k__Bacteria; p__Firmicutes; c__Clostridia; o__Clostridiales; f__Peptostreptococcaceae; g__; s__	5.081878871	2.11E-19
324882	k__Bacteria; p__Firmicutes; c__Clostridia; o__Clostridiales; f__[Mogibacteriaceae]; g__; s__	-5.338931999	1.52E-18
4465072	k__Bacteria; p__Firmicutes; c__Clostridia; o__Clostridiales; f__Ruminococcaceae; g__; s__	5.589416004	3.02E-18
4463532	k__Bacteria; p__Firmicutes; c__Clostridia; o__Clostridiales; f__; g__; s__	4.650691777	1.99E-17
2617854	k__Bacteria; p__Bacteroidetes; c__Bacteroidia; o__Bacteroidales; f__Rikenellaceae; g__; s__	5.061701378	2.50E-17
4371061	k__Bacteria; p__Firmicutes; c__Clostridia; o__Clostridiales; f__; g__; s__	4.329563162	2.70E-17
4480529	k__Bacteria; p__Firmicutes; c__Clostridia; o__Clostridiales; f__Lachnospiraceae; g__Dorea; s__	4.925973305	3.82E-17
187924	k__Bacteria; p__Firmicutes; c__Clostridia; o__Clostridiales; f__Ruminococcaceae; g__; s__	5.074368148	5.29E-17
4405104	k__Bacteria; p__Firmicutes; c__Clostridia; o__Clostridiales; f__Lachnospiraceae; g__Coprococcus; s__	5.185014985	5.29E-17
173863	k__Bacteria; p__Firmicutes; c__Clostridia; o__Clostridiales; f__Ruminococcaceae; g__; s__	4.455438183	7.71E-17
782953	k__Bacteria; p__Proteobacteria; c__Gammaproteobacteria; o__Enterobacteriales; f__Enterobacteriaceae; g__; s__	-4.66574003	8.80E-17
4347159	k__Bacteria; p__Actinobacteria; c__Actinobacteria; o__Bifidobacteriales; f__Bifidobacteriaceae; g__Bifidobacterium; s__adolescentis	5.150685577	1.31E-16
664205	k__Bacteria; p__Firmicutes; c__Bacilli; o__Bacillales; f__Bacillaceae; g__Bacillus; s__coagulans	-3.271820342	2.06E-16
190913	k__Bacteria; p__Bacteroidetes; c__Bacteroidia; o__Bacteroidales; f__Bacteroidaceae; g__Bacteroides; s__	4.73134207	2.21E-16
4479443	k__Bacteria; p__Firmicutes; c__Clostridia; o__Clostridiales; f__Lachnospiraceae; g__; s__	4.422727706	2.54E-16
2407149	k__Bacteria; p__Firmicutes; c__Clostridia; o__Clostridiales; f__Lachnospiraceae; g__Lachnospira; s__	5.101507447	2.54E-16
4469007	k__Bacteria; p__Firmicutes; c__Clostridia; o__Clostridiales; f__; g__; s__	4.281009184	3.18E-16
4456027	k__Bacteria; p__Firmicutes; c__Clostridia; o__Clostridiales; f__Lachnospiraceae; g__; s__	4.420587527	4.04E-16
730906	k__Bacteria; p__Firmicutes; c__Clostridia; o__Clostridiales; f__Ruminococcaceae; g__; s__	3.763791301	5.66E-16
4401580	k__Bacteria; p__Bacteroidetes; c__Bacteroidia; o__Bacteroidales; f__Bacteroidaceae; g__Bacteroides; s__	4.41162438	7.99E-16
292735	k__Bacteria; p__Firmicutes; c__Clostridia; o__Clostridiales; f__Lachnospiraceae; g__Blautia; s__	-5.342863784	1.21E-15
4447950	k__Bacteria; p__Bacteroidetes; c__Bacteroidia; o__Bacteroidales; f__Bacteroidaceae; g__Bacteroides; s__	6.422796276	1.64E-15
188735	k__Bacteria; p__Bacteroidetes; c__Bacteroidia; o__Bacteroidales; f__Bacteroidaceae; g__Bacteroides; s__	4.289862157	2.83E-15
179826	k__Bacteria; p__Firmicutes; c__Clostridia; o__Clostridiales; f__Ruminococcaceae; g__; s__	4.289582834	3.01E-15
4232045	k__Bacteria; p__Bacteroidetes; c__Bacteroidia; o__Bacteroidales; f__Bacteroidaceae; g__Bacteroides; s__	4.356516613	3.12E-15
187504	k__Bacteria; p__Firmicutes; c__Clostridia; o__Clostridiales; f__Ruminococcaceae; g__; s__	4.44696371	3.20E-15
2532173	k__Bacteria; p__Firmicutes; c__Clostridia; o__Clostridiales; f__Lachnospiraceae; g__Roseburia; s__faecis	4.017000924	7.25E-15
4385326	k__Bacteria; p__Firmicutes; c__Clostridia; o__Clostridiales; f__; g__; s__	4.552453706	7.43E-15
182886	k__Bacteria; p__Bacteroidetes; c__Bacteroidia; o__Bacteroidales; f__Bacteroidaceae; g__Bacteroides; s__uniformis	4.118069037	8.69E-15
125624	k__Bacteria; p__Firmicutes; c__Clostridia; o__Clostridiales; f__Lachnospiraceae; g__; s__	4.673691924	8.69E-15
4342104	k__Bacteria; p__Firmicutes; c__Clostridia; o__Clostridiales; f__Ruminococcaceae; g__Anaerotruncus; s__	-6.580964161	2.63E-14
4465907	k__Bacteria; p__Firmicutes; c__Clostridia; o__Clostridiales; f__Lachnospiraceae; g__Blautia; s__	4.68844226	2.72E-14
361966	k__Bacteria; p__Firmicutes; c__Clostridia; o__Clostridiales; f__Ruminococcaceae; g__Faecalibacterium; s__prausnitzii	4.418242459	2.81E-14
3275562	k__Bacteria; p__Firmicutes; c__Clostridia; o__Clostridiales; f__Lachnospiraceae; g__; s__	4.393754831	6.00E-14
1504042	k__Bacteria; p__Firmicutes; c__Clostridia; o__Clostridiales; f__Ruminococcaceae; g__Oscillospira; s__	3.825414987	9.17E-14
4480359	k__Bacteria; p__Firmicutes; c__Clostridia; o__Clostridiales; f__Ruminococcaceae; g__; s__	4.929347425	1.17E-13
357046	k__Bacteria; p__Bacteroidetes; c__Bacteroidia; o__Bacteroidales; f__Rikenellaceae; g__; s__	4.189775631	1.32E-13

4396297	k__Bacteria; p__Firmicutes; c__Clostridia; o__Clostridiales; f__Lachnospiraceae; g__; s__	4.154379181	1.70E-13
2497335	k__Bacteria; p__Bacteroidetes; c__Bacteroidia; o__Bacteroidales; f__Porphyromonadaceae; g__Parabacteroides; s__distasonis	4.525201921	2.04E-13
180414	k__Bacteria; p__Firmicutes; c__Clostridia; o__Clostridiales; f__Lachnospiraceae; g__Blautia; s__	3.992653706	2.12E-13
4472091	k__Bacteria; p__Firmicutes; c__Clostridia; o__Clostridiales; f__Ruminococcaceae; g__Ruminococcus; s__	4.230475295	3.63E-13
4359797	k__Bacteria; p__Firmicutes; c__Clostridia; o__Clostridiales; f__Lachnospiraceae; g__Blautia; s__	3.800326634	3.77E-13
4476780	k__Bacteria; p__Bacteroidetes; c__Bacteroidia; o__Bacteroidales; f__Rikenellaceae; g__; s__	4.366231291	5.09E-13
4102199	k__Bacteria; p__Firmicutes; c__Clostridia; o__Clostridiales; f__Ruminococcaceae; g__; s__	4.171985559	7.49E-13
176269	k__Bacteria; p__Firmicutes; c__Clostridia; o__Clostridiales; f__Lachnospiraceae; g__Lachnospira; s__	4.459144515	1.00E-12
184925	k__Bacteria; p__Firmicutes; c__Clostridia; o__Clostridiales; f__; g__; s__	4.483661768	1.05E-12
4473788	k__Bacteria; p__Firmicutes; c__Clostridia; o__Clostridiales; f__Lachnospiraceae; g__; s__	3.955317715	1.90E-12
193679	k__Bacteria; p__Firmicutes; c__Clostridia; o__Clostridiales; f__Ruminococcaceae; g__; s__	3.842486054	2.43E-12
4472399	k__Bacteria; p__Firmicutes; c__Clostridia; o__Clostridiales; f__Lachnospiraceae; g__; s__	3.824252038	3.03E-12
4364405	k__Bacteria; p__Firmicutes; c__Clostridia; o__Clostridiales; f__Ruminococcaceae; g__; s__	4.963538947	3.95E-12
4451899	k__Bacteria; p__Firmicutes; c__Clostridia; o__Clostridiales; f__Lachnospiraceae; g__; s__	4.241238473	4.53E-12
178082	k__Bacteria; p__Firmicutes; c__Clostridia; o__Clostridiales; f__Lachnospiraceae; g__Lachnospira; s__	4.341384489	4.53E-12
4331360	k__Bacteria; p__Firmicutes; c__Clostridia; o__Clostridiales; f__Lachnospiraceae; g__; s__	3.555289354	4.94E-12
4217963	k__Bacteria; p__Firmicutes; c__Clostridia; o__Clostridiales; f__Lachnospiraceae; g__; s__	3.882760241	4.94E-12
191251	k__Bacteria; p__Bacteroidetes; c__Bacteroidia; o__Bacteroidales; f__Porphyromonadaceae; g__Parabacteroides; s__	3.678289368	5.67E-12
4301511	k__Bacteria; p__Firmicutes; c__Clostridia; o__Clostridiales; f__; g__; s__	3.960096891	6.66E-12
3856408	k__Bacteria; p__Firmicutes; c__Clostridia; o__Clostridiales; f__Lachnospiraceae; g__; s__	3.776310719	1.02E-11
177581	k__Bacteria; p__Firmicutes; c__Clostridia; o__Clostridiales; f__Lachnospiraceae; g__; s__	3.486525784	1.23E-11
195651	k__Bacteria; p__Firmicutes; c__Clostridia; o__Clostridiales; f__Ruminococcaceae; g__; s__	3.582085762	1.45E-11
3924627	k__Bacteria; p__Firmicutes; c__Clostridia; o__Clostridiales; f__Lachnospiraceae; g__; s__	3.550344357	1.64E-11
4480244	k__Bacteria; p__Firmicutes; c__Clostridia; o__Clostridiales; f__Veillonellaceae; g__Dialister; s__	5.300367373	1.98E-11
183698	k__Bacteria; p__Firmicutes; c__Clostridia; o__Clostridiales; f__Lachnospiraceae; g__; s__	3.268056464	1.98E-11
178462	k__Bacteria; p__Firmicutes; c__Clostridia; o__Clostridiales; f__Lachnospiraceae; g__Blautia; s__	-5.128635707	3.11E-11
4318125	k__Bacteria; p__Firmicutes; c__Clostridia; o__Clostridiales; f__Lachnospiraceae; g__; s__	3.742782022	3.11E-11
4403632	k__Bacteria; p__Firmicutes; c__Clostridia; o__Clostridiales; f__Lachnospiraceae; g__Coprococcus; s__	3.692047372	3.69E-11
2438203	k__Bacteria; p__Firmicutes; c__Clostridia; o__Clostridiales; f__Lachnospiraceae; g__Roseburia; s__	3.685666235	4.37E-11
4433823	k__Bacteria; p__Bacteroidetes; c__Bacteroidia; o__Bacteroidales; f__Bacteroidaceae; g__Bacteroides; s__fragilis	4.329167337	4.37E-11
4354486	k__Bacteria; p__Firmicutes; c__Clostridia; o__Clostridiales; f__; g__; s__	3.367698576	4.96E-11
302049	k__Bacteria; p__Firmicutes; c__Clostridia; o__Clostridiales; f__Lachnospiraceae; g__Blautia; s__	-4.333566301	5.41E-11
4448492	k__Bacteria; p__Firmicutes; c__Clostridia; o__Clostridiales; f__Lachnospiraceae; g__; s__	3.18172126	8.59E-11
4254528	k__Bacteria; p__Firmicutes; c__Clostridia; o__Clostridiales; f__Lachnospiraceae; g__; s__	3.311338818	9.32E-11
2943548	k__Bacteria; p__Firmicutes; c__Clostridia; o__Clostridiales; f__Ruminococcaceae; g__Ruminococcus; s__	3.618863379	9.78E-11
176115	k__Bacteria; p__Firmicutes; c__Clostridia; o__Clostridiales; f__Ruminococcaceae; g__Faecalibacterium; s__prausnitzii	3.676596202	1.34E-10
3537197	k__Bacteria; p__Firmicutes; c__Clostridia; o__Clostridiales; f__Lachnospiraceae; g__Anaerostipes; s__	3.385253708	1.40E-10
195105	k__Bacteria; p__Firmicutes; c__Clostridia; o__Clostridiales; f__Lachnospiraceae; g__Roseburia; s__faecis	3.460907912	1.42E-10
4278525	k__Bacteria; p__Bacteroidetes; c__Bacteroidia; o__Bacteroidales; f__Bacteroidaceae; g__Bacteroides; s__	3.298314762	1.61E-10
15728	k__Bacteria; p__Firmicutes; c__Erysipelotrichi; o__Erysipelotrichales; f__Erysipelotrichaceae; g__Holdemania; s__	3.264341587	1.62E-10
4473509	k__Bacteria; p__Firmicutes; c__Clostridia; o__Clostridiales; f__Lachnospiraceae; g__; s__	3.56876603	1.80E-10
367176	k__Bacteria; p__Firmicutes; c__Clostridia; o__Clostridiales; f__Ruminococcaceae; g__Oscillospira; s__	-5.425535728	1.94E-10
198190	k__Bacteria; p__Bacteroidetes; c__Bacteroidia; o__Bacteroidales; f__Porphyromonadaceae; g__Parabacteroides; s__distasonis	3.310042107	2.02E-10
4437359	k__Bacteria; p__Firmicutes; c__Clostridia; o__Clostridiales; f__Ruminococcaceae; g__Oscillospira; s__	3.694012541	2.83E-10
317677	k__Bacteria; p__Firmicutes; c__Clostridia; o__Clostridiales; f__Ruminococcaceae; g__; s__	3.401447159	3.00E-10
182089	k__Bacteria; p__Firmicutes; c__Clostridia; o__Clostridiales; f__Ruminococcaceae; g__; s__	3.534718357	3.85E-10
173876	k__Bacteria; p__Firmicutes; c__Clostridia; o__Clostridiales; f__; g__; s__	4.465749177	4.88E-10

308544	k__Bacteria; p__Firmicutes; c__Clostridia; o__Clostridiales; f__Ruminococcaceae; g__; s__	-4.034277254	6.02E-10
357261	k__Bacteria; p__Firmicutes; c__Clostridia; o__Clostridiales; f__Ruminococcaceae; g__Ruminococcus; s__	-4.483342081	8.09E-10
4444262	k__Bacteria; p__Firmicutes; c__Clostridia; o__Clostridiales; f__Lachnospiraceae; g__; s__	4.118878	8.09E-10
146554	k__Bacteria; p__Firmicutes; c__Clostridia; o__Clostridiales; f__Ruminococcaceae; g__Ruminococcus; s__	-4.731587609	8.82E-10
182036	k__Bacteria; p__Firmicutes; c__Clostridia; o__Clostridiales; f__Ruminococcaceae; g__; s__	3.368121639	1.13E-09
180155	k__Bacteria; p__Firmicutes; c__Clostridia; o__Clostridiales; f__Ruminococcaceae; g__; s__	3.363251114	1.21E-09
324894	k__Bacteria; p__Firmicutes; c__Clostridia; o__Clostridiales; f__Ruminococcaceae; g__; s__	-4.256928832	1.70E-09
179785	k__Bacteria; p__Firmicutes; c__Clostridia; o__Clostridiales; f__; g__; s__	3.304840367	1.70E-09
2307779	k__Bacteria; p__Firmicutes; c__Clostridia; o__Clostridiales; f__Ruminococcaceae; g__Oscillospira; s__	3.351110508	1.77E-09
2017729	k__Bacteria; p__Firmicutes; c__Clostridia; o__Clostridiales; f__Lachnospiraceae; g__; s__	3.133230555	2.46E-09
162623	k__Bacteria; p__Firmicutes; c__Clostridia; o__Clostridiales; f__Lachnospiraceae; g__Roseburia; s__	2.937544452	2.83E-09
4396292	k__Bacteria; p__Firmicutes; c__Clostridia; o__Clostridiales; f__Ruminococcaceae; g__; s__	3.385958093	3.37E-09
195556	k__Bacteria; p__Firmicutes; c__Clostridia; o__Clostridiales; f__Ruminococcaceae; g__; s__	3.350213092	4.19E-09
4437362	k__Bacteria; p__Bacteroidetes; c__Bacteroidia; o__Bacteroidales; f__Bacteroidaceae; g__Bacteroides; s__	3.535154776	5.00E-09
350697	k__Bacteria; p__Firmicutes; c__Clostridia; o__Clostridiales; f__Peptostreptococcaceae; g__; s__	-2.428582142	5.26E-09
313593	k__Bacteria; p__Firmicutes; c__Clostridia; o__Clostridiales; f__Lachnospiraceae; g__Roseburia; s__	3.235504344	6.39E-09
3943186	k__Bacteria; p__Firmicutes; c__Clostridia; o__Clostridiales; f__Lachnospiraceae; g__Lachnobacterium; s__	3.662229889	7.30E-09
189524	k__Bacteria; p__Firmicutes; c__Clostridia; o__Clostridiales; f__Ruminococcaceae; g__; s__	3.382762633	7.47E-09
192983	k__Bacteria; p__Firmicutes; c__Clostridia; o__Clostridiales; f__Lachnospiraceae; g__; s__	3.086442239	7.51E-09
216111	k__Bacteria; p__Firmicutes; c__Clostridia; o__Clostridiales; f__Lachnospiraceae; g__; s__	3.422116049	8.03E-09
186997	k__Bacteria; p__Firmicutes; c__Clostridia; o__Clostridiales; f__Lachnospiraceae; g__; s__	-4.060613641	8.51E-09
199534	k__Bacteria; p__Bacteroidetes; c__Bacteroidia; o__Bacteroidales; f__Rikenellaceae; g__; s__	3.149384118	9.19E-09
181204	k__Bacteria; p__Firmicutes; c__Clostridia; o__Clostridiales; f__Ruminococcaceae; g__; s__	3.137887658	9.76E-09
174489	k__Bacteria; p__Firmicutes; c__Clostridia; o__Clostridiales; f__Lachnospiraceae; g__; s__	3.225004897	1.33E-08
1077373	k__Bacteria; p__Bacteroidetes; c__Bacteroidia; o__Bacteroidales; f__Prevotellaceae; g__Prevotella; s__	3.565966476	1.33E-08
4428676	k__Bacteria; p__Firmicutes; c__Clostridia; o__Clostridiales; f__Lachnospiraceae; g__Coprococcus; s__	3.17286207	1.42E-08
4474255	k__Bacteria; p__Proteobacteria; c__Betaproteobacteria; o__Burkholderiales; f__Alcaligenaceae; g__Sutterella; s__	3.211805488	1.70E-08
4408801	k__Bacteria; p__Firmicutes; c__Clostridia; o__Clostridiales; f__Ruminococcaceae; g__Oscillospira; s__	3.187015009	1.75E-08
336710	k__Bacteria; p__Bacteroidetes; c__Bacteroidia; o__Bacteroidales; f__Bacteroidaceae; g__Bacteroides; s__	3.064502006	1.78E-08
4306262	k__Bacteria; p__Verrucomicrobia; c__Verrucomicrobiae; o__Verrucomicrobiales; f__Verrucomicrobiaceae; g__Akkermansia; s__muciniphila	3.610669592	1.92E-08
181862	k__Bacteria; p__Firmicutes; c__Clostridia; o__Clostridiales; f__Ruminococcaceae; g__; s__	3.234268969	1.94E-08
592616	k__Bacteria; p__Firmicutes; c__Erysipelotrichi; o__Erysipelotrichales; f__Erysipelotrichaceae; g__; s__	3.237516473	2.07E-08
248902	k__Bacteria; p__Firmicutes; c__Bacilli; o__Turicibacterales; f__Turicibacteraceae; g__Turicibacter; s__	-3.694875051	2.39E-08
3530697	k__Bacteria; p__Firmicutes; c__Clostridia; o__Clostridiales; f__Ruminococcaceae; g__; s__	3.088548414	2.44E-08
194297	k__Bacteria; p__Firmicutes; c__Clostridia; o__Clostridiales; f__Ruminococcaceae; g__Ruminococcus; s__	3.892243391	2.85E-08
177037	k__Bacteria; p__Firmicutes; c__Clostridia; o__Clostridiales; f__Lachnospiraceae; g__Blautia; s__	2.847241358	3.35E-08
2979308	k__Bacteria; p__Firmicutes; c__Clostridia; o__Clostridiales; f__Ruminococcaceae; g__Ruminococcus; s__	2.824053736	3.45E-08
1096610	k__Bacteria; p__Firmicutes; c__Clostridia; o__Clostridiales; f__[Tissierellaceae]; g__Finegoldia; s__	3.439483293	3.52E-08
4458576	k__Bacteria; p__Firmicutes; c__Clostridia; o__Clostridiales; f__Lachnospiraceae; g__; s__	3.287082996	3.57E-08
193863	k__Bacteria; p__Firmicutes; c__Clostridia; o__Clostridiales; f__Lachnospiraceae; g__; s__	2.864686093	4.14E-08
4309301	k__Bacteria; p__Firmicutes; c__Bacilli; o__Lactobacillales; f__Streptococcaceae; g__Streptococcus; s__	-2.677519395	5.19E-08
190675	k__Bacteria; p__Firmicutes; c__Clostridia; o__Clostridiales; f__Ruminococcaceae; g__Faecalibacterium; s__prausnitzii	2.750597517	5.19E-08
4483963	k__Bacteria; p__Bacteroidetes; c__Bacteroidia; o__Bacteroidales; f__Bacteroidaceae; g__Bacteroides; s__	3.667264218	5.46E-08
238205	k__Bacteria; p__Firmicutes; c__Clostridia; o__Clostridiales; f__Clostridiaceae; g__Clostridium; s__butyricum	-2.80712847	5.79E-08
3302038	k__Bacteria; p__Firmicutes; c__Clostridia; o__Clostridiales; f__Ruminococcaceae; g__Oscillospira; s__	3.59707011	5.83E-08
3794053	k__Bacteria; p__Firmicutes; c__Clostridia; o__Clostridiales; f__Lachnospiraceae; g__Dorea; s__	2.846741443	7.07E-08
188127	k__Bacteria; p__Firmicutes; c__Clostridia; o__Clostridiales; f__; g__; s__	2.849559184	8.81E-08

319275	k__Bacteria; p__Firmicutes; c__Clostridia; o__Clostridiales; f__Ruminococcaceae; g__Faecalibacterium; s__prausnitzii	3.234601815	9.53E-08
4383953	k__Bacteria; p__Firmicutes; c__Clostridia; o__Clostridiales; f__Clostridiaceae; g__; s__	-3.19940381	9.87E-08
4153054	k__Bacteria; p__Firmicutes; c__Clostridia; o__Clostridiales; f__Ruminococcaceae; g__; s__	3.41640181	9.91E-08
4357811	k__Bacteria; p__Bacteroidetes; c__Bacteroidia; o__Bacteroidales; f__Bacteroidaceae; g__Bacteroides; s__	2.910361859	9.93E-08
4425214	k__Bacteria; p__Firmicutes; c__Bacilli; o__Lactobacillales; f__Streptococcaceae; g__Streptococcus; s__	2.763103368	9.96E-08
4468466	k__Bacteria; p__Firmicutes; c__Clostridia; o__Clostridiales; f__Ruminococcaceae; g__; s__	3.25301822	1.11E-07
182052	k__Bacteria; p__Bacteroidetes; c__Bacteroidia; o__Bacteroidales; f__Bacteroidaceae; g__Bacteroides; s__	3.435369345	1.15E-07
190676	k__Bacteria; p__Firmicutes; c__Clostridia; o__Clostridiales; f__Ruminococcaceae; g__Oscillospira; s__	3.161995327	1.25E-07
176077	k__Bacteria; p__Firmicutes; c__Clostridia; o__Clostridiales; f__; g__; s__	2.84197239	1.37E-07
179358	k__Bacteria; p__Firmicutes; c__Clostridia; o__Clostridiales; f__Ruminococcaceae; g__; s__	2.785007145	1.40E-07
230232	k__Bacteria; p__Firmicutes; c__Clostridia; o__Clostridiales; f__Lachnospiraceae; g__Dorea; s__	2.821154785	1.46E-07
340219	k__Bacteria; p__Firmicutes; c__Clostridia; o__Clostridiales; f__Ruminococcaceae; g__Faecalibacterium; s__prausnitzii	2.725566245	1.48E-07
181756	k__Bacteria; p__Firmicutes; c__Clostridia; o__Clostridiales; f__Lachnospiraceae; g__Blautia; s__	2.502853934	1.52E-07
366352	k__Bacteria; p__Firmicutes; c__Clostridia; o__Clostridiales; f__Ruminococcaceae; g__; s__	-3.52101104	1.67E-07
3236435	k__Bacteria; p__Firmicutes; c__Clostridia; o__Clostridiales; f__Ruminococcaceae; g__; s__	3.334538737	1.77E-07
318970	k__Bacteria; p__Firmicutes; c__Clostridia; o__Clostridiales; f__Lachnospiraceae; g__Blautia; s__	-3.188571844	1.84E-07
194597	k__Bacteria; p__Firmicutes; c__Clostridia; o__Clostridiales; f__; g__; s__	3.489593958	1.87E-07
170652	k__Bacteria; p__Firmicutes; c__Clostridia; o__Clostridiales; f__Clostridiaceae; g__; s__	-2.746679556	1.87E-07
4425663	k__Bacteria; p__Firmicutes; c__Clostridia; o__Clostridiales; f__; g__; s__	3.448909118	1.89E-07
4361727	k__Bacteria; p__Bacteroidetes; c__Bacteroidia; o__Bacteroidales; f__Bacteroidaceae; g__Bacteroides; s__	2.673979785	1.98E-07
841635	k__Bacteria; p__Proteobacteria; c__Betaproteobacteria; o__Burkholderiales; f__Alcaligenaceae; g__Sutterella; s__	3.577035044	2.35E-07
2688035	k__Bacteria; p__Firmicutes; c__Clostridia; o__Clostridiales; f__Lachnospiraceae; g__; s__	2.814570672	2.48E-07
359872	k__Bacteria; p__Proteobacteria; c__Deltaproteobacteria; o__Desulfovibrionales; f__Desulfovibrionaceae; g__Bilophila; s__	3.040554702	2.64E-07
175336	k__Bacteria; p__Firmicutes; c__Clostridia; o__Clostridiales; f__Ruminococcaceae; g__Oscillospira; s__	2.998505748	3.02E-07
2018038	k__Bacteria; p__Firmicutes; c__Clostridia; o__Clostridiales; f__Ruminococcaceae; g__; s__	3.005774646	3.49E-07
120281	k__Bacteria; p__Firmicutes; c__Clostridia; o__Clostridiales; f__Lachnospiraceae; g__Blautia; s__producta	3.036130752	3.67E-07
4481195	k__Bacteria; p__Firmicutes; c__Clostridia; o__Clostridiales; f__Lachnospiraceae; g__; s__	2.482970163	3.70E-07
186022	k__Bacteria; p__Firmicutes; c__Clostridia; o__Clostridiales; f__Lachnospiraceae; g__Blautia; s__	-3.24734528	3.90E-07
4469032	k__Bacteria; p__Firmicutes; c__Bacilli; o__Lactobacillales; f__; g__; s__	-2.765522424	4.01E-07
182054	k__Bacteria; p__Firmicutes; c__Clostridia; o__Clostridiales; f__Lachnospiraceae; g__Coprococcus; s__	2.583634724	4.19E-07
175148	k__Bacteria; p__Firmicutes; c__Clostridia; o__Clostridiales; f__Ruminococcaceae; g__; s__	2.661986217	4.30E-07
198511	k__Bacteria; p__Firmicutes; c__Clostridia; o__Clostridiales; f__Lachnospiraceae; g__; s__	2.866204461	4.84E-07
306299	k__Bacteria; p__Firmicutes; c__Clostridia; o__Clostridiales; f__Lachnospiraceae; g__; s__	-2.56243339	6.06E-07
175180	k__Bacteria; p__Firmicutes; c__Clostridia; o__Clostridiales; f__; g__; s__	2.456566583	6.06E-07
365536	k__Bacteria; p__Firmicutes; c__Clostridia; o__Clostridiales; f__Ruminococcaceae; g__Oscillospira; s__	2.514462202	6.06E-07
348009	k__Bacteria; p__Firmicutes; c__Clostridia; o__Clostridiales; f__Ruminococcaceae; g__Oscillospira; s__	3.154742602	6.37E-07
2368865	k__Bacteria; p__Firmicutes; c__Clostridia; o__Clostridiales; f__; g__; s__	3.32161353	6.37E-07
186732	k__Bacteria; p__Firmicutes; c__Clostridia; o__Clostridiales; f__Ruminococcaceae; g__; s__	-3.244009801	6.60E-07
4476604	k__Bacteria; p__Firmicutes; c__Clostridia; o__Clostridiales; f__Lachnospiraceae; g__[Ruminococcus]; s__gnavus	2.936461391	6.80E-07
163494	k__Bacteria; p__Firmicutes; c__Clostridia; o__Clostridiales; f__Lachnospiraceae; g__; s__	2.654346675	7.24E-07
575407	k__Bacteria; p__Verrucomicrobia; c__Verrucomicrobiae; o__Verrucomicrobiales; f__Verrucomicrobiaceae; g__Akkermansia; s__muciniphila	3.256247913	7.29E-07
197624	k__Bacteria; p__Firmicutes; c__Clostridia; o__Clostridiales; f__Ruminococcaceae; g__; s__	2.404747349	7.35E-07
198646	k__Bacteria; p__Firmicutes; c__Clostridia; o__Clostridiales; f__Lachnospiraceae; g__Blautia; s__	-3.134858202	7.92E-07
1919007	k__Bacteria; p__Bacteroidetes; c__Bacteroidia; o__Bacteroidales; f__Bacteroidaceae; g__Bacteroides; s__	2.5432145	7.92E-07
4459196	k__Bacteria; p__Firmicutes; c__Clostridia; o__Clostridiales; f__Lachnospiraceae; g__Lachnospira; s__	2.906061291	7.92E-07
157338	k__Bacteria; p__Firmicutes; c__Clostridia; o__Clostridiales; f__; g__; s__	3.224123926	8.01E-07
185034	k__Bacteria; p__Firmicutes; c__Clostridia; o__Clostridiales; f__; g__; s__	2.816831263	8.33E-07

4446320	k__Bacteria; p__Firmicutes; c__Clostridia; o__Clostridiales; f__Clostridiaceae; g__; s__	2.912666933	8.44E-07
177697	k__Bacteria; p__Firmicutes; c__Clostridia; o__Clostridiales; f__Ruminococcaceae; g__; s__	2.766311117	8.95E-07
186955	k__Bacteria; p__Firmicutes; c__Clostridia; o__Clostridiales; f__Lachnospiraceae; g__; s__	2.509142923	9.08E-07
157453	k__Bacteria; p__Firmicutes; c__Clostridia; o__Clostridiales; f__Ruminococcaceae; g__; s__	2.899850144	9.08E-07
4437368	k__Bacteria; p__Firmicutes; c__Clostridia; o__Clostridiales; f__Lachnospiraceae; g__; s__	2.416528253	9.13E-07
330469	k__Bacteria; p__Firmicutes; c__Clostridia; o__Clostridiales; f__Ruminococcaceae; g__; s__	2.548734395	9.32E-07
195950	k__Bacteria; p__Firmicutes; c__Clostridia; o__Clostridiales; f__Ruminococcaceae; g__; s__	3.058076668	9.32E-07
4448928	k__Bacteria; p__Firmicutes; c__Clostridia; o__Clostridiales; f__Clostridiaceae; g__Clostridium; s__	-2.56456257	1.09E-06
193667	k__Bacteria; p__Firmicutes; c__Clostridia; o__Clostridiales; f__Ruminococcaceae; g__Faecalibacterium; s__	2.207434305	1.13E-06
212532	k__Bacteria; p__Firmicutes; c__Clostridia; o__Clostridiales; f__Ruminococcaceae; g__; s__	2.433239407	1.15E-06
177230	k__Bacteria; p__Firmicutes; c__Clostridia; o__Clostridiales; f__Lachnospiraceae; g__; s__	2.387972334	1.20E-06
184561	k__Bacteria; p__Firmicutes; c__Clostridia; o__Clostridiales; f__Lachnospiraceae; g__Blautia; s__	2.588598058	1.25E-06
196724	k__Bacteria; p__Firmicutes; c__Clostridia; o__Clostridiales; f__Lachnospiraceae; g__Blautia; s__	2.329805983	1.30E-06
4339144	k__Bacteria; p__Bacteroidetes; c__Bacteroidia; o__Bacteroidales; f__[Odoribacteraceae]; g__Butyricimonas; s__	3.117465088	1.33E-06
544996	k__Bacteria; p__Firmicutes; c__Clostridia; o__Clostridiales; f__Ruminococcaceae; g__Oscillospira; s__	2.869901377	1.34E-06
545299	k__Bacteria; p__Fusobacteria; c__Fusobacteriia; o__Fusobacteriales; f__Fusobacteriaceae; g__Fusobacterium; s__	-2.299237197	1.52E-06
4466707	k__Bacteria; p__Firmicutes; c__Clostridia; o__Clostridiales; f__Lachnospiraceae; g__Coprococcus; s__	2.792497987	1.60E-06
175145	k__Bacteria; p__Firmicutes; c__Clostridia; o__Clostridiales; f__Lachnospiraceae; g__; s__	2.369673909	1.94E-06
182073	k__Bacteria; p__Firmicutes; c__Clostridia; o__Clostridiales; f__; g__; s__	2.830350638	1.94E-06
1951826	k__Bacteria; p__Proteobacteria; c__Gammaproteobacteria; o__Enterobacteriales; f__Enterobacteriaceae; g__; s__	3.225158187	1.96E-06
495451	k__Bacteria; p__Bacteroidetes; c__Bacteroidia; o__Bacteroidales; f__Porphyromonadaceae; g__Porphyromonas; s__	2.807337756	2.04E-06
189083	k__Bacteria; p__Bacteroidetes; c__Bacteroidia; o__Bacteroidales; f__Prevotellaceae; g__Prevotella; s__copri	2.785113408	2.05E-06
352034	k__Bacteria; p__Bacteroidetes; c__Bacteroidia; o__Bacteroidales; f__Bacteroidaceae; g__Bacteroides; s__	2.615212689	2.07E-06
176690	k__Bacteria; p__Firmicutes; c__Clostridia; o__Clostridiales; f__Lachnospiraceae; g__Roseburia; s__	2.39206123	2.14E-06
327218	k__Bacteria; p__Firmicutes; c__Clostridia; o__Clostridiales; f__Ruminococcaceae; g__; s__	2.507223676	2.19E-06
4372973	k__Bacteria; p__Firmicutes; c__Clostridia; o__Clostridiales; f__Ruminococcaceae; g__; s__	2.750132537	2.19E-06
363477	k__Bacteria; p__Firmicutes; c__Clostridia; o__Clostridiales; f__Ruminococcaceae; g__; s__	2.312109464	2.32E-06
191412	k__Bacteria; p__Firmicutes; c__Clostridia; o__Clostridiales; f__Lachnospiraceae; g__; s__	2.310320167	2.33E-06
194371	k__Bacteria; p__Firmicutes; c__Clostridia; o__Clostridiales; f__Lachnospiraceae; g__Blautia; s__	-3.131198526	2.36E-06
4482516	k__Bacteria; p__Firmicutes; c__Clostridia; o__Clostridiales; f__Clostridiaceae; g__; s__	2.859504772	2.42E-06
194223	k__Bacteria; p__Firmicutes; c__Clostridia; o__Clostridiales; f__Ruminococcaceae; g__Ruminococcus; s__	-3.2352809	2.48E-06
4414420	k__Bacteria; p__Bacteroidetes; c__Bacteroidia; o__Bacteroidales; f__Bacteroidaceae; g__Bacteroides; s__eggerthii	2.92068171	2.58E-06
4412540	k__Bacteria; p__Firmicutes; c__Clostridia; o__Clostridiales; f__Ruminococcaceae; g__; s__	3.152914738	2.59E-06
4094259	k__Bacteria; p__Firmicutes; c__Clostridia; o__Clostridiales; f__Ruminococcaceae; g__; s__	2.496913941	2.74E-06
179601	k__Bacteria; p__Firmicutes; c__Clostridia; o__Clostridiales; f__Ruminococcaceae; g__; s__	2.928388976	2.80E-06
3203801	k__Bacteria; p__Firmicutes; c__Clostridia; o__Clostridiales; f__Lachnospiraceae; g__; s__	2.403302887	2.83E-06
188753	k__Bacteria; p__Firmicutes; c__Clostridia; o__Clostridiales; f__Lachnospiraceae; g__Blautia; s__	-2.937657373	3.04E-06
188648	k__Bacteria; p__Firmicutes; c__Clostridia; o__Clostridiales; f__Lachnospiraceae; g__; s__	2.274731365	3.15E-06
4425571	k__Bacteria; p__Proteobacteria; c__Gammaproteobacteria; o__Enterobacteriales; f__Enterobacteriaceae; g__; s__	-2.340426411	3.20E-06
178242	k__Bacteria; p__Firmicutes; c__Clostridia; o__Clostridiales; f__Ruminococcaceae; g__; s__	2.819820419	3.20E-06
146564	k__Bacteria; p__Firmicutes; c__Clostridia; o__Clostridiales; f__; g__; s__	3.111109409	3.20E-06
851323	k__Bacteria; p__Bacteroidetes; c__Bacteroidia; o__Bacteroidales; f__Porphyromonadaceae; g__Parabacteroides; s__	3.12884039	3.35E-06
4454531	k__Bacteria; p__Proteobacteria; c__Gammaproteobacteria; o__Enterobacteriales; f__Enterobacteriaceae; g__; s__	3.286544948	3.35E-06
176346	k__Bacteria; p__Firmicutes; c__Clostridia; o__Clostridiales; f__Lachnospiraceae; g__Roseburia; s__	2.185676983	3.39E-06
4356080	k__Bacteria; p__Bacteroidetes; c__Bacteroidia; o__Bacteroidales; f__[Barnesiellaceae]; g__; s__	2.792497075	3.68E-06
581079	k__Bacteria; p__Firmicutes; c__Clostridia; o__Clostridiales; f__Ruminococcaceae; g__Oscillospira; s__	-2.919505841	3.70E-06
4419459	k__Bacteria; p__Firmicutes; c__Clostridia; o__Clostridiales; f__; g__; s__	3.118748879	3.70E-06

174862	k__Bacteria; p__Firmicutes; c__Clostridia; o__Clostridiales; f__Lachnospiraceae; g__; s__	2.324449051	3.71E-06
192015	k__Bacteria; p__Firmicutes; c__Clostridia; o__Clostridiales; f__; g__; s__	2.44597235	3.95E-06
175168	k__Bacteria; p__Firmicutes; c__Clostridia; o__Clostridiales; f__; g__; s__	2.29876985	4.46E-06
198947	k__Bacteria; p__Firmicutes; c__Clostridia; o__Clostridiales; f__Ruminococcaceae; g__; s__	2.229810852	4.61E-06
4359216	k__Bacteria; p__Firmicutes; c__Clostridia; o__Clostridiales; f__Ruminococcaceae; g__Faecalibacterium; s__prausnitzii	2.33488397	4.69E-06
188333	k__Bacteria; p__Firmicutes; c__Clostridia; o__Clostridiales; f__Lachnospiraceae; g__[Ruminococcus]; s__gnavus	2.348747902	5.20E-06
4448331	k__Bacteria; p__Proteobacteria; c__Gammaproteobacteria; o__Enterobacteriales; f__Enterobacteriaceae; g__; s__	1.880447284	5.21E-06
321096	k__Bacteria; p__Firmicutes; c__Clostridia; o__Clostridiales; f__Clostridiaceae; g__; s__	-2.902609481	5.31E-06
187126	k__Bacteria; p__Firmicutes; c__Clostridia; o__Clostridiales; f__Ruminococcaceae; g__; s__	2.358138269	5.44E-06
186463	k__Bacteria; p__Firmicutes; c__Clostridia; o__Clostridiales; f__Ruminococcaceae; g__; s__	-2.973013748	5.80E-06
4332078	k__Bacteria; p__Firmicutes; c__Clostridia; o__Clostridiales; f__Clostridiaceae; g__; s__	2.515309984	6.15E-06
177941	k__Bacteria; p__Firmicutes; c__Clostridia; o__Clostridiales; f__Lachnospiraceae; g__Blautia; s__	2.445580279	6.24E-06
180468	k__Bacteria; p__Firmicutes; c__Clostridia; o__Clostridiales; f__Ruminococcaceae; g__Oscillospira; s__	2.231351113	6.26E-06
188079	k__Bacteria; p__Firmicutes; c__Clostridia; o__Clostridiales; f__Lachnospiraceae; g__Coprococcus; s__	2.512495201	6.29E-06
197004	k__Bacteria; p__Firmicutes; c__Clostridia; o__Clostridiales; f__Lachnospiraceae; g__; s__	-2.514788061	6.29E-06
174288	k__Bacteria; p__Firmicutes; c__Clostridia; o__Clostridiales; f__Ruminococcaceae; g__; s__	2.447537248	6.29E-06
4402903	k__Bacteria; p__Firmicutes; c__Clostridia; o__Clostridiales; f__; g__; s__	2.972640338	6.67E-06
178686	k__Bacteria; p__Firmicutes; c__Clostridia; o__Clostridiales; f__Lachnospiraceae; g__Coprococcus; s__	2.582296023	7.30E-06
4429335	k__Bacteria; p__Firmicutes; c__Clostridia; o__Clostridiales; f__[Tissierellaceae]; g__Peptoniphilus; s__	2.848248956	7.30E-06
187883	k__Bacteria; p__Firmicutes; c__Clostridia; o__Clostridiales; f__Ruminococcaceae; g__; s__	2.297101516	7.40E-06
354574	k__Bacteria; p__Proteobacteria; c__Deltaproteobacteria; o__Desulfovibrionales; f__Desulfovibrionaceae; g__Bilophila; s__	2.955297033	7.60E-06
182196	k__Bacteria; p__Firmicutes; c__Clostridia; o__Clostridiales; f__; g__; s__	2.426207865	7.73E-06
211706	k__Bacteria; p__Bacteroidetes; c__Bacteroidia; o__Bacteroidales; f__Bacteroidaceae; g__Bacteroides; s__	2.211163373	8.03E-06
3756485	k__Bacteria; p__Firmicutes; c__Clostridia; o__Clostridiales; f__; g__; s__	2.260388433	8.15E-06
258375	k__Bacteria; p__Firmicutes; c__Clostridia; o__Clostridiales; f__Veillonellaceae; g__Dialister; s__	2.647983843	8.30E-06
178713	k__Bacteria; p__Firmicutes; c__Clostridia; o__Clostridiales; f__Ruminococcaceae; g__; s__	2.880626182	8.50E-06
2949328	k__Bacteria; p__Bacteroidetes; c__Bacteroidia; o__Bacteroidales; f__Bacteroidaceae; g__Bacteroides; s__	2.431689619	8.77E-06
874462	k__Bacteria; p__Bacteroidetes; c__Bacteroidia; o__Bacteroidales; f__Porphyromonadaceae; g__Porphyromonas; s__	2.584534073	8.85E-06
4415390	k__Bacteria; p__Firmicutes; c__Clostridia; o__Clostridiales; f__Lachnospiraceae; g__; s__	2.93822001	8.94E-06
175761	k__Bacteria; p__Firmicutes; c__Clostridia; o__Clostridiales; f__Ruminococcaceae; g__; s__	2.886143023	9.07E-06
4481613	k__Bacteria; p__Actinobacteria; c__Coriobacteriia; o__Coriobacteriales; f__Coriobacteriaceae; g__Collinsella; s__aerofaciens	2.324120529	9.26E-06
197354	k__Bacteria; p__Firmicutes; c__Clostridia; o__Clostridiales; f__Lachnospiraceae; g__Blautia; s__	2.177546735	9.88E-06
145801	k__Bacteria; p__Firmicutes; c__Erysipelotrichi; o__Erysipelotrichales; f__Erysipelotrichaceae; g__; s__	2.462064461	9.88E-06
4451906	k__Bacteria; p__Firmicutes; c__Clostridia; o__Clostridiales; f__Lachnospiraceae; g__; s__	2.451088114	9.88E-06
134786	k__Bacteria; p__Firmicutes; c__Clostridia; o__Clostridiales; f__[Tissierellaceae]; g__Anaerococcus; s__	2.50696932	9.93E-06
194372	k__Bacteria; p__Firmicutes; c__Clostridia; o__Clostridiales; f__Ruminococcaceae; g__; s__	2.207734502	1.10E-05
177618	k__Bacteria; p__Firmicutes; c__Clostridia; o__Clostridiales; f__Lachnospiraceae; g__; s__	2.177925837	1.15E-05
365717	k__Bacteria; p__Firmicutes; c__Clostridia; o__Clostridiales; f__Ruminococcaceae; g__Faecalibacterium; s__prausnitzii	2.323408307	1.30E-05
183604	k__Bacteria; p__Firmicutes; c__Clostridia; o__Clostridiales; f__Lachnospiraceae; g__Blautia; s__	-2.685194206	1.34E-05
3887769	k__Bacteria; p__Bacteroidetes; c__Bacteroidia; o__Bacteroidales; f__Bacteroidaceae; g__Bacteroides; s__	2.104676868	1.42E-05
4439469	k__Bacteria; p__Firmicutes; c__Clostridia; o__Clostridiales; f__Ruminococcaceae; g__; s__	2.252337751	1.44E-05
195029	k__Bacteria; p__Firmicutes; c__Clostridia; o__Clostridiales; f__; g__; s__	2.562921044	1.44E-05
191913	k__Bacteria; p__Firmicutes; c__Clostridia; o__Clostridiales; f__Lachnospiraceae; g__; s__	2.180658183	1.48E-05
179663	k__Bacteria; p__Firmicutes; c__Clostridia; o__Clostridiales; f__Ruminococcaceae; g__; s__	2.040753413	1.51E-05
183071	k__Bacteria; p__Firmicutes; c__Clostridia; o__Clostridiales; f__Ruminococcaceae; g__; s__	2.16442404	1.59E-05
198194	k__Bacteria; p__Firmicutes; c__Clostridia; o__Clostridiales; f__Ruminococcaceae; g__Oscillospira; s__	-3.057806802	1.64E-05
180629	k__Bacteria; p__Firmicutes; c__Clostridia; o__Clostridiales; f__Lachnospiraceae; g__Blautia; s__	-2.550933371	1.64E-05

321902	k__Bacteria; p__Firmicutes; c__Clostridia; o__Clostridiales; f__Lachnospiraceae; g__; s__	2.588790185	1.64E-05
138179	k__Bacteria; p__Bacteroidetes; c__Bacteroidia; o__Bacteroidales; f__Porphyromonadaceae; g__Porphyromonas; s__	2.465995239	1.69E-05
177032	k__Bacteria; p__Firmicutes; c__Clostridia; o__Clostridiales; f__; g__; s__	2.237781449	1.69E-05
173917	k__Bacteria; p__Firmicutes; c__Clostridia; o__Clostridiales; f__Lachnospiraceae; g__; s__	2.178968542	1.74E-05
4449851	k__Bacteria; p__Proteobacteria; c__Gammaproteobacteria; o__Enterobacteriales; f__Enterobacteriaceae; g__; s__	2.281288681	1.74E-05
672440	k__Bacteria; p__Firmicutes; c__Clostridia; o__Clostridiales; f__; g__; s__	2.667753894	1.74E-05
16054	k__Bacteria; p__Firmicutes; c__Clostridia; o__Clostridiales; f__Ruminococcaceae; g__Ruminococcus; s__callidus	2.673147804	1.74E-05
168071	k__Bacteria; p__Firmicutes; c__Clostridia; o__Clostridiales; f__Lachnospiraceae; g__; s__	2.226569305	1.74E-05
4430843	k__Bacteria; p__Bacteroidetes; c__Bacteroidia; o__Bacteroidales; f__Prevotellaceae; g__Prevotella; s__	2.661425247	1.75E-05
190864	k__Bacteria; p__Firmicutes; c__Clostridia; o__Clostridiales; f__; g__; s__	2.052047226	1.77E-05
199710	k__Bacteria; p__Firmicutes; c__Clostridia; o__Clostridiales; f__; g__; s__	2.198226399	1.77E-05
1133172	k__Bacteria; p__Firmicutes; c__Clostridia; o__Clostridiales; f__Peptostreptococcaceae; g__; s__	-2.616637439	1.81E-05
184209	k__Bacteria; p__Bacteroidetes; c__Bacteroidia; o__Bacteroidales; f__Bacteroidaceae; g__Bacteroides; s__	2.077319138	1.86E-05
4020502	k__Bacteria; p__Bacteroidetes; c__Bacteroidia; o__Bacteroidales; f__Bacteroidaceae; g__Bacteroides; s__	2.144705306	1.87E-05
4472130	k__Bacteria; p__Firmicutes; c__Clostridia; o__Clostridiales; f__Lachnospiraceae; g__; s__	2.365838692	1.87E-05
183650	k__Bacteria; p__Firmicutes; c__Clostridia; o__Clostridiales; f__Ruminococcaceae; g__; s__	2.616323374	1.87E-05
755148	k__Bacteria; p__Firmicutes; c__Clostridia; o__Clostridiales; f__[Tissierellaceae]; g__1-68; s__	2.680861769	2.08E-05
1028632	k__Bacteria; p__Proteobacteria; c__Gammaproteobacteria; o__Enterobacteriales; f__Enterobacteriaceae; g__; s__	2.550423932	2.18E-05
4111715	k__Bacteria; p__Proteobacteria; c__Gammaproteobacteria; o__Enterobacteriales; f__Enterobacteriaceae; g__; s__	2.721772274	2.19E-05
307113	k__Bacteria; p__Firmicutes; c__Clostridia; o__Clostridiales; f__Lachnospiraceae; g__Blautia; s__	-2.334840575	2.39E-05
4397098	k__Bacteria; p__Firmicutes; c__Clostridia; o__Clostridiales; f__[Tissierellaceae]; g__Peptoniphilus; s__	2.277164506	2.42E-05
214031	k__Bacteria; p__Bacteroidetes; c__Bacteroidia; o__Bacteroidales; f__Rikenellaceae; g__; s__	2.323979758	2.57E-05
2442706	k__Bacteria; p__Firmicutes; c__Clostridia; o__Clostridiales; f__Christensenellaceae; g__; s__	2.169756754	2.57E-05
157966	k__Bacteria; p__Firmicutes; c__Clostridia; o__Clostridiales; f__Ruminococcaceae; g__; s__	2.186576118	2.70E-05
4398028	k__Bacteria; p__Firmicutes; c__Clostridia; o__Clostridiales; f__Ruminococcaceae; g__; s__	2.578902477	2.73E-05
4405146	k__Bacteria; p__Firmicutes; c__Clostridia; o__Clostridiales; f__; g__; s__	2.751323954	2.79E-05
4435400	k__Bacteria; p__Firmicutes; c__Clostridia; o__Clostridiales; f__Lachnospiraceae; g__; s__	2.030195379	2.90E-05
4426298	k__Bacteria; p__Actinobacteria; c__Actinobacteria; o__Bifidobacteriales; f__Bifidobacteriaceae; g__Bifidobacterium; s__animalis	2.733739129	2.98E-05
180606	k__Bacteria; p__Bacteroidetes; c__Bacteroidia; o__Bacteroidales; f__Bacteroidaceae; g__Bacteroides; s__ovatus	2.080625331	3.06E-05
2876801	k__Bacteria; p__Bacteroidetes; c__Bacteroidia; o__Bacteroidales; f__Bacteroidaceae; g__Bacteroides; s__uniformis	2.123198962	3.10E-05
4442899	k__Bacteria; p__Firmicutes; c__Clostridia; o__Clostridiales; f__; g__; s__	2.546874849	3.14E-05
4402645	k__Bacteria; p__Firmicutes; c__Clostridia; o__Clostridiales; f__; g__; s__	2.478572913	3.20E-05
190991	k__Bacteria; p__Firmicutes; c__Clostridia; o__Clostridiales; f__Lachnospiraceae; g__Blautia; s__	-2.631530728	3.22E-05
3600504	k__Bacteria; p__Bacteroidetes; c__Bacteroidia; o__Bacteroidales; f__Bacteroidaceae; g__Bacteroides; s__	-2.239896983	3.23E-05
4383922	k__Bacteria; p__Firmicutes; c__Clostridia; o__Clostridiales; f__Lachnospiraceae; g__; s__	1.881433696	3.23E-05
354334	k__Bacteria; p__Firmicutes; c__Clostridia; o__Clostridiales; f__Ruminococcaceae; g__; s__	2.145514926	3.26E-05
199490	k__Bacteria; p__Firmicutes; c__Clostridia; o__Clostridiales; f__Lachnospiraceae; g__; s__	2.045919541	3.26E-05
3973322	k__Bacteria; p__Firmicutes; c__Clostridia; o__Clostridiales; f__; g__; s__	2.17586392	3.55E-05
195465	k__Bacteria; p__Firmicutes; c__Clostridia; o__Clostridiales; f__Lachnospiraceae; g__; s__	1.973259353	3.61E-05
4356062	k__Bacteria; p__Firmicutes; c__Clostridia; o__Clostridiales; f__Ruminococcaceae; g__; s__	2.399128779	3.68E-05
4410166	k__Bacteria; p__Bacteroidetes; c__Bacteroidia; o__Bacteroidales; f__Prevotellaceae; g__Prevotella; s__copri	2.059016047	3.70E-05
173965	k__Bacteria; p__Actinobacteria; c__Coriobacteriia; o__Coriobacteriales; f__Coriobacteriaceae; g__Adlercreutzia; s__	2.115554274	3.71E-05
187882	k__Bacteria; p__Firmicutes; c__Clostridia; o__Clostridiales; f__Ruminococcaceae; g__; s__	-2.805786333	3.75E-05
797229	k__Bacteria; p__Proteobacteria; c__Gammaproteobacteria; o__Enterobacteriales; f__Enterobacteriaceae; g__; s__	2.488038623	3.75E-05
191476	k__Bacteria; p__Firmicutes; c__Clostridia; o__Clostridiales; f__Lachnospiraceae; g__[Ruminococcus]; s__gnavus	-2.867593382	3.76E-05
187123	k__Bacteria; p__Firmicutes; c__Clostridia; o__Clostridiales; f__Ruminococcaceae; g__; s__	2.15973789	3.78E-05
341322	k__Bacteria; p__Firmicutes; c__Bacilli; o__Turicibacterales; f__Turicibacteraceae; g__Turicibacter; s__	-2.507491068	3.97E-05

4380450	k__Bacteria; p__Firmicutes; c__Clostridia; o__Clostridiales; f__Ruminococcaceae; g__; s__	2.229532189	4.04E-05
305224	k__Bacteria; p__Firmicutes; c__Clostridia; o__Clostridiales; f__Lachnospiraceae; g__; s__	2.532073683	4.20E-05
522433	k__Bacteria; p__Firmicutes; c__Clostridia; o__Clostridiales; f__; g__; s__	2.38514838	4.32E-05
194654	k__Bacteria; p__Firmicutes; c__Clostridia; o__Clostridiales; f__Ruminococcaceae; g__; s__	1.899723031	4.59E-05
189679	k__Bacteria; p__Firmicutes; c__Clostridia; o__Clostridiales; f__Lachnospiraceae; g__; s__	2.007426117	4.59E-05
180563	k__Bacteria; p__Firmicutes; c__Clostridia; o__Clostridiales; f__Ruminococcaceae; g__; s__	-2.342360093	4.74E-05
176008	k__Bacteria; p__Firmicutes; c__Clostridia; o__Clostridiales; f__Lachnospiraceae; g__Blautia; s__	2.085665499	4.74E-05
4434579	k__Bacteria; p__Bacteroidetes; c__Bacteroidia; o__Bacteroidales; f__Prevotellaceae; g__Prevotella; s__	2.593615908	4.74E-05
825808	k__Bacteria; p__Actinobacteria; c__Actinobacteria; o__Bifidobacteriales; f__Bifidobacteriaceae; g__Bifidobacterium; s__	2.261498346	4.75E-05
173851	k__Bacteria; p__Firmicutes; c__Clostridia; o__Clostridiales; f__Lachnospiraceae; g__; s__	2.440462438	5.11E-05
1835779	k__Bacteria; p__Firmicutes; c__Clostridia; o__Clostridiales; f__Lachnospiraceae; g__; s__	1.97454629	5.20E-05
186981	k__Bacteria; p__Bacteroidetes; c__Bacteroidia; o__Bacteroidales; f__[Barnesiellaceae]; g__; s__	2.360043908	5.53E-05
4387453	k__Bacteria; p__Firmicutes; c__Clostridia; o__Clostridiales; f__Clostridiaceae; g__; s__	2.187449248	5.54E-05
1102370	k__Bacteria; p__Firmicutes; c__Clostridia; o__Clostridiales; f__Ruminococcaceae; g__; s__	2.388604761	5.63E-05
229919	k__Bacteria; p__Firmicutes; c__Clostridia; o__Clostridiales; f__; g__; s__	2.472637171	5.83E-05
4438116	k__Bacteria; p__Bacteroidetes; c__Bacteroidia; o__Bacteroidales; f__Bacteroidaceae; g__Bacteroides; s__	2.007947577	6.28E-05
188348	k__Bacteria; p__Firmicutes; c__Clostridia; o__Clostridiales; f__Ruminococcaceae; g__; s__	2.423441142	6.35E-05
176113	k__Bacteria; p__Firmicutes; c__Clostridia; o__Clostridiales; f__; g__; s__	2.003150129	6.56E-05
147702	k__Bacteria; p__Firmicutes; c__Clostridia; o__Clostridiales; f__Ruminococcaceae; g__Faecalibacterium; s__prausnitzii	-2.422468476	6.94E-05
2056702	k__Bacteria; p__Firmicutes; c__Clostridia; o__Clostridiales; f__Ruminococcaceae; g__; s__	2.407922781	6.98E-05
179760	k__Bacteria; p__Firmicutes; c__Clostridia; o__Clostridiales; f__; g__; s__	2.065898889	7.01E-05
188764	k__Bacteria; p__Firmicutes; c__Clostridia; o__Clostridiales; f__Ruminococcaceae; g__; s__	2.298022142	7.03E-05
348304	k__Bacteria; p__Bacteroidetes; c__Bacteroidia; o__Bacteroidales; f__Bacteroidaceae; g__Bacteroides; s__	2.089723629	7.07E-05
174695	k__Bacteria; p__Firmicutes; c__Clostridia; o__Clostridiales; f__Lachnospiraceae; g__; s__	1.909550921	7.19E-05
188047	k__Bacteria; p__Firmicutes; c__Clostridia; o__Clostridiales; f__Lachnospiraceae; g__Coprococcus; s__	-2.120082153	7.22E-05
184534	k__Bacteria; p__Firmicutes; c__Clostridia; o__Clostridiales; f__Christensenellaceae; g__; s__	2.503073617	7.52E-05
4435655	k__Bacteria; p__Proteobacteria; c__Alphaproteobacteria; o__RF32; f__; g__; s__	2.321312363	7.52E-05
4479397	k__Bacteria; p__Bacteroidetes; c__Bacteroidia; o__Bacteroidales; f__Bacteroidaceae; g__Bacteroides; s__fragilis	2.412902359	7.89E-05
183824	k__Bacteria; p__Firmicutes; c__Clostridia; o__Clostridiales; f__Lachnospiraceae; g__Blautia; s__	1.902884766	8.03E-05
179729	k__Bacteria; p__Firmicutes; c__Clostridia; o__Clostridiales; f__Lachnospiraceae; g__Blautia; s__	-2.284622993	8.05E-05
4459634	k__Bacteria; p__Firmicutes; c__Clostridia; o__Clostridiales; f__Clostridiaceae; g__Clostridium; s__	-1.696200275	8.05E-05
148279	k__Bacteria; p__Firmicutes; c__Clostridia; o__Clostridiales; f__Lachnospiraceae; g__Blautia; s__	1.864363086	8.05E-05
190649	k__Bacteria; p__Firmicutes; c__Clostridia; o__Clostridiales; f__Ruminococcaceae; g__; s__	2.047641487	8.05E-05
3805726	k__Bacteria; p__Firmicutes; c__Clostridia; o__Clostridiales; f__[Mogibacteriaceae]; g__; s__	2.148328322	8.05E-05
2388617	k__Bacteria; p__Bacteroidetes; c__Bacteroidia; o__Bacteroidales; f__Bacteroidaceae; g__Bacteroides; s__	2.440718642	8.05E-05
4453501	k__Bacteria; p__Firmicutes; c__Clostridia; o__Clostridiales; f__Veillonellaceae; g__Veillonella; s__dispar	-1.978733166	8.20E-05
190100	k__Bacteria; p__Firmicutes; c__Clostridia; o__Clostridiales; f__Lachnospiraceae; g__Blautia; s__	-2.211543072	8.39E-05
190162	k__Bacteria; p__Firmicutes; c__Clostridia; o__Clostridiales; f__Lachnospiraceae; g__Blautia; s__	-2.243182477	8.40E-05
178845	k__Bacteria; p__Firmicutes; c__Clostridia; o__Clostridiales; f__Ruminococcaceae; g__; s__	2.389252865	8.52E-05
362363	k__Bacteria; p__Firmicutes; c__Clostridia; o__Clostridiales; f__Ruminococcaceae; g__Faecalibacterium; s__prausnitzii	1.920442857	8.63E-05
1846390	k__Bacteria; p__Firmicutes; c__Clostridia; o__Clostridiales; f__Clostridiaceae; g__Clostridium; s__	-2.189347463	8.99E-05
358030	k__Bacteria; p__Firmicutes; c__Clostridia; o__Clostridiales; f__Ruminococcaceae; g__; s__	2.398491566	8.99E-05
4354582	k__Bacteria; p__Firmicutes; c__Clostridia; o__Clostridiales; f__Lachnospiraceae; g__Dorea; s__	1.790116815	9.47E-05
188863	k__Bacteria; p__Firmicutes; c__Clostridia; o__Clostridiales; f__Ruminococcaceae; g__; s__	1.838160314	9.69E-05
4349261	k__Bacteria; p__Firmicutes; c__Clostridia; o__Clostridiales; f__Lachnospiraceae; g__; s__	2.361030711	9.70E-05
2035344	k__Bacteria; p__Firmicutes; c__Clostridia; o__Clostridiales; f__Lachnospiraceae; g__Blautia; s__	1.765358242	9.70E-05
185575	k__Bacteria; p__Firmicutes; c__Clostridia; o__Clostridiales; f__Ruminococcaceae; g__; s__	-2.117057185	9.88E-05

3232988	k__Bacteria; p__Tenericutes; c__RF3; o__ML615J-28; f__; g__; s__	2.460039131	9.94E-05
174763	k__Bacteria; p__Firmicutes; c__Clostridia; o__Clostridiales; f__Lachnospiraceae; g__Blautia; s__	-2.256938853	0.000104665
180552	k__Bacteria; p__Firmicutes; c__Clostridia; o__Clostridiales; f__Clostridiaceae; g__; s__	1.931125458	0.000118618
4356307	k__Bacteria; p__Firmicutes; c__Clostridia; o__Clostridiales; f__Ruminococcaceae; g__Oscillospira; s__	1.873283677	0.000129192
4370941	k__Bacteria; p__Firmicutes; c__Clostridia; o__Clostridiales; f__Ruminococcaceae; g__; s__	1.99537494	0.000135926
495017	k__Bacteria; p__Bacteroidetes; c__Bacteroidia; o__Bacteroidales; f__Porphyromonadaceae; g__Porphyromonas; s__	2.112861341	0.00013858
184339	k__Bacteria; p__Firmicutes; c__Clostridia; o__Clostridiales; f__Ruminococcaceae; g__; s__	1.8093862	0.000141868
192720	k__Bacteria; p__Firmicutes; c__Clostridia; o__Clostridiales; f__Ruminococcaceae; g__; s__	2.264382016	0.000142999
4418496	k__Bacteria; p__Bacteroidetes; c__Bacteroidia; o__Bacteroidales; f__Porphyromonadaceae; g__Parabacteroides; s__	2.011038939	0.000147174
4401375	k__Bacteria; p__Bacteroidetes; c__Bacteroidia; o__Bacteroidales; f__Bacteroidaceae; g__Bacteroides; s__uniformis	1.933847807	0.000148952
185731	k__Bacteria; p__Firmicutes; c__Clostridia; o__Clostridiales; f__Lachnospiraceae; g__Blautia; s__	-2.138436112	0.000149361
183207	k__Bacteria; p__Firmicutes; c__Clostridia; o__Clostridiales; f__Ruminococcaceae; g__; s__	1.875766569	0.000149361
571642	k__Bacteria; p__Firmicutes; c__Clostridia; o__Clostridiales; f__Ruminococcaceae; g__; s__	2.143563476	0.000149361
180721	k__Bacteria; p__Firmicutes; c__Clostridia; o__Clostridiales; f__Ruminococcaceae; g__; s__	1.871204999	0.000150968
178773	k__Bacteria; p__Firmicutes; c__Clostridia; o__Clostridiales; f__Ruminococcaceae; g__; s__	2.164628839	0.000152044
174924	k__Bacteria; p__Firmicutes; c__Clostridia; o__Clostridiales; f__Ruminococcaceae; g__Ruminococcus; s__	2.102174722	0.000154927
1104963	k__Bacteria; p__Firmicutes; c__Clostridia; o__Clostridiales; f__Clostridiaceae; g__; s__	-2.26394976	0.000154986
4331723	k__Bacteria; p__Firmicutes; c__Clostridia; o__Clostridiales; f__Ruminococcaceae; g__Ruminococcus; s__	1.911142204	0.000166068
4380971	k__Bacteria; p__Firmicutes; c__Clostridia; o__Clostridiales; f__Clostridiaceae; g__Clostridium; s__	-1.778857732	0.000167412
4439603	k__Bacteria; p__Firmicutes; c__Bacilli; o__Lactobacillales; f__Streptococcaceae; g__Streptococcus; s__	1.986603269	0.000168894
174752	k__Bacteria; p__Firmicutes; c__Clostridia; o__Clostridiales; f__Ruminococcaceae; g__; s__	-2.074637626	0.000169706
309391	k__Bacteria; p__Firmicutes; c__Clostridia; o__Clostridiales; f__; g__; s__	-2.106070806	0.000178314
192210	k__Bacteria; p__Firmicutes; c__Clostridia; o__Clostridiales; f__; g__; s__	1.720143405	0.000178314
194733	k__Bacteria; p__Firmicutes; c__Clostridia; o__Clostridiales; f__Lachnospiraceae; g__; s__	1.698507078	0.000178581
187178	k__Bacteria; p__Bacteroidetes; c__Bacteroidia; o__Bacteroidales; f__Bacteroidaceae; g__Bacteroides; s__	2.113927377	0.000178581
193551	k__Bacteria; p__Firmicutes; c__Clostridia; o__Clostridiales; f__Lachnospiraceae; g__Coprococcus; s__	1.814102022	0.000185061
324163	k__Bacteria; p__Firmicutes; c__Clostridia; o__Clostridiales; f__Lachnospiraceae; g__Blautia; s__	-1.959651833	0.000196279
4460021	k__Bacteria; p__Firmicutes; c__Clostridia; o__Clostridiales; f__Ruminococcaceae; g__Ruminococcus; s__	2.013359366	0.000196634
2331530	k__Bacteria; p__Bacteroidetes; c__Bacteroidia; o__Bacteroidales; f__Bacteroidaceae; g__Bacteroides; s__	1.837180139	0.000200679
4373156	k__Bacteria; p__Firmicutes; c__Clostridia; o__Clostridiales; f__Lachnospiraceae; g__; s__	1.824444609	0.000201711
3531225	k__Bacteria; p__Proteobacteria; c__Gammaproteobacteria; o__Enterobacteriales; f__Enterobacteriaceae; g__; s__	2.106825753	0.000205632
193148	k__Bacteria; p__Firmicutes; c__Clostridia; o__Clostridiales; f__; g__; s__	-2.102149338	0.000211328
2704013	k__Bacteria; p__Firmicutes; c__Clostridia; o__Clostridiales; f__Veillonellaceae; g__Dialister; s__	2.066579139	0.000215936
232900	k__Bacteria; p__Actinobacteria; c__Coriobacteriia; o__Coriobacteriales; f__Coriobacteriaceae; g__; s__	1.967114724	0.000226715
176381	k__Bacteria; p__Firmicutes; c__Clostridia; o__Clostridiales; f__Lachnospiraceae; g__Blautia; s__	-2.39579525	0.000232883
168289	k__Bacteria; p__Firmicutes; c__Clostridia; o__Clostridiales; f__; g__; s__	2.065875777	0.000233583
188707	k__Bacteria; p__Firmicutes; c__Clostridia; o__Clostridiales; f__Ruminococcaceae; g__; s__	1.766917021	0.00023626
185864	k__Bacteria; p__Firmicutes; c__Clostridia; o__Clostridiales; f__Lachnospiraceae; g__; s__	1.766194019	0.000236698
178064	k__Bacteria; p__Bacteroidetes; c__Bacteroidia; o__Bacteroidales; f__Bacteroidaceae; g__Bacteroides; s__	2.174298742	0.000243256
4361189	k__Bacteria; p__Firmicutes; c__Clostridia; o__Clostridiales; f__Lachnospiraceae; g__Blautia; s__	1.993506863	0.00024441
185583	k__Bacteria; p__Firmicutes; c__Clostridia; o__Clostridiales; f__Ruminococcaceae; g__; s__	-2.024905245	0.000251541
4462599	k__Bacteria; p__Firmicutes; c__Clostridia; o__Clostridiales; f__; g__; s__	1.875197546	0.000251541
308760	k__Bacteria; p__Tenericutes; c__Mollicutes; o__RF39; f__; g__; s__	2.184903229	0.000253346
184394	k__Bacteria; p__Firmicutes; c__Clostridia; o__Clostridiales; f__Lachnospiraceae; g__; s__	1.939767538	0.000253982
177515	k__Bacteria; p__Firmicutes; c__Clostridia; o__Clostridiales; f__Lachnospiraceae; g__Roseburia; s__	-2.073333084	0.000265703
4468506	k__Bacteria; p__Firmicutes; c__Clostridia; o__Clostridiales; f__Lachnospiraceae; g__Blautia; s__producta	2.006318448	0.000266787
179905	k__Bacteria; p__Firmicutes; c__Clostridia; o__Clostridiales; f__Lachnospiraceae; g__; s__	1.702068672	0.000268122

14205	k__Bacteria; p__Firmicutes; c__Clostridia; o__Clostridiales; f__Peptostreptococcaceae; g__[Clostridium]; s__sordellii	-2.073367315	0.000268574
849440	k__Archaea; p__Euryarchaeota; c__Methanobacteria; o__Methanobacteriales; f__Methanobacteriaceae; g__Methanobrevibacter; s__	2.17059364	0.000269766
4396298	k__Bacteria; p__Firmicutes; c__Clostridia; o__Clostridiales; f__Ruminococcaceae; g__; s__	2.118269793	0.000282314
187952	k__Bacteria; p__Firmicutes; c__Clostridia; o__Clostridiales; f__Lachnospiraceae; g__Blautia; s__	-2.072688232	0.000287594
317315	k__Bacteria; p__Firmicutes; c__Clostridia; o__Clostridiales; f__; g__; s__	2.321580689	0.000288633
175560	k__Bacteria; p__Firmicutes; c__Clostridia; o__Clostridiales; f__Ruminococcaceae; g__Faecalibacterium; s__prausnitzii	1.730326146	0.000292245
4396688	k__Bacteria; p__Firmicutes; c__Clostridia; o__Clostridiales; f__Lachnospiraceae; g__[Ruminococcus]; s__	2.06534339	0.000292433
177349	k__Bacteria; p__Firmicutes; c__Clostridia; o__Clostridiales; f__Lachnospiraceae; g__Blautia; s__	-2.095277266	0.000295563
199145	k__Bacteria; p__Firmicutes; c__Clostridia; o__Clostridiales; f__Ruminococcaceae; g__Faecalibacterium; s__prausnitzii	-1.927436242	0.000296164
177342	k__Bacteria; p__Firmicutes; c__Clostridia; o__Clostridiales; f__Ruminococcaceae; g__; s__	1.868353021	0.000305584
7366	k__Bacteria; p__Proteobacteria; c__Betaproteobacteria; o__Burkholderiales; f__Oxalobacteraceae; g__Oxalobacter; s__formigenes	1.907262312	0.000306937
4431558	k__Bacteria; p__Firmicutes; c__Clostridia; o__Clostridiales; f__Lachnospiraceae; g__; s__	1.873565523	0.000308544
360636	k__Bacteria; p__Firmicutes; c__Erysipelotrichi; o__Erysipelotrichales; f__Erysipelotrichaceae; g__; s__	-1.960496027	0.000311453
4479989	k__Bacteria; p__Firmicutes; c__Bacilli; o__Lactobacillales; f__Streptococcaceae; g__Streptococcus; s__	1.814322266	0.000311453
3422630	k__Bacteria; p__Firmicutes; c__Clostridia; o__Clostridiales; f__Ruminococcaceae; g__; s__	1.874074054	0.000311453
339566	k__Bacteria; p__Firmicutes; c__Clostridia; o__Clostridiales; f__Ruminococcaceae; g__; s__	1.88983722	0.000311453
191361	k__Bacteria; p__Firmicutes; c__Clostridia; o__Clostridiales; f__Lachnospiraceae; g__; s__	-1.886788477	0.000315335
4465124	k__Bacteria; p__Firmicutes; c__Clostridia; o__Clostridiales; f__Clostridiaceae; g__Clostridium; s__	2.082078418	0.000338639
182577	k__Bacteria; p__Firmicutes; c__Clostridia; o__Clostridiales; f__Ruminococcaceae; g__; s__	-2.348773972	0.000339017
187929	k__Bacteria; p__Firmicutes; c__Clostridia; o__Clostridiales; f__; g__; s__	1.770118388	0.000339017
176104	k__Bacteria; p__Firmicutes; c__Clostridia; o__Clostridiales; f__Ruminococcaceae; g__Oscillospira; s__	1.927012617	0.000339017
187248	k__Bacteria; p__Firmicutes; c__Clostridia; o__Clostridiales; f__Lachnospiraceae; g__; s__	2.069160763	0.000339017
177040	k__Bacteria; p__Firmicutes; c__Clostridia; o__Clostridiales; f__Lachnospiraceae; g__; s__	-1.939126455	0.000339807
188329	k__Bacteria; p__Firmicutes; c__Clostridia; o__Clostridiales; f__Ruminococcaceae; g__Faecalibacterium; s__prausnitzii	1.691705917	0.000341755
1100471	k__Bacteria; p__Firmicutes; c__Clostridia; o__Clostridiales; f__[Tissierellaceae]; g__Peptoniphilus; s__	1.962843003	0.00034352
196200	k__Bacteria; p__Firmicutes; c__Clostridia; o__Clostridiales; f__Lachnospiraceae; g__Blautia; s__	-2.039030413	0.000347387
193367	k__Bacteria; p__Firmicutes; c__Clostridia; o__Clostridiales; f__Lachnospiraceae; g__Blautia; s__	-2.0299401	0.000348987
4052330	k__Bacteria; p__Firmicutes; c__Clostridia; o__Clostridiales; f__Lachnospiraceae; g__; s__	1.980662535	0.000352005
731422	k__Bacteria; p__Firmicutes; c__Clostridia; o__Clostridiales; f__Veillonellaceae; g__Phascolarctobacterium; s__	2.152604095	0.000353984
176244	k__Bacteria; p__Firmicutes; c__Clostridia; o__Clostridiales; f__Lachnospiraceae; g__Blautia; s__	-2.11187254	0.000371022
187404	k__Bacteria; p__Firmicutes; c__Clostridia; o__Clostridiales; f__Ruminococcaceae; g__; s__	1.85260652	0.000371022
4476527	k__Bacteria; p__Firmicutes; c__Clostridia; o__Clostridiales; f__Ruminococcaceae; g__Faecalibacterium; s__prausnitzii	1.664061249	0.00038201
839964	k__Bacteria; p__Firmicutes; c__Clostridia; o__Clostridiales; f__Ruminococcaceae; g__Oscillospira; s__	1.855376815	0.000384186
4410097	k__Bacteria; p__Firmicutes; c__Clostridia; o__Clostridiales; f__Christensenellaceae; g__; s__	2.178154373	0.000386031
180352	k__Bacteria; p__Firmicutes; c__Clostridia; o__Clostridiales; f__; g__; s__	1.575153993	0.000398255
166869	k__Bacteria; p__Firmicutes; c__Clostridia; o__Clostridiales; f__; g__; s__	1.754867633	0.000401838
1945397	k__Bacteria; p__Firmicutes; c__Clostridia; o__Clostridiales; f__; g__; s__	2.110988533	0.000406304
176062	k__Bacteria; p__Firmicutes; c__Clostridia; o__Clostridiales; f__; g__; s__	2.060231876	0.000408022
321453	k__Bacteria; p__Firmicutes; c__Clostridia; o__Clostridiales; f__Lachnospiraceae; g__Blautia; s__	-2.062574128	0.000421719
1602805	k__Bacteria; p__Firmicutes; c__Clostridia; o__Clostridiales; f__Lachnospiraceae; g__; s__	1.766132637	0.000421719
183210	k__Bacteria; p__Firmicutes; c__Clostridia; o__Clostridiales; f__Lachnospiraceae; g__Blautia; s__	1.824876307	0.000423662
2840201	k__Bacteria; p__Firmicutes; c__Clostridia; o__Clostridiales; f__Clostridiaceae; g__Clostridium; s__	-2.152787053	0.00043387
4470870	k__Bacteria; p__Bacteroidetes; c__Bacteroidia; o__Bacteroidales; f__[Barnesiellaceae]; g__; s__	2.028946224	0.00043825
173135	k__Bacteria; p__Firmicutes; c__Clostridia; o__Clostridiales; f__Ruminococcaceae; g__Faecalibacterium; s__prausnitzii	1.672748732	0.000440104
180421	k__Bacteria; p__Bacteroidetes; c__Bacteroidia; o__Bacteroidales; f__Bacteroidaceae; g__Bacteroides; s__	1.791481371	0.000441379
345951	k__Bacteria; p__Proteobacteria; c__Gammaproteobacteria; o__Enterobacteriales; f__Enterobacteriaceae; g__; s__	1.898151127	0.000461602
516553	k__Bacteria; p__Firmicutes; c__Clostridia; o__Clostridiales; f__Lachnospiraceae; g__; s__	1.8857356	0.000476547

1600780	k__Bacteria; p__Firmicutes; c__Clostridia; o__Clostridiales; f__Lachnospiraceae; g__; s__	1.780864184	0.000486095
661266	k__Bacteria; p__Firmicutes; c__Clostridia; o__Clostridiales; f__Lachnospiraceae; g__Blautia; s__	-1.884759214	0.000490732
324015	k__Bacteria; p__Firmicutes; c__Clostridia; o__Clostridiales; f__; g__; s__	1.754063366	0.000491725
198221	k__Bacteria; p__Firmicutes; c__Clostridia; o__Clostridiales; f__Ruminococcaceae; g__; s__	1.63425825	0.000493312
1790209	k__Bacteria; p__Firmicutes; c__Clostridia; o__Clostridiales; f__Ruminococcaceae; g__; s__	1.776026051	0.000495095
348027	k__Bacteria; p__Bacteroidetes; c__Bacteroidia; o__Bacteroidales; f__Bacteroidaceae; g__Bacteroides; s__uniformis	1.688196761	0.000496315
193666	k__Bacteria; p__Firmicutes; c__Clostridia; o__Clostridiales; f__Lachnospiraceae; g__Blautia; s__	-2.080853342	0.000499768
172163	k__Bacteria; p__Firmicutes; c__Clostridia; o__Clostridiales; f__; g__; s__	2.036875767	0.000510984
4336940	k__Bacteria; p__Firmicutes; c__Clostridia; o__Clostridiales; f__; g__; s__	1.898817204	0.000515365
2225203	k__Bacteria; p__Firmicutes; c__Clostridia; o__Clostridiales; f__Clostridiaceae; g__; s__	-2.142182327	0.000515452
708680	k__Bacteria; p__Firmicutes; c__Clostridia; o__Clostridiales; f__Lachnospiraceae; g__; s__	1.575476729	0.00051864
311947	k__Bacteria; p__Firmicutes; c__Clostridia; o__Clostridiales; f__Ruminococcaceae; g__; s__	1.904100928	0.000523627
994357	k__Bacteria; p__Firmicutes; c__Clostridia; o__Clostridiales; f__[Mogibacteriaceae]; g__Mogibacterium; s__	1.868874832	0.000530739
217109	k__Bacteria; p__Firmicutes; c__Clostridia; o__Clostridiales; f__Christensenellaceae; g__; s__	1.668943402	0.000543278
360890	k__Bacteria; p__Firmicutes; c__Clostridia; o__Clostridiales; f__; g__; s__	1.665763511	0.000549857
4354477	k__Bacteria; p__Proteobacteria; c__Gammaproteobacteria; o__Enterobacteriales; f__Enterobacteriaceae; g__; s__	2.001293992	0.00057243
2990918	k__Bacteria; p__Actinobacteria; c__Coriobacteriia; o__Coriobacteriales; f__Coriobacteriaceae; g__Collinsella; s__stercoris	2.010679296	0.000574157
337538	k__Bacteria; p__Firmicutes; c__Clostridia; o__Clostridiales; f__Lachnospiraceae; g__; s__	1.51119252	0.000575627
2829179	k__Bacteria; p__Firmicutes; c__Clostridia; o__Clostridiales; f__Veillonellaceae; g__Acidaminococcus; s__	2.081748947	0.000588231
573061	k__Bacteria; p__Proteobacteria; c__Deltaproteobacteria; o__Desulfovibrionales; f__Desulfovibrionaceae; g__Desulfovibrio; s__	1.903982326	0.000592541
4300690	k__Bacteria; p__Firmicutes; c__Clostridia; o__Clostridiales; f__Ruminococcaceae; g__; s__	1.893708822	0.00062585
179744	k__Bacteria; p__Firmicutes; c__Clostridia; o__Clostridiales; f__; g__; s__	1.770629313	0.000633407
194626	k__Bacteria; p__Firmicutes; c__Clostridia; o__Clostridiales; f__Ruminococcaceae; g__; s__	1.518953167	0.000641953
4436046	k__Bacteria; p__Firmicutes; c__Clostridia; o__Clostridiales; f__Lachnospiraceae; g__Dorea; s__	1.888658567	0.000643877
183873	k__Bacteria; p__Firmicutes; c__Clostridia; o__Clostridiales; f__Lachnospiraceae; g__[Ruminococcus]; s__	1.707162827	0.000672635
2190939	k__Bacteria; p__Firmicutes; c__Clostridia; o__Clostridiales; f__; g__; s__	-2.065925031	0.000673526
191442	k__Bacteria; p__Firmicutes; c__Clostridia; o__Clostridiales; f__Ruminococcaceae; g__; s__	1.590724129	0.000685385
30062	k__Bacteria; p__Firmicutes; c__Clostridia; o__Clostridiales; f__[Tissierellaceae]; g__Anaerococcus; s__	1.830588956	0.000698253
514257	k__Bacteria; p__Firmicutes; c__Clostridia; o__Clostridiales; f__; g__; s__	1.918197978	0.000716193
192438	k__Bacteria; p__Firmicutes; c__Clostridia; o__Clostridiales; f__Ruminococcaceae; g__; s__	1.593781421	0.000769739
4354235	k__Bacteria; p__Firmicutes; c__Clostridia; o__Clostridiales; f__Lachnospiraceae; g__; s__	-1.508756103	0.000776238
199694	k__Bacteria; p__Firmicutes; c__Clostridia; o__Clostridiales; f__Clostridiaceae; g__Clostridium; s__	1.582481371	0.000810717
158771	k__Bacteria; p__Firmicutes; c__Clostridia; o__Clostridiales; f__; g__; s__	1.791381162	0.000815098
193969	k__Bacteria; p__Firmicutes; c__Clostridia; o__Clostridiales; f__Lachnospiraceae; g__Coprococcus; s__	1.939096881	0.00081757
4442508	k__Bacteria; p__Firmicutes; c__Clostridia; o__Clostridiales; f__Lachnospiraceae; g__; s__	1.589038721	0.000826357
185563	k__Bacteria; p__Firmicutes; c__Clostridia; o__Clostridiales; f__Lachnospiraceae; g__Blautia; s__	-1.911242235	0.000830486
4451907	k__Bacteria; p__Firmicutes; c__Clostridia; o__Clostridiales; f__Lachnospiraceae; g__Dorea; s__	1.732284875	0.000888693
195004	k__Bacteria; p__Firmicutes; c__Clostridia; o__Clostridiales; f__; g__; s__	1.52554475	0.000900238
182033	k__Bacteria; p__Firmicutes; c__Clostridia; o__Clostridiales; f__Ruminococcaceae; g__Oscillospira; s__	1.495225441	0.000909396
137056	k__Bacteria; p__Firmicutes; c__Bacilli; o__Bacillales; f__Planococcaceae; g__; s__	-1.627524797	0.000912253
190639	k__Bacteria; p__Bacteroidetes; c__Bacteroidia; o__Bacteroidales; f__[Odoribacteraceae]; g__Butyricimonas; s__	1.664664963	0.000929948
183585	k__Bacteria; p__Firmicutes; c__Clostridia; o__Clostridiales; f__Lachnospiraceae; g__Blautia; s__	-1.749635573	0.000931508
1010876	k__Bacteria; p__Firmicutes; c__Clostridia; o__Clostridiales; f__Ruminococcaceae; g__Oscillospira; s__	-1.989264584	0.000938175
189867	k__Bacteria; p__Firmicutes; c__Clostridia; o__Clostridiales; f__Ruminococcaceae; g__Ruminococcus; s__	1.669423505	0.000939857
3327894	k__Bacteria; p__Bacteroidetes; c__Bacteroidia; o__Bacteroidales; f__Bacteroidaceae; g__Bacteroides; s__uniformis	-1.485524776	0.001021162
197581	k__Bacteria; p__Firmicutes; c__Clostridia; o__Clostridiales; f__Lachnospiraceae; g__Lachnospira; s__	1.605489176	0.001021162
4440335	k__Bacteria; p__Firmicutes; c__Clostridia; o__Clostridiales; f__Christensenellaceae; g__; s__	1.849094514	0.001029369

4315396	k__Bacteria; p__Firmicutes; c__Clostridia; o__Clostridiales; f__Peptococcaceae; g__rc4-4; s__	1.832141483	0.001038744
2506486	k__Bacteria; p__Firmicutes; c__Clostridia; o__Clostridiales; f__Ruminococcaceae; g__; s__	1.566100135	0.001065981
178478	k__Bacteria; p__Bacteroidetes; c__Bacteroidia; o__Bacteroidales; f__Bacteroidaceae; g__Bacteroides; s__	1.580590161	0.001072182
4423384	k__Bacteria; p__Firmicutes; c__Clostridia; o__Clostridiales; f__Lachnospiraceae; g__Blautia; s__obeum	1.683961718	0.001072182
350503	k__Bacteria; p__Firmicutes; c__Clostridia; o__Clostridiales; f__Ruminococcaceae; g__; s__	1.78547604	0.001072182
183454	k__Bacteria; p__Firmicutes; c__Clostridia; o__Clostridiales; f__Ruminococcaceae; g__; s__	1.727991722	0.001088603
180572	k__Bacteria; p__Firmicutes; c__Clostridia; o__Clostridiales; f__Ruminococcaceae; g__Faecalibacterium; s__prausnitzii	1.440668853	0.001106668
532203	k__Bacteria; p__Firmicutes; c__Clostridia; o__Clostridiales; f__Lachnospiraceae; g__Blautia; s__	-1.708391234	0.001114485
3134492	k__Bacteria; p__Firmicutes; c__Clostridia; o__Clostridiales; f__Lachnospiraceae; g__; s__	-1.84325062	0.001182244
4331760	k__Bacteria; p__Bacteroidetes; c__Bacteroidia; o__Bacteroidales; f__Rikenellaceae; g__Alistipes; s__indistinctus	1.505270596	0.001206098
586453	k__Bacteria; p__Firmicutes; c__Clostridia; o__Clostridiales; f__Christensenellaceae; g__; s__	1.716144608	0.001217196
1751298	k__Bacteria; p__Firmicutes; c__Clostridia; o__Clostridiales; f__Lachnospiraceae; g__Roseburia; s__	-1.746356654	0.001225871
4484075	k__Bacteria; p__Firmicutes; c__Clostridia; o__Clostridiales; f__Ruminococcaceae; g__; s__	1.445620548	0.001233967
175642	k__Bacteria; p__Firmicutes; c__Clostridia; o__Clostridiales; f__Lachnospiraceae; g__; s__	1.535965838	0.001256465
1100972	k__Bacteria; p__Firmicutes; c__Bacilli; o__Lactobacillales; f__Streptococcaceae; g__Lactococcus; s__	1.596081445	0.001267363
230421	k__Bacteria; p__Firmicutes; c__Clostridia; o__Clostridiales; f__Ruminococcaceae; g__; s__	-1.917909345	0.001300854
557974	k__Bacteria; p__Proteobacteria; c__Gammaproteobacteria; o__Pseudomonadales; f__Pseudomonadaceae; g__Pseudomonas; s__	-1.670118636	0.001346041
721569	k__Bacteria; p__Firmicutes; c__Clostridia; o__Clostridiales; f__Ruminococcaceae; g__; s__	1.834320489	0.001383538
128382	k__Bacteria; p__Firmicutes; c__Clostridia; o__Clostridiales; f__Veillonellaceae; g__Dialister; s__	1.636540103	0.001386778
177828	k__Bacteria; p__Firmicutes; c__Clostridia; o__Clostridiales; f__Lachnospiraceae; g__Anaerostipes; s__	1.709004691	0.001403057
728119	k__Bacteria; p__Proteobacteria; c__Gammaproteobacteria; o__Pseudomonadales; f__Pseudomonadaceae; g__Pseudomonas; s__	1.661535349	0.001412614
12574	k__Bacteria; p__Actinobacteria; c__Actinobacteria; o__Actinomycetales; f__Actinomycetaceae; g__Actinomyces; s__	1.653554533	0.001430026
190980	k__Bacteria; p__Firmicutes; c__Clostridia; o__Clostridiales; f__; g__; s__	-1.661245333	0.001430501
210262	k__Bacteria; p__Firmicutes; c__Clostridia; o__Clostridiales; f__; g__; s__	1.769227622	0.001433531
195892	k__Bacteria; p__Firmicutes; c__Clostridia; o__Clostridiales; f__Ruminococcaceae; g__; s__	-1.814187524	0.00150278
183533	k__Bacteria; p__Firmicutes; c__Clostridia; o__Clostridiales; f__Lachnospiraceae; g__Coprococcus; s__	1.908430709	0.00150732
179400	k__Bacteria; p__Firmicutes; c__Clostridia; o__Clostridiales; f__; g__; s__	-1.457697176	0.001513086
194670	k__Bacteria; p__Bacteroidetes; c__Bacteroidia; o__Bacteroidales; f__Bacteroidaceae; g__Bacteroides; s__	1.495568473	0.001577378
181239	k__Bacteria; p__Bacteroidetes; c__Bacteroidia; o__Bacteroidales; f__Bacteroidaceae; g__Bacteroides; s__	1.520603297	0.001577378
4458306	k__Bacteria; p__Firmicutes; c__Clostridia; o__Clostridiales; f__Veillonellaceae; g__Veillonella; s__dispar	1.521558248	0.00160433
183340	k__Bacteria; p__Firmicutes; c__Clostridia; o__Clostridiales; f__Lachnospiraceae; g__Blautia; s__	-1.720396703	0.001617048
181719	k__Bacteria; p__Bacteroidetes; c__Bacteroidia; o__Bacteroidales; f__Bacteroidaceae; g__Bacteroides; s__	1.602457941	0.001664322
186906	k__Bacteria; p__Firmicutes; c__Clostridia; o__Clostridiales; f__Lachnospiraceae; g__; s__	1.41315596	0.001678793
214036	k__Bacteria; p__Firmicutes; c__Clostridia; o__Clostridiales; f__[Mogibacteriaceae]; g__; s__	1.493356251	0.001729722
194586	k__Bacteria; p__Firmicutes; c__Clostridia; o__Clostridiales; f__Lachnospiraceae; g__Blautia; s__	-1.642984504	0.001749023
182044	k__Bacteria; p__Firmicutes; c__Clostridia; o__Clostridiales; f__Ruminococcaceae; g__; s__	1.538114062	0.001757082
4402605	k__Bacteria; p__Firmicutes; c__Clostridia; o__Clostridiales; f__Christensenellaceae; g__; s__	1.773557971	0.001801632
1885089	k__Bacteria; p__Firmicutes; c__Clostridia; o__Clostridiales; f__Ruminococcaceae; g__; s__	1.464073752	0.001844971
175844	k__Bacteria; p__Bacteroidetes; c__Bacteroidia; o__Bacteroidales; f__[Barnesiellaceae]; g__; s__	1.65184572	0.001868477
1679707	k__Bacteria; p__Firmicutes; c__Clostridia; o__Clostridiales; f__Ruminococcaceae; g__; s__	1.575978883	0.001909963
1000986	k__Bacteria; p__Actinobacteria; c__Actinobacteria; o__Actinomycetales; f__Corynebacteriaceae; g__Corynebacterium; s__	1.40454414	0.001928816
975306	k__Bacteria; p__Firmicutes; c__Clostridia; o__Clostridiales; f__Lachnospiraceae; g__; s__	1.413981362	0.001928816
4364814	k__Bacteria; p__Actinobacteria; c__Actinobacteria; o__Actinomycetales; f__Corynebacteriaceae; g__Corynebacterium; s__	1.605988909	0.001928816
2137906	k__Bacteria; p__Firmicutes; c__Clostridia; o__Clostridiales; f__Lachnospiraceae; g__Blautia; s__	1.6734379	0.001928816
363029	k__Bacteria; p__Firmicutes; c__Clostridia; o__Clostridiales; f__Lachnospiraceae; g__Blautia; s__	-1.570591179	0.00196127
4387771	k__Bacteria; p__Firmicutes; c__Clostridia; o__Clostridiales; f__Ruminococcaceae; g__; s__	1.51287106	0.002010014
3141342	k__Bacteria; p__Firmicutes; c__Clostridia; o__Clostridiales; f__Lachnospiraceae; g__Coprococcus; s__	1.433671926	0.002033777

195937	k__Bacteria; p__Firmicutes; c__Clostridia; o__Clostridiales; f__Lachnospiraceae; g__Blautia; s__	-1.825566258	0.002075516
185516	k__Bacteria; p__Firmicutes; c__Clostridia; o__Clostridiales; f__Lachnospiraceae; g__; s__	1.368187781	0.002075516
849346	k__Bacteria; p__Firmicutes; c__Clostridia; o__Clostridiales; f__Ruminococcaceae; g__; s__	1.595807304	0.002082635
229348	k__Bacteria; p__Firmicutes; c__Clostridia; o__Clostridiales; f__; g__; s__	1.559786001	0.00208706
3673770	k__Bacteria; p__Firmicutes; c__Clostridia; o__Clostridiales; f__Lachnospiraceae; g__Dorea; s__	1.677504951	0.002114462
4318470	k__Bacteria; p__Bacteroidetes; c__Bacteroidia; o__Bacteroidales; f__Bacteroidaceae; g__Bacteroides; s__	1.479507032	0.002180197
342380	k__Bacteria; p__Firmicutes; c__Clostridia; o__Clostridiales; f__Lachnospiraceae; g__Blautia; s__	-1.504133483	0.002181319
289293	k__Bacteria; p__Firmicutes; c__Clostridia; o__Clostridiales; f__Clostridiaceae; g__Clostridium; s__perfringens	-1.55555052	0.002182758
1023075	k__Bacteria; p__Firmicutes; c__Clostridia; o__Clostridiales; f__Veillonellaceae; g__Veillonella; s__	-1.480006643	0.002195243
315982	k__Bacteria; p__Firmicutes; c__Clostridia; o__Clostridiales; f__Clostridiaceae; g__Clostridium; s__perfringens	-1.750009367	0.002211505
328544	k__Bacteria; p__Firmicutes; c__Clostridia; o__Clostridiales; f__Ruminococcaceae; g__; s__	-1.825337774	0.00222241
191153	k__Bacteria; p__Firmicutes; c__Clostridia; o__Clostridiales; f__Ruminococcaceae; g__; s__	-1.483242411	0.002222823
14280	k__Bacteria; p__Firmicutes; c__Clostridia; o__Clostridiales; f__[Tissierellaceae]; g__Peptoniphilus; s__	1.562087132	0.00222634
4350035	k__Bacteria; p__Firmicutes; c__Clostridia; o__Clostridiales; f__Lachnospiraceae; g__; s__	1.343783576	0.002242345
4004998	k__Bacteria; p__Firmicutes; c__Clostridia; o__Clostridiales; f__Lachnospiraceae; g__; s__	1.621414817	0.002283216
334459	k__Bacteria; p__Proteobacteria; c__Betaproteobacteria; o__Burkholderiales; f__; g__; s__	1.558898068	0.002293968
2656868	k__Bacteria; p__Bacteroidetes; c__Bacteroidia; o__Bacteroidales; f__Bacteroidaceae; g__Bacteroides; s__	1.414424113	0.002369502
192424	k__Bacteria; p__Firmicutes; c__Clostridia; o__Clostridiales; f__Lachnospiraceae; g__Blautia; s__	-1.63665306	0.002371155
4425495	k__Bacteria; p__Bacteroidetes; c__Bacteroidia; o__Bacteroidales; f__Bacteroidaceae; g__Bacteroides; s__	1.61495798	0.002371603
4439530	k__Bacteria; p__Firmicutes; c__Clostridia; o__Clostridiales; f__Clostridiaceae; g__; s__	-1.528827408	0.002416865
296872	k__Bacteria; p__Firmicutes; c__Clostridia; o__Clostridiales; f__Lachnospiraceae; g__; s__	-1.35259123	0.002542812
355471	k__Bacteria; p__Firmicutes; c__Clostridia; o__Clostridiales; f__Clostridiaceae; g__; s__	-1.75011981	0.00254609
182331	k__Bacteria; p__Firmicutes; c__Clostridia; o__Clostridiales; f__Lachnospiraceae; g__; s__	1.451333894	0.002571635
335577	k__Bacteria; p__Firmicutes; c__Clostridia; o__Clostridiales; f__Lachnospiraceae; g__Blautia; s__	-1.71240663	0.002578567
191978	k__Bacteria; p__Firmicutes; c__Clostridia; o__Clostridiales; f__Lachnospiraceae; g__Blautia; s__	1.343077639	0.002578567
194443	k__Bacteria; p__Firmicutes; c__Clostridia; o__Clostridiales; f__Ruminococcaceae; g__; s__	-1.673777182	0.00257929
232030	k__Bacteria; p__Firmicutes; c__Clostridia; o__Clostridiales; f__Lachnospiraceae; g__Coprococcus; s__	1.558704274	0.00257929
328617	k__Bacteria; p__Bacteroidetes; c__Bacteroidia; o__Bacteroidales; f__Bacteroidaceae; g__Bacteroides; s__uniformis	-1.550606031	0.002607039
190490	k__Bacteria; p__Firmicutes; c__Clostridia; o__Clostridiales; f__Ruminococcaceae; g__; s__	1.568117275	0.002625768
1105984	k__Bacteria; p__Bacteroidetes; c__Bacteroidia; o__Bacteroidales; f__Bacteroidaceae; g__Bacteroides; s__	1.495579174	0.002679307
4370657	k__Bacteria; p__Firmicutes; c__Clostridia; o__Clostridiales; f__Clostridiaceae; g__Clostridium; s__perfringens	-1.687884439	0.002696271
183662	k__Bacteria; p__Bacteroidetes; c__Bacteroidia; o__Bacteroidales; f__Bacteroidaceae; g__Bacteroides; s__	1.392196526	0.002700689
193534	k__Bacteria; p__Bacteroidetes; c__Bacteroidia; o__Bacteroidales; f__Bacteroidaceae; g__Bacteroides; s__	1.585451676	0.002741667
291266	k__Bacteria; p__Firmicutes; c__Clostridia; o__Clostridiales; f__Ruminococcaceae; g__; s__	1.452315955	0.00278976
195619	k__Bacteria; p__Firmicutes; c__Clostridia; o__Clostridiales; f__Ruminococcaceae; g__; s__	1.341739115	0.002800133
197179	k__Bacteria; p__Firmicutes; c__Clostridia; o__Clostridiales; f__Ruminococcaceae; g__; s__	1.507022971	0.002816764
187385	k__Bacteria; p__Firmicutes; c__Clostridia; o__Clostridiales; f__Lachnospiraceae; g__Blautia; s__	-1.622423846	0.00292359
691423	k__Bacteria; p__Proteobacteria; c__Gammaproteobacteria; o__Enterobacteriales; f__Enterobacteriaceae; g__; s__	1.459495165	0.002947437
4451152	k__Bacteria; p__Bacteroidetes; c__Bacteroidia; o__Bacteroidales; f__Bacteroidaceae; g__Bacteroides; s__	1.610010696	0.002959622
503372	k__Bacteria; p__Firmicutes; c__Clostridia; o__Clostridiales; f__[Tissierellaceae]; g__Anaerococcus; s__	1.52814282	0.002972293
146586	k__Bacteria; p__Firmicutes; c__Clostridia; o__Clostridiales; f__Ruminococcaceae; g__Oscillospira; s__	1.585512698	0.00299042
196518	k__Bacteria; p__Firmicutes; c__Clostridia; o__Clostridiales; f__Ruminococcaceae; g__; s__	-1.551789101	0.003140333
180462	k__Bacteria; p__Firmicutes; c__Clostridia; o__Clostridiales; f__Ruminococcaceae; g__; s__	-1.689072735	0.003147135
566243	k__Bacteria; p__Proteobacteria; c__Gammaproteobacteria; o__Enterobacteriales; f__Enterobacteriaceae; g__; s__	1.564053374	0.003155549
529940	k__Bacteria; p__Firmicutes; c__Clostridia; o__Clostridiales; f__Ruminococcaceae; g__Faecalibacterium; s__prausnitzii	1.33702995	0.003180594
185088	k__Bacteria; p__Firmicutes; c__Clostridia; o__Clostridiales; f__Lachnospiraceae; g__[Ruminococcus]; s__gnavus	1.386455886	0.003180594
184465	k__Bacteria; p__Firmicutes; c__Clostridia; o__Clostridiales; f__Lachnospiraceae; g__; s__	1.314257682	0.003193735

227819	k__Bacteria; p__Actinobacteria; c__Coriobacteriia; o__Coriobacteriales; f__Coriobacteriaceae; g__; s__	1.470661139	0.003234988
191779	k__Bacteria; p__Firmicutes; c__Clostridia; o__Clostridiales; f__Lachnospiraceae; g__Blautia; s__	-1.476999645	0.003303942
4327141	k__Bacteria; p__Firmicutes; c__Clostridia; o__Clostridiales; f__Lachnospiraceae; g__Blautia; s__	1.297153041	0.003310824
389371	k__Bacteria; p__Firmicutes; c__Clostridia; o__Clostridiales; f__; g__; s__	1.369609306	0.003310824
1943669	k__Bacteria; p__Firmicutes; c__Clostridia; o__Clostridiales; f__Ruminococcaceae; g__Faecalibacterium; s__prausnitzii	1.322516311	0.003370577
189708	k__Bacteria; p__Firmicutes; c__Clostridia; o__Clostridiales; f__Ruminococcaceae; g__Faecalibacterium; s__prausnitzii	1.268094411	0.003384865
19611	k__Bacteria; p__Firmicutes; c__Clostridia; o__Clostridiales; f__Ruminococcaceae; g__; s__	1.490072029	0.003384865
2237211	k__Bacteria; p__Bacteroidetes; c__Bacteroidia; o__Bacteroidales; f__Bacteroidaceae; g__Bacteroides; s__	1.294702552	0.003429406
197490	k__Bacteria; p__Bacteroidetes; c__Bacteroidia; o__Bacteroidales; f__Bacteroidaceae; g__Bacteroides; s__uniformis	1.404751855	0.003429406
4377149	k__Bacteria; p__Firmicutes; c__Clostridia; o__Clostridiales; f__; g__; s__	1.5384634	0.003432959
4442459	k__Bacteria; p__Bacteroidetes; c__Bacteroidia; o__Bacteroidales; f__Porphyromonadaceae; g__Parabacteroides; s__	1.377086002	0.00345899
4323124	k__Bacteria; p__Bacteroidetes; c__Bacteroidia; o__Bacteroidales; f__[Barnesiellaceae]; g__; s__	1.478081262	0.003511367
304973	k__Bacteria; p__Firmicutes; c__Clostridia; o__Clostridiales; f__Ruminococcaceae; g__Oscillospira; s__	1.404127654	0.003547267
3430935	k__Bacteria; p__Firmicutes; c__Clostridia; o__Clostridiales; f__Ruminococcaceae; g__Faecalibacterium; s__prausnitzii	1.266622657	0.003557074
197364	k__Bacteria; p__Firmicutes; c__Clostridia; o__Clostridiales; f__; g__; s__	-1.454533548	0.003583357
2749126	k__Bacteria; p__Proteobacteria; c__Gammaproteobacteria; o__Enterobacteriales; f__Enterobacteriaceae; g__; s__	1.493505959	0.003616071
179159	k__Bacteria; p__Firmicutes; c__Clostridia; o__Clostridiales; f__; g__; s__	1.612855915	0.003625517
181827	k__Bacteria; p__Firmicutes; c__Clostridia; o__Clostridiales; f__Ruminococcaceae; g__; s__	1.402077718	0.003719374
4427459	k__Bacteria; p__Firmicutes; c__Clostridia; o__Clostridiales; f__Ruminococcaceae; g__Ruminococcus; s__	1.513475038	0.003732268
175509	k__Bacteria; p__Firmicutes; c__Clostridia; o__Clostridiales; f__Lachnospiraceae; g__Blautia; s__	1.317782308	0.003744642
4468891	k__Bacteria; p__Firmicutes; c__Erysipelotrichi; o__Erysipelotrichales; f__Erysipelotrichaceae; g__; s__	1.314984087	0.0037991
192070	k__Bacteria; p__Bacteroidetes; c__Bacteroidia; o__Bacteroidales; f__Bacteroidaceae; g__Bacteroides; s__	1.341780105	0.003803836
359149	k__Bacteria; p__Firmicutes; c__Clostridia; o__Clostridiales; f__; g__; s__	1.266529368	0.003856022
171559	k__Bacteria; p__Bacteroidetes; c__Bacteroidia; o__Bacteroidales; f__Bacteroidaceae; g__Bacteroides; s__	1.364186883	0.003880035
170462	k__Bacteria; p__Firmicutes; c__Clostridia; o__Clostridiales; f__Lachnospiraceae; g__[Ruminococcus]; s__	1.547625959	0.003890726
182483	k__Bacteria; p__Firmicutes; c__Erysipelotrichi; o__Erysipelotrichales; f__Erysipelotrichaceae; g__[Eubacterium]; s__biforme	1.585436803	0.003892592
4393532	k__Bacteria; p__Actinobacteria; c__Coriobacteriia; o__Coriobacteriales; f__Coriobacteriaceae; g__Eggerthella; s__lenta	1.350570095	0.003900497
158625	k__Bacteria; p__Firmicutes; c__Clostridia; o__Clostridiales; f__Ruminococcaceae; g__; s__	-1.576663057	0.003982367
329096	k__Bacteria; p__Proteobacteria; c__Gammaproteobacteria; o__Enterobacteriales; f__Enterobacteriaceae; g__; s__	1.432523361	0.003999224
306315	k__Bacteria; p__Firmicutes; c__Clostridia; o__Clostridiales; f__Ruminococcaceae; g__Oscillospira; s__	1.472306676	0.003999224
178018	k__Bacteria; p__Firmicutes; c__Clostridia; o__Clostridiales; f__Lachnospiraceae; g__; s__	1.419028998	0.004027308
772282	k__Bacteria; p__Bacteroidetes; c__Bacteroidia; o__Bacteroidales; f__Rikenellaceae; g__; s__	1.367694646	0.004101308
362568	k__Bacteria; p__Firmicutes; c__Clostridia; o__Clostridiales; f__Lachnospiraceae; g__Blautia; s__	-1.503877475	0.004109473
4469492	k__Bacteria; p__Proteobacteria; c__Betaproteobacteria; o__Rhodocyclales; f__Rhodocyclaceae; g__; s__	-1.50574219	0.004160405
4469233	k__Bacteria; p__Firmicutes; c__Clostridia; o__Clostridiales; f__; g__; s__	1.53369041	0.004160405
183169	k__Bacteria; p__Firmicutes; c__Clostridia; o__Clostridiales; f__; g__; s__	1.332290004	0.004210132
54563	k__Bacteria; p__Firmicutes; c__Clostridia; o__Clostridiales; f__Lachnospiraceae; g__[Ruminococcus]; s__gnavus	1.418917112	0.00423129
3855938	k__Bacteria; p__Firmicutes; c__Erysipelotrichi; o__Erysipelotrichales; f__Erysipelotrichaceae; g__Coprobacillus; s__	1.492743868	0.00423129
293307	k__Bacteria; p__Firmicutes; c__Clostridia; o__Clostridiales; f__Lachnospiraceae; g__Blautia; s__	-1.578217089	0.004332644
338301	k__Bacteria; p__Firmicutes; c__Clostridia; o__Clostridiales; f__; g__; s__	1.521980064	0.004335592
367456	k__Bacteria; p__Firmicutes; c__Clostridia; o__Clostridiales; f__Lachnospiraceae; g__Blautia; s__	-1.449910675	0.004418417
2423305	k__Bacteria; p__Actinobacteria; c__Coriobacteriia; o__Coriobacteriales; f__Coriobacteriaceae; g__; s__	1.388405765	0.004421945
319621	k__Bacteria; p__Bacteroidetes; c__Bacteroidia; o__Bacteroidales; f__Bacteroidaceae; g__Bacteroides; s__	1.553557518	0.004500385
4475758	k__Bacteria; p__Firmicutes; c__Clostridia; o__Clostridiales; f__Veillonellaceae; g__Veillonella; s__dispar	1.405495502	0.004533804
194758	k__Bacteria; p__Firmicutes; c__Clostridia; o__Clostridiales; f__Lachnospiraceae; g__Coprococcus; s__	1.295072116	0.00456376
176312	k__Bacteria; p__Firmicutes; c__Clostridia; o__Clostridiales; f__; g__; s__	1.32745986	0.004580931
328905	k__Bacteria; p__Firmicutes; c__Clostridia; o__Clostridiales; f__Ruminococcaceae; g__Oscillospira; s__	-1.427059686	0.004583513

197696	k__Bacteria; p__Firmicutes; c__Clostridia; o__Clostridiales; f__Ruminococcaceae; g__; s__	1.408099834	0.004592135
4344883	k__Bacteria; p__Firmicutes; c__Clostridia; o__Clostridiales; f__Clostridiaceae; g__; s__	-1.417645825	0.004665727
176450	k__Bacteria; p__Firmicutes; c__Clostridia; o__Clostridiales; f__Lachnospiraceae; g__Blautia; s__	1.300069813	0.004688255
4325261	k__Bacteria; p__Firmicutes; c__Clostridia; o__Clostridiales; f__Lachnospiraceae; g__; s__	-1.599022845	0.004720428
4372382	k__Bacteria; p__Firmicutes; c__Clostridia; o__Clostridiales; f__Lachnospiraceae; g__; s__	-1.450954772	0.00478819
191601	k__Bacteria; p__Firmicutes; c__Clostridia; o__Clostridiales; f__Lachnospiraceae; g__Blautia; s__	-1.685331413	0.004849989
182289	k__Bacteria; p__Firmicutes; c__Clostridia; o__Clostridiales; f__Lachnospiraceae; g__Coprococcus; s__	-1.296097194	0.00492475
340711	k__Bacteria; p__Firmicutes; c__Clostridia; o__Clostridiales; f__Ruminococcaceae; g__; s__	1.362743763	0.005044067
801210	k__Bacteria; p__Proteobacteria; c__Betaproteobacteria; o__Burkholderiales; f__Alcaligenaceae; g__Sutterella; s__	1.57083013	0.005085016
181422	k__Bacteria; p__Firmicutes; c__Clostridia; o__Clostridiales; f__Ruminococcaceae; g__Faecalibacterium; s__prausnitzii	1.278439453	0.005209064
195947	k__Bacteria; p__Firmicutes; c__Clostridia; o__Clostridiales; f__Ruminococcaceae; g__Ruminococcus; s__	1.397038867	0.005230599
163243	k__Bacteria; p__Firmicutes; c__Clostridia; o__Clostridiales; f__Ruminococcaceae; g__Ruminococcus; s__	-1.628960076	0.005280143
3450453	k__Bacteria; p__Firmicutes; c__Clostridia; o__Clostridiales; f__Lachnospiraceae; g__Blautia; s__	-1.427755056	0.005297291
2148365	k__Bacteria; p__Firmicutes; c__Clostridia; o__Clostridiales; f__Lachnospiraceae; g__; s__	-1.295299775	0.005616292
212686	k__Bacteria; p__Firmicutes; c__Clostridia; o__Clostridiales; f__Ruminococcaceae; g__Oscillospira; s__	1.307860258	0.00562945
194215	k__Bacteria; p__Firmicutes; c__Clostridia; o__Clostridiales; f__Ruminococcaceae; g__; s__	1.329196639	0.005682975
1066814	k__Bacteria; p__Bacteroidetes; c__Bacteroidia; o__Bacteroidales; f__Prevotellaceae; g__Prevotella; s__	1.428596771	0.005682975
338105	k__Bacteria; p__Firmicutes; c__Clostridia; o__Clostridiales; f__Christensenellaceae; g__; s__	1.401813129	0.005789098
268978	k__Bacteria; p__Firmicutes; c__Clostridia; o__Clostridiales; f__Lachnospiraceae; g__; s__	-1.29514459	0.005828348
3090117	k__Bacteria; p__Bacteroidetes; c__Bacteroidia; o__Bacteroidales; f__[Barnesiellaceae]; g__; s__	1.475378117	0.00590636
4459733	k__Bacteria; p__Firmicutes; c__Clostridia; o__Clostridiales; f__Clostridiaceae; g__; s__	-1.566282697	0.005954253
579244	k__Bacteria; p__Firmicutes; c__Clostridia; o__Clostridiales; f__Ruminococcaceae; g__Oscillospira; s__	-1.426862981	0.006002664
4454025	k__Bacteria; p__Firmicutes; c__Clostridia; o__Clostridiales; f__Lachnospiraceae; g__; s__	1.370832388	0.006039824
312882	k__Bacteria; p__Firmicutes; c__Clostridia; o__Clostridiales; f__Ruminococcaceae; g__; s__	1.161096871	0.006050568
174831	k__Bacteria; p__Bacteroidetes; c__Bacteroidia; o__Bacteroidales; f__Prevotellaceae; g__Prevotella; s__copri	1.360424863	0.00622264
356745	k__Bacteria; p__Firmicutes; c__Clostridia; o__Clostridiales; f__Ruminococcaceae; g__Ruminococcus; s__	-1.504686048	0.006240771
4359056	k__Bacteria; p__Bacteroidetes; c__Bacteroidia; o__Bacteroidales; f__Bacteroidaceae; g__Bacteroides; s__	1.403196728	0.006273883
72820	k__Bacteria; p__Actinobacteria; c__Actinobacteria; o__Bifidobacteriales; f__Bifidobacteriaceae; g__Bifidobacterium; s__longum	-1.238821971	0.006481913
190441	k__Bacteria; p__Firmicutes; c__Clostridia; o__Clostridiales; f__Lachnospiraceae; g__; s__	1.269467412	0.006583518
189235	k__Bacteria; p__Firmicutes; c__Clostridia; o__Clostridiales; f__Ruminococcaceae; g__; s__	1.423543803	0.00679205
192308	k__Bacteria; p__Firmicutes; c__Clostridia; o__Clostridiales; f__; g__; s__	-1.405193915	0.006828414
4375000	k__Bacteria; p__Proteobacteria; c__Gammaproteobacteria; o__Enterobacteriales; f__Enterobacteriaceae; g__; s__	1.390376037	0.006828414
193709	k__Bacteria; p__Firmicutes; c__Clostridia; o__Clostridiales; f__Ruminococcaceae; g__; s__	1.690191695	0.006834449
1696853	k__Bacteria; p__Firmicutes; c__Bacilli; o__Lactobacillales; f__Carnobacteriaceae; g__Granulicatella; s__	1.184500347	0.006987832
258099	k__Bacteria; p__Firmicutes; c__Clostridia; o__Clostridiales; f__Ruminococcaceae; g__; s__	1.353306353	0.007015544
851634	k__Bacteria; p__Bacteroidetes; c__Bacteroidia; o__Bacteroidales; f__Prevotellaceae; g__Prevotella; s__	1.34530585	0.00712974
582691	k__Bacteria; p__Firmicutes; c__Clostridia; o__Clostridiales; f__Clostridiaceae; g__; s__	-1.266183122	0.007185254
173900	k__Bacteria; p__Firmicutes; c__Clostridia; o__Clostridiales; f__Lachnospiraceae; g__; s__	1.310030484	0.007348252
4461030	k__Bacteria; p__Proteobacteria; c__Gammaproteobacteria; o__Enterobacteriales; f__Enterobacteriaceae; g__; s__	1.340584413	0.007367282
177150	k__Bacteria; p__Bacteroidetes; c__Bacteroidia; o__Bacteroidales; f__Bacteroidaceae; g__Bacteroides; s__	1.208433944	0.007369354
505670	k__Bacteria; p__Firmicutes; c__Clostridia; o__Clostridiales; f__[Tissierellaceae]; g__WAL_1855D; s__	1.216711497	0.007381683
495007	k__Bacteria; p__Firmicutes; c__Clostridia; o__Clostridiales; f__[Tissierellaceae]; g__ph2; s__	1.349727678	0.007471059
1142110	k__Bacteria; p__Firmicutes; c__Clostridia; o__Clostridiales; f__Peptostreptococcaceae; g__; s__	-1.238466382	0.007472206
13986	k__Bacteria; p__Firmicutes; c__Clostridia; o__Clostridiales; f__Lachnospiraceae; g__; s__	1.291529892	0.007472206
178497	k__Bacteria; p__Firmicutes; c__Clostridia; o__Clostridiales; f__Ruminococcaceae; g__; s__	1.405347806	0.007472206
197460	k__Bacteria; p__Firmicutes; c__Clostridia; o__Clostridiales; f__Lachnospiraceae; g__; s__	1.186771525	0.00758671
175836	k__Bacteria; p__Firmicutes; c__Clostridia; o__Clostridiales; f__Ruminococcaceae; g__; s__	1.285165538	0.007609263

185836	k__Bacteria; p__Firmicutes; c__Clostridia; o__Clostridiales; f__Lachnospiraceae; g__Blautia; s__	-1.320176077	0.007632422
179609	k__Bacteria; p__Firmicutes; c__Clostridia; o__Clostridiales; f__Lachnospiraceae; g__; s__	1.216367286	0.00791985
104135	k__Bacteria; p__Actinobacteria; c__Actinobacteria; o__Actinomycetales; f__Actinomycetaceae; g__Mobiluncus; s__	1.274338089	0.008045394
4452107	k__Bacteria; p__Firmicutes; c__Clostridia; o__Clostridiales; f__Lachnospiraceae; g__; s__	1.156370494	0.008073638
179625	k__Bacteria; p__Firmicutes; c__Clostridia; o__Clostridiales; f__Lachnospiraceae; g__; s__	1.209107731	0.008122034
198128	k__Bacteria; p__Firmicutes; c__Clostridia; o__Clostridiales; f__Lachnospiraceae; g__Blautia; s__	1.209312233	0.008153721
997267	k__Bacteria; p__Firmicutes; c__Clostridia; o__Clostridiales; f__Ruminococcaceae; g__; s__	1.342769107	0.008176133
839684	k__Bacteria; p__Firmicutes; c__Clostridia; o__Clostridiales; f__Lachnospiraceae; g__[Ruminococcus]; s__	1.361269623	0.008176133
190233	k__Bacteria; p__Bacteroidetes; c__Bacteroidia; o__Bacteroidales; f__Prevotellaceae; g__Prevotella; s__	1.299210311	0.008178394
548587	k__Bacteria; p__Firmicutes; c__Erysipelotrichi; o__Erysipelotrichales; f__Erysipelotrichaceae; g__[Eubacterium]; s__dolichum	1.237932844	0.00821559
1928156	k__Bacteria; p__Firmicutes; c__Clostridia; o__Clostridiales; f__Lachnospiraceae; g__Roseburia; s__	1.255957268	0.00821559
2256425	k__Bacteria; p__Firmicutes; c__Clostridia; o__Clostridiales; f__Christensenellaceae; g__; s__	1.282413565	0.00821559
3028273	k__Bacteria; p__Firmicutes; c__Clostridia; o__Clostridiales; f__; g__; s__	1.32817221	0.008415516
183054	k__Bacteria; p__Firmicutes; c__Clostridia; o__Clostridiales; f__Lachnospiraceae; g__; s__	1.284905516	0.008616509
4391262	k__Bacteria; p__Proteobacteria; c__Gammaproteobacteria; o__Enterobacteriales; f__Enterobacteriaceae; g__; s__	-1.178043095	0.008777397
194036	k__Bacteria; p__Firmicutes; c__Clostridia; o__Clostridiales; f__Ruminococcaceae; g__; s__	-1.290827444	0.008848263
189877	k__Bacteria; p__Firmicutes; c__Clostridia; o__Clostridiales; f__Lachnospiraceae; g__Blautia; s__	-1.317707105	0.008993817
4475642	k__Bacteria; p__Bacteroidetes; c__Bacteroidia; o__Bacteroidales; f__Bacteroidaceae; g__Bacteroides; s__	1.22848442	0.009310808
4433737	k__Bacteria; p__Firmicutes; c__Clostridia; o__Clostridiales; f__Lachnospiraceae; g__; s__	1.190912549	0.009411322
4371463	k__Bacteria; p__Firmicutes; c__Clostridia; o__Clostridiales; f__Lachnospiraceae; g__; s__	1.152258155	0.00948101
186389	k__Bacteria; p__Firmicutes; c__Clostridia; o__Clostridiales; f__Clostridiaceae; g__Clostridium; s__	1.232915434	0.009826034
187035	k__Bacteria; p__Firmicutes; c__Clostridia; o__Clostridiales; f__Lachnospiraceae; g__Blautia; s__	-1.447918363	0.009835855
362955	k__Bacteria; p__Firmicutes; c__Clostridia; o__Clostridiales; f__; g__; s__	1.331135029	0.009907801
189067	k__Bacteria; p__Firmicutes; c__Clostridia; o__Clostridiales; f__Lachnospiraceae; g__Blautia; s__	-1.352666772	0.010037536
213394	k__Bacteria; p__Firmicutes; c__Clostridia; o__Clostridiales; f__Lachnospiraceae; g__Lachnospira; s__	1.314552969	0.010271891
4409280	k__Bacteria; p__Firmicutes; c__Clostridia; o__Clostridiales; f__Ruminococcaceae; g__; s__	-1.264241144	0.010318
692756	k__Bacteria; p__Bacteroidetes; c__Bacteroidia; o__Bacteroidales; f__Porphyromonadaceae; g__Porphyromonas; s__	1.227261049	0.010346708
180523	k__Bacteria; p__Firmicutes; c__Clostridia; o__Clostridiales; f__Lachnospiraceae; g__; s__	1.123955668	0.010389022
189292	k__Bacteria; p__Firmicutes; c__Clostridia; o__Clostridiales; f__Lachnospiraceae; g__; s__	1.197855712	0.010394546
563803	k__Bacteria; p__Firmicutes; c__Clostridia; o__Clostridiales; f__Lachnospiraceae; g__; s__	1.305634389	0.010395236
4376828	k__Bacteria; p__Actinobacteria; c__Actinobacteria; o__Bifidobacteriales; f__Bifidobacteriaceae; g__Bifidobacterium; s__	1.175549517	0.010534034
184000	k__Bacteria; p__Firmicutes; c__Clostridia; o__Clostridiales; f__Ruminococcaceae; g__Faecalibacterium; s__prausnitzii	1.131452508	0.0108654
4359590	k__Bacteria; p__Firmicutes; c__Bacilli; o__Lactobacillales; f__Enterococcaceae; g__Enterococcus; s__	1.262109445	0.010872721
184910	k__Bacteria; p__Firmicutes; c__Clostridia; o__Clostridiales; f__Lachnospiraceae; g__; s__	1.131106282	0.010955148
185715	k__Bacteria; p__Firmicutes; c__Clostridia; o__Clostridiales; f__Lachnospiraceae; g__Coprococcus; s__	1.176827732	0.010955148
300662	k__Bacteria; p__Firmicutes; c__Clostridia; o__Clostridiales; f__Clostridiaceae; g__Sarcina; s__	1.224614268	0.01096498
174611	k__Bacteria; p__Firmicutes; c__Clostridia; o__Clostridiales; f__Ruminococcaceae; g__Faecalibacterium; s__prausnitzii	-1.264072423	0.010978967
4345285	k__Bacteria; p__Firmicutes; c__Bacilli; o__Bacillales; f__Staphylococcaceae; g__Staphylococcus; s__	-1.115192413	0.010978967
183579	k__Bacteria; p__Bacteroidetes; c__Bacteroidia; o__Bacteroidales; f__Bacteroidaceae; g__Bacteroides; s__	1.206063756	0.011083523
4421070	k__Bacteria; p__Bacteroidetes; c__Bacteroidia; o__Bacteroidales; f__Bacteroidaceae; g__Bacteroides; s__	1.295548163	0.011093917
290241	k__Bacteria; p__Firmicutes; c__Clostridia; o__Clostridiales; f__Clostridiaceae; g__Clostridium; s__perfringens	-1.176021977	0.011236182
44151	k__Bacteria; p__Firmicutes; c__Clostridia; o__Clostridiales; f__Ruminococcaceae; g__; s__	1.150330534	0.011496204
165046	k__Bacteria; p__Firmicutes; c__Clostridia; o__Clostridiales; f__Ruminococcaceae; g__Oscillospira; s__	1.170084008	0.011533647
189760	k__Bacteria; p__Firmicutes; c__Clostridia; o__Clostridiales; f__Ruminococcaceae; g__; s__	1.078043018	0.012024593
4336939	k__Bacteria; p__Firmicutes; c__Clostridia; o__Clostridiales; f__Ruminococcaceae; g__; s__	1.352339367	0.012288429
4453535	k__Bacteria; p__Firmicutes; c__Bacilli; o__Gemellales; f__Gemellaceae; g__; s__	1.208072136	0.012288629
553150	k__Bacteria; p__Firmicutes; c__Clostridia; o__Clostridiales; f__Lachnospiraceae; g__Coprococcus; s__	1.254284004	0.012305615

4094866	k__Bacteria; p__Firmicutes; c__Clostridia; o__Clostridiales; f__Lachnospiraceae; g__; s__	-1.114237905	0.012454089
193797	k__Bacteria; p__Firmicutes; c__Clostridia; o__Clostridiales; f__; g__; s__	1.241575947	0.012454089
4477479	k__Bacteria; p__Firmicutes; c__Clostridia; o__Clostridiales; f__Lachnospiraceae; g__[Ruminococcus]; s__	1.227803977	0.012717616
197367	k__Bacteria; p__Bacteroidetes; c__Bacteroidia; o__Bacteroidales; f__Bacteroidaceae; g__Bacteroides; s__	1.123181156	0.013113083
173245	k__Bacteria; p__Firmicutes; c__Clostridia; o__Clostridiales; f__; g__; s__	1.244723348	0.01337129
563572	k__Bacteria; p__Firmicutes; c__Clostridia; o__Clostridiales; f__Lachnospiraceae; g__; s__	1.093441473	0.013824076
328059	k__Bacteria; p__Firmicutes; c__Clostridia; o__Clostridiales; f__Clostridiaceae; g__; s__	-1.263910485	0.014076024
143423	k__Bacteria; p__Firmicutes; c__Clostridia; o__Clostridiales; f__Ruminococcaceae; g__; s__	1.243809888	0.014076024
253584	k__Bacteria; p__Proteobacteria; c__Epsilonproteobacteria; o__Campylobacterales; f__Campylobacteraceae; g__Campylobacter; s__	1.178080773	0.014097602
4347520	k__Bacteria; p__Firmicutes; c__Clostridia; o__Clostridiales; f__; g__; s__	1.124198682	0.014101576
4256470	k__Bacteria; p__Bacteroidetes; c__Bacteroidia; o__Bacteroidales; f__Bacteroidaceae; g__Bacteroides; s__ovatus	-1.174523878	0.014461911
182133	k__Bacteria; p__Firmicutes; c__Clostridia; o__Clostridiales; f__Lachnospiraceae; g__Blautia; s__	-1.201603798	0.014493881
179384	k__Bacteria; p__Firmicutes; c__Clostridia; o__Clostridiales; f__Lachnospiraceae; g__; s__	1.089614821	0.014633557
174614	k__Bacteria; p__Firmicutes; c__Clostridia; o__Clostridiales; f__Ruminococcaceae; g__Ruminococcus; s__	-1.14549767	0.014636162
2403301	k__Bacteria; p__Firmicutes; c__Clostridia; o__Clostridiales; f__Lachnospiraceae; g__; s__	1.213299012	0.014820597
2132002	k__Bacteria; p__Firmicutes; c__Clostridia; o__Clostridiales; f__Lachnospiraceae; g__; s__	1.305416789	0.014820597
4471245	k__Bacteria; p__Firmicutes; c__Clostridia; o__Clostridiales; f__Lachnospiraceae; g__Dorea; s__	1.203841462	0.0148766
340547	k__Bacteria; p__Bacteroidetes; c__Bacteroidia; o__Bacteroidales; f__Bacteroidaceae; g__Bacteroides; s__	1.243786467	0.014944812
3390534	k__Bacteria; p__Firmicutes; c__Clostridia; o__Clostridiales; f__Ruminococcaceae; g__; s__	1.153616258	0.01516727
836693	k__Bacteria; p__Firmicutes; c__Clostridia; o__SHA-98; f__; g__; s__	1.173965078	0.01516727
174439	k__Bacteria; p__Firmicutes; c__Clostridia; o__Clostridiales; f__Ruminococcaceae; g__Faecalibacterium; s__prausnitzii	1.064022756	0.01517775
176507	k__Bacteria; p__Firmicutes; c__Clostridia; o__Clostridiales; f__Ruminococcaceae; g__; s__	-1.254162716	0.015433428
175612	k__Bacteria; p__Firmicutes; c__Clostridia; o__Clostridiales; f__Lachnospiraceae; g__Roseburia; s__	1.061995754	0.015433428
296394	k__Bacteria; p__Firmicutes; c__Clostridia; o__Clostridiales; f__Ruminococcaceae; g__; s__	1.203686935	0.015433428
4456702	k__Bacteria; p__Firmicutes; c__Clostridia; o__Clostridiales; f__Ruminococcaceae; g__Ruminococcus; s__	1.145742165	0.015570066
166164	k__Bacteria; p__Firmicutes; c__Clostridia; o__Clostridiales; f__Ruminococcaceae; g__Ruminococcus; s__	1.218040018	0.01557665
1115481	k__Bacteria; p__Firmicutes; c__Clostridia; o__Clostridiales; f__Clostridiaceae; g__Clostridium; s__	-1.205148016	0.015857357
4374084	k__Bacteria; p__Bacteroidetes; c__Bacteroidia; o__Bacteroidales; f__Porphyromonadaceae; g__Parabacteroides; s__distasonis	1.171244957	0.015857357
519880	k__Bacteria; p__Firmicutes; c__Clostridia; o__Clostridiales; f__Ruminococcaceae; g__; s__	1.17191505	0.015857357
176318	k__Bacteria; p__Firmicutes; c__Clostridia; o__Clostridiales; f__Christensenellaceae; g__; s__	1.190314908	0.015857357
807548	k__Bacteria; p__Firmicutes; c__Erysipelotrichi; o__Erysipelotrichales; f__Erysipelotrichaceae; g__cc_115; s__	1.214175088	0.01611174
591399	k__Bacteria; p__Firmicutes; c__Clostridia; o__Clostridiales; f__Lachnospiraceae; g__Dorea; s__	1.166449485	0.016230261
191734	k__Bacteria; p__Firmicutes; c__Clostridia; o__Clostridiales; f__Lachnospiraceae; g__Blautia; s__	-1.111545232	0.016265439
4380813	k__Bacteria; p__Firmicutes; c__Clostridia; o__Clostridiales; f__Lachnospiraceae; g__Roseburia; s__	1.066234062	0.01629752
1848900	k__Bacteria; p__Firmicutes; c__Clostridia; o__Clostridiales; f__Lachnospiraceae; g__; s__	1.059323243	0.016512806
4414044	k__Bacteria; p__Firmicutes; c__Clostridia; o__Clostridiales; f__Lachnospiraceae; g__Roseburia; s__	1.059063478	0.016652179
337511	k__Bacteria; p__Firmicutes; c__Clostridia; o__Clostridiales; f__Clostridiaceae; g__; s__	1.122837757	0.016795975
2066056	k__Bacteria; p__Firmicutes; c__Clostridia; o__Clostridiales; f__Ruminococcaceae; g__; s__	1.103884511	0.016801519
4424113	k__Bacteria; p__Firmicutes; c__Clostridia; o__Clostridiales; f__; g__; s__	1.092243632	0.017151556
4458959	k__Bacteria; p__Firmicutes; c__Clostridia; o__Clostridiales; f__Veillonellaceae; g__Veillonella; s__	-1.079803672	0.017230732
304047	k__Bacteria; p__Bacteroidetes; c__Bacteroidia; o__Bacteroidales; f__Bacteroidaceae; g__Bacteroides; s__acidifaciens	-1.113150944	0.017815403
194534	k__Bacteria; p__Firmicutes; c__Clostridia; o__Clostridiales; f__Ruminococcaceae; g__; s__	-1.170129387	0.018110238
845544	k__Bacteria; p__Firmicutes; c__Clostridia; o__Clostridiales; f__Ruminococcaceae; g__Faecalibacterium; s__prausnitzii	1.031626261	0.01825608
4328910	k__Bacteria; p__Firmicutes; c__Clostridia; o__Clostridiales; f__Veillonellaceae; g__Veillonella; s__parvula	1.06224327	0.01825608
178712	k__Bacteria; p__Firmicutes; c__Clostridia; o__Clostridiales; f__; g__; s__	1.139164663	0.01825608
183030	k__Bacteria; p__Firmicutes; c__Clostridia; o__Clostridiales; f__; g__; s__	1.196504711	0.018356163
187210	k__Bacteria; p__Firmicutes; c__Clostridia; o__Clostridiales; f__Lachnospiraceae; g__Blautia; s__	-1.143799349	0.01842732

282360	k__Bacteria; p__Actinobacteria; c__Actinobacteria; o__Actinomycetales; f__Corynebacteriaceae; g__Corynebacterium; s__	1.162302581	0.018582
233253	k__Bacteria; p__Firmicutes; c__Clostridia; o__Clostridiales; f__Ruminococcaceae; g__; s__	1.034959273	0.018663531
246717	k__Bacteria; p__Proteobacteria; c__Epsilonproteobacteria; o__Campylobacterales; f__Campylobacteraceae; g__Campylobacter; s__	1.165110557	0.018719334
587933	k__Bacteria; p__Firmicutes; c__Erysipelotrichi; o__Erysipelotrichales; f__Erysipelotrichaceae; g__Coprobacillus; s__	1.150164542	0.019171887
292758	k__Bacteria; p__Firmicutes; c__Clostridia; o__Clostridiales; f__; g__; s__	1.148613565	0.019340812
178760	k__Bacteria; p__Firmicutes; c__Clostridia; o__Clostridiales; f__; g__; s__	-1.393574385	0.019885006
114348	k__Bacteria; p__Firmicutes; c__Clostridia; o__Clostridiales; f__Ruminococcaceae; g__Oscillospira; s__	1.032243266	0.02026873
174911	k__Bacteria; p__Firmicutes; c__Clostridia; o__Clostridiales; f__Lachnospiraceae; g__; s__	1.090279563	0.020865898
358939	k__Bacteria; p__Firmicutes; c__Clostridia; o__Clostridiales; f__Lachnospiraceae; g__; s__	-1.145826816	0.020930605
4443143	k__Bacteria; p__Firmicutes; c__Clostridia; o__Clostridiales; f__Ruminococcaceae; g__Ruminococcus; s__	1.148740196	0.020930605
840376	k__Bacteria; p__Firmicutes; c__Clostridia; o__Clostridiales; f__Ruminococcaceae; g__; s__	1.16141362	0.021067573
4330852	k__Bacteria; p__Firmicutes; c__Clostridia; o__Clostridiales; f__Ruminococcaceae; g__Oscillospira; s__	1.089799846	0.021279647
527988	k__Bacteria; p__Firmicutes; c__Clostridia; o__Clostridiales; f__Eubacteriaceae; g__Anaerofustis; s__	-1.171539744	0.021655844
175922	k__Bacteria; p__Firmicutes; c__Clostridia; o__Clostridiales; f__Ruminococcaceae; g__Oscillospira; s__	-1.011224807	0.021655844
302352	k__Bacteria; p__Firmicutes; c__Clostridia; o__Clostridiales; f__Lachnospiraceae; g__; s__	1.004555816	0.021655844
121873	k__Bacteria; p__Firmicutes; c__Clostridia; o__Clostridiales; f__Dehalobacteriaceae; g__Dehalobacterium; s__	1.057029336	0.022355981
4374302	k__Bacteria; p__Firmicutes; c__Clostridia; o__Clostridiales; f__Lachnospiraceae; g__Dorea; s__	-1.013607656	0.022588053
4378683	k__Bacteria; p__Firmicutes; c__Clostridia; o__Clostridiales; f__Lachnospiraceae; g__; s__	-0.951303117	0.022633214
113417	k__Bacteria; p__Proteobacteria; c__Deltaproteobacteria; o__Desulfovibrionales; f__Desulfovibrionaceae; g__; s__	1.125851745	0.022649453
174019	k__Bacteria; p__Firmicutes; c__Clostridia; o__Clostridiales; f__Lachnospiraceae; g__Coprococcus; s__	1.262418398	0.022712017
188225	k__Bacteria; p__Firmicutes; c__Clostridia; o__Clostridiales; f__Ruminococcaceae; g__; s__	1.054238746	0.023319467
352014	k__Bacteria; p__Firmicutes; c__Clostridia; o__Clostridiales; f__Ruminococcaceae; g__; s__	1.056122025	0.023319467
340876	k__Bacteria; p__Firmicutes; c__Clostridia; o__Clostridiales; f__; g__; s__	1.135320425	0.023319467
4381422	k__Bacteria; p__Bacteroidetes; c__Bacteroidia; o__Bacteroidales; f__Bacteroidaceae; g__Bacteroides; s__	1.106154843	0.024685115
151870	k__Bacteria; p__Firmicutes; c__Erysipelotrichi; o__Erysipelotrichales; f__Erysipelotrichaceae; g__Coprobacillus; s__	1.117897299	0.024762074
191872	k__Bacteria; p__Firmicutes; c__Clostridia; o__Clostridiales; f__Ruminococcaceae; g__; s__	0.996496674	0.025241175
187780	k__Bacteria; p__Firmicutes; c__Clostridia; o__Clostridiales; f__Ruminococcaceae; g__; s__	1.14929537	0.025241175
1082607	k__Bacteria; p__Actinobacteria; c__Actinobacteria; o__Actinomycetales; f__Corynebacteriaceae; g__Corynebacterium; s__	1.004164688	0.025353299
81152	k__Bacteria; p__Firmicutes; c__Clostridia; o__Clostridiales; f__Lachnospiraceae; g__Blautia; s__	1.001179982	0.02536821
343322	k__Bacteria; p__Firmicutes; c__Clostridia; o__Clostridiales; f__Clostridiaceae; g__; s__	-1.079624817	0.025540741
105813	k__Bacteria; p__Firmicutes; c__Clostridia; o__Clostridiales; f__; g__; s__	1.104690231	0.025540741
359762	k__Bacteria; p__Firmicutes; c__Clostridia; o__Clostridiales; f__; g__; s__	1.113909947	0.026272081
197077	k__Bacteria; p__Firmicutes; c__Clostridia; o__Clostridiales; f__Lachnospiraceae; g__Roseburia; s__	0.968092532	0.027506388
368236	k__Bacteria; p__Firmicutes; c__Clostridia; o__Clostridiales; f__Ruminococcaceae; g__; s__	0.969736044	0.027506388
4346677	k__Bacteria; p__Bacteroidetes; c__Bacteroidia; o__Bacteroidales; f__Bacteroidaceae; g__Bacteroides; s__	0.997110338	0.027506388
308498	k__Bacteria; p__Firmicutes; c__Clostridia; o__Clostridiales; f__Ruminococcaceae; g__; s__	0.99968072	0.027646895
3565293	k__Bacteria; p__Bacteroidetes; c__Bacteroidia; o__Bacteroidales; f__Rikenellaceae; g__; s__	1.136317582	0.027703634
828483	k__Bacteria; p__Firmicutes; c__Clostridia; o__Clostridiales; f__Clostridiaceae; g__Clostridium; s__perfringens	-1.013540022	0.02783622
3557915	k__Bacteria; p__Firmicutes; c__Clostridia; o__Clostridiales; f__Lachnospiraceae; g__; s__	1.007003941	0.02783622
503197	k__Bacteria; p__Firmicutes; c__Clostridia; o__Clostridiales; f__[Tissierellaceae]; g__Anaerococcus; s__	0.994695794	0.027893369
336205	k__Bacteria; p__Firmicutes; c__Clostridia; o__Clostridiales; f__Lachnospiraceae; g__Coprococcus; s__	0.965144633	0.027908986
4420669	k__Bacteria; p__Bacteroidetes; c__Bacteroidia; o__Bacteroidales; f__[Odoribacteraceae]; g__Butyricimonas; s__	1.064211844	0.027918089
182116	k__Bacteria; p__Firmicutes; c__Clostridia; o__Clostridiales; f__Lachnospiraceae; g__; s__	-1.108143617	0.028284306
4379449	k__Bacteria; p__Firmicutes; c__Erysipelotrichi; o__Erysipelotrichales; f__Erysipelotrichaceae; g__Clostridium; s__saccharogumia	1.037948464	0.02838867
269386	k__Bacteria; p__Cyanobacteria; c__4C0d-2; o__YS2; f__; g__; s__	1.006916447	0.028910663
4402537	k__Bacteria; p__Actinobacteria; c__Coriobacteriia; o__Coriobacteriales; f__Coriobacteriaceae; g__; s__	1.007655812	0.028910663
310979	k__Bacteria; p__Firmicutes; c__Clostridia; o__Clostridiales; f__; g__; s__	1.021457586	0.028910663

182184	k__Bacteria; p__Firmicutes; c__Clostridia; o__Clostridiales; f__Ruminococcaceae; g__; s__	0.9642308	0.029398002
619817	k__Bacteria; p__Firmicutes; c__Clostridia; o__Clostridiales; f__Lachnospiraceae; g__Anaerostipes; s__	1.006176713	0.029398002
583134	k__Bacteria; p__Firmicutes; c__Clostridia; o__Clostridiales; f__Ruminococcaceae; g__; s__	1.050006129	0.029398002
186104	k__Bacteria; p__Firmicutes; c__Clostridia; o__Clostridiales; f__Ruminococcaceae; g__; s__	1.001981629	0.030131854
337909	k__Bacteria; p__Firmicutes; c__Clostridia; o__Clostridiales; f__Clostridiaceae; g__; s__	-1.079767404	0.030668657
4030157	k__Bacteria; p__Firmicutes; c__Clostridia; o__Clostridiales; f__Lachnospiraceae; g__[Ruminococcus]; s__	1.031293271	0.030668657
360660	k__Bacteria; p__Firmicutes; c__Clostridia; o__Clostridiales; f__Lachnospiraceae; g__Blautia; s__	-1.063879825	0.030775961
3841096	k__Bacteria; p__Firmicutes; c__Clostridia; o__Clostridiales; f__Ruminococcaceae; g__Oscillospira; s__	1.028424305	0.030775961
204126	k__Bacteria; p__Firmicutes; c__Clostridia; o__Clostridiales; f__Ruminococcaceae; g__Oscillospira; s__	0.963551385	0.031272768
919806	k__Bacteria; p__Firmicutes; c__Clostridia; o__Clostridiales; f__Peptostreptococcaceae; g__; s__	-1.012125178	0.032041788
4426051	k__Bacteria; p__Firmicutes; c__Clostridia; o__Clostridiales; f__Lachnospiraceae; g__[Ruminococcus]; s__gnavus	0.933977071	0.032275926
4344207	k__Bacteria; p__Firmicutes; c__Bacilli; o__Lactobacillales; f__Streptococcaceae; g__Streptococcus; s__anginosus	1.022235995	0.032275926
4477696	k__Bacteria; p__Proteobacteria; c__Gammaproteobacteria; o__Pasteurellales; f__Pasteurellaceae; g__Haemophilus; s__parainfluenzae	1.17898892	0.032275926
192444	k__Bacteria; p__Firmicutes; c__Clostridia; o__Clostridiales; f__Lachnospiraceae; g__; s__	-1.013151648	0.032592482
299137	k__Bacteria; p__Firmicutes; c__Clostridia; o__Clostridiales; f__Clostridiaceae; g__; s__	-1.043048441	0.032614569
313274	k__Bacteria; p__Firmicutes; c__Clostridia; o__Clostridiales; f__Ruminococcaceae; g__Oscillospira; s__	-1.010354534	0.032614569
580270	k__Bacteria; p__Firmicutes; c__Clostridia; o__Clostridiales; f__Ruminococcaceae; g__Oscillospira; s__	0.932770041	0.032614569
300491	k__Bacteria; p__Firmicutes; c__Clostridia; o__Clostridiales; f__Ruminococcaceae; g__Oscillospira; s__	0.935180103	0.032995388
130763	k__Bacteria; p__Firmicutes; c__Clostridia; o__Clostridiales; f__Ruminococcaceae; g__; s__	0.96417326	0.035276193
4403113	k__Bacteria; p__Firmicutes; c__Clostridia; o__Clostridiales; f__Ruminococcaceae; g__Faecalibacterium; s__prausnitzii	1.086479777	0.035276193
4466843	k__Bacteria; p__Fusobacteria; c__Fusobacteriia; o__Fusobacteriales; f__Fusobacteriaceae; g__Fusobacterium; s__	0.968290691	0.035848522
4372003	k__Bacteria; p__Bacteroidetes; c__Bacteroidia; o__Bacteroidales; f__Porphyromonadaceae; g__Parabacteroides; s__	1.129981307	0.036069856
4479317	k__Bacteria; p__Firmicutes; c__Clostridia; o__Clostridiales; f__Clostridiaceae; g__Clostridium; s__perfringens	-1.011476005	0.036176204
4332878	k__Bacteria; p__Actinobacteria; c__Coriobacteriia; o__Coriobacteriales; f__Coriobacteriaceae; g__Slackia; s__	0.959878982	0.036176204
424038	k__Bacteria; p__Firmicutes; c__Clostridia; o__Clostridiales; f__[Tissierellaceae]; g__Anaerococcus; s__	0.996246407	0.036191894
4395075	k__Bacteria; p__Bacteroidetes; c__Bacteroidia; o__Bacteroidales; f__Bacteroidaceae; g__Bacteroides; s__ovatus	0.934196143	0.036377367
4329132	k__Bacteria; p__Firmicutes; c__Clostridia; o__Clostridiales; f__Lachnospiraceae; g__; s__	0.900255422	0.036604742
289883	k__Bacteria; p__Firmicutes; c__Bacilli; o__Bacillales; f__Bacillaceae; g__Bacillus; s__	0.991461804	0.03689445
3588390	k__Bacteria; p__Bacteroidetes; c__Bacteroidia; o__Bacteroidales; f__Bacteroidaceae; g__Bacteroides; s__	-0.976050407	0.036928745
234447	k__Bacteria; p__Firmicutes; c__Clostridia; o__Clostridiales; f__Christensenellaceae; g__; s__	0.995676453	0.037516888
1051082	k__Bacteria; p__Firmicutes; c__Clostridia; o__Clostridiales; f__; g__; s__	0.964246855	0.037531772
184996	k__Bacteria; p__Firmicutes; c__Clostridia; o__Clostridiales; f__Ruminococcaceae; g__Faecalibacterium; s__prausnitzii	0.900879997	0.037630633
215231	k__Bacteria; p__Firmicutes; c__Clostridia; o__Clostridiales; f__Ruminococcaceae; g__; s__	0.937005891	0.037721212
180826	k__Bacteria; p__Firmicutes; c__Clostridia; o__Clostridiales; f__Ruminococcaceae; g__Ruminococcus; s__	-0.977283222	0.037975607
190595	k__Bacteria; p__Firmicutes; c__Clostridia; o__Clostridiales; f__; g__; s__	0.92999764	0.037975607
193946	k__Bacteria; p__Firmicutes; c__Clostridia; o__Clostridiales; f__Lachnospiraceae; g__Blautia; s__	-0.941121898	0.038019431
4333921	k__Bacteria; p__Bacteroidetes; c__Bacteroidia; o__Bacteroidales; f__[Odoribacteraceae]; g__Butyricimonas; s__	0.992076462	0.038358406
2309802	k__Bacteria; p__Bacteroidetes; c__Bacteroidia; o__Bacteroidales; f__Porphyromonadaceae; g__Parabacteroides; s__distasonis	0.932805381	0.038421619
221429	k__Bacteria; p__Firmicutes; c__Clostridia; o__Clostridiales; f__Ruminococcaceae; g__; s__	0.993383933	0.038421619
1950496	k__Bacteria; p__Bacteroidetes; c__Bacteroidia; o__Bacteroidales; f__Bacteroidaceae; g__Bacteroides; s__	0.930463571	0.039270857
177893	k__Bacteria; p__Firmicutes; c__Clostridia; o__Clostridiales; f__Ruminococcaceae; g__; s__	0.897170336	0.03931768
845444	k__Bacteria; p__Firmicutes; c__Clostridia; o__Clostridiales; f__Ruminococcaceae; g__; s__	0.963451003	0.03931768
363519	k__Bacteria; p__Firmicutes; c__Clostridia; o__Clostridiales; f__Christensenellaceae; g__; s__	0.930918626	0.039495853
4452400	k__Bacteria; p__Firmicutes; c__Clostridia; o__Clostridiales; f__Lachnospiraceae; g__; s__	0.937209241	0.040291115
2498994	k__Bacteria; p__Firmicutes; c__Erysipelotrichi; o__Erysipelotrichales; f__Erysipelotrichaceae; g__; s__	0.898741423	0.040360774
4476950	k__Bacteria; p__Firmicutes; c__Clostridia; o__Clostridiales; f__[Tissierellaceae]; g__Anaerococcus; s__	0.933637927	0.040360774
312586	k__Bacteria; p__Firmicutes; c__Clostridia; o__Clostridiales; f__Lachnospiraceae; g__; s__	0.897927085	0.041397441

295258	k__Bacteria; p__Firmicutes; c__Clostridia; o__Clostridiales; f__Ruminococcaceae; g__; s__	0.899550502	0.041397441
178991	k__Bacteria; p__Firmicutes; c__Clostridia; o__Clostridiales; f__Lachnospiraceae; g__; s__	0.906695132	0.041397441
174300	k__Bacteria; p__Firmicutes; c__Clostridia; o__Clostridiales; f__Ruminococcaceae; g__; s__	0.8968239	0.041983748
181174	k__Bacteria; p__Firmicutes; c__Clostridia; o__Clostridiales; f__Ruminococcaceae; g__; s__	1.115994088	0.041983748
332929	k__Bacteria; p__Firmicutes; c__Clostridia; o__Clostridiales; f__Ruminococcaceae; g__; s__	0.887373173	0.042054871
4423553	k__Bacteria; p__Firmicutes; c__Clostridia; o__Clostridiales; f__Ruminococcaceae; g__; s__	0.901131947	0.042783657
2835813	k__Bacteria; p__Firmicutes; c__Clostridia; o__Clostridiales; f__Ruminococcaceae; g__; s__	0.899550521	0.042968746
178474	k__Bacteria; p__Firmicutes; c__Clostridia; o__Clostridiales; f__Lachnospiraceae; g__; s__	0.896570814	0.043115954
3903651	k__Bacteria; p__Firmicutes; c__Clostridia; o__Clostridiales; f__Ruminococcaceae; g__Oscillospira; s__	-0.860915649	0.043154468
2957436	k__Bacteria; p__Firmicutes; c__Clostridia; o__Clostridiales; f__Lachnospiraceae; g__Dorea; s__	0.995883865	0.043221361
1835985	k__Bacteria; p__Firmicutes; c__Clostridia; o__Clostridiales; f__; g__; s__	0.960205549	0.04337433
190301	k__Bacteria; p__Firmicutes; c__Clostridia; o__Clostridiales; f__Ruminococcaceae; g__; s__	-0.976376117	0.043413435
3424669	k__Bacteria; p__Firmicutes; c__Clostridia; o__Clostridiales; f__Lachnospiraceae; g__; s__	0.901515315	0.043457671
102471	k__Bacteria; p__Firmicutes; c__Clostridia; o__Clostridiales; f__; g__; s__	0.954854387	0.044062613
2251909	k__Bacteria; p__Firmicutes; c__Clostridia; o__Clostridiales; f__Peptostreptococcaceae; g__; s__	-0.905156895	0.045301932
4393565	k__Bacteria; p__Firmicutes; c__Clostridia; o__Clostridiales; f__; g__; s__	0.869716498	0.045580936
368203	k__Bacteria; p__Firmicutes; c__Clostridia; o__Clostridiales; f__; g__; s__	0.864182796	0.046501329
363430	k__Bacteria; p__Firmicutes; c__Clostridia; o__Clostridiales; f__; g__; s__	0.987158715	0.046501329
4380039	k__Bacteria; p__Firmicutes; c__Clostridia; o__Clostridiales; f__Lachnospiraceae; g__; s__	0.8634584	0.046544508
4331364	k__Bacteria; p__Firmicutes; c__Clostridia; o__Clostridiales; f__Lachnospiraceae; g__; s__	0.86754407	0.046544508
328472	k__Bacteria; p__Actinobacteria; c__Actinobacteria; o__Actinomycetales; f__Actinomycetaceae; g__Varibaculum; s__	0.894383735	0.046578924
189820	k__Bacteria; p__Firmicutes; c__Clostridia; o__Clostridiales; f__Ruminococcaceae; g__Ruminococcus; s__	0.897743183	0.046893606
195015	k__Bacteria; p__Firmicutes; c__Clostridia; o__Clostridiales; f__Lachnospiraceae; g__Lachnospira; s__	0.866251029	0.047021427
185584	k__Bacteria; p__Firmicutes; c__Clostridia; o__Clostridiales; f__Ruminococcaceae; g__; s__	-1.020305909	0.047826044
1868703	k__Bacteria; p__Firmicutes; c__Clostridia; o__Clostridiales; f__Lachnospiraceae; g__; s__	0.864416638	0.047959814
4434334	k__Bacteria; p__Firmicutes; c__Clostridia; o__Clostridiales; f__Clostridiaceae; g__; s__	1.022980315	0.048241937
179148	k__Bacteria; p__Firmicutes; c__Clostridia; o__Clostridiales; f__; g__; s__	0.964345899	0.048376777
4442130	k__Bacteria; p__Firmicutes; c__Bacilli; o__Lactobacillales; f__Streptococcaceae; g__Streptococcus; s__	0.847516616	0.048757183
4309760	k__Bacteria; p__Firmicutes; c__Clostridia; o__Clostridiales; f__Ruminococcaceae; g__; s__	0.960233145	0.048984162
177207	k__Bacteria; p__Firmicutes; c__Clostridia; o__Clostridiales; f__; g__; s__	-0.90250006	0.049983864
