# Supplementary material for: Multiple Sclerosis-Associated Changes in the Composition and Immune Functions of Spore-Forming Bacteria
Source: mSystems. 2018 Nov 6;3(6):e00083-18. doi: 10.1128/mSystems.00083-18 (PMC6222044; doi:10.1128/mSystems.00083-18)
Supplement: TABLE S4 [file sys006182286st4.pdf]

Supplementary Table 4. Differentially abundant genera in mice

Genus (blue: more in CTRL colonized, red: more in MS colonized)
k__Bacteria;p__Firmicutes;c__Bacilli;o__Lactobacillales;f__Enterococcaceae;g__Enterococcus
k__Bacteria;p__Firmicutes;c__Clostridia;o__Clostridiales;f__Clostridiaceae;g__SMB53
k__Bacteria;p__Firmicutes;c__Clostridia;o__Clostridiales;f__Dehalobacteriaceae;g__Dehalobacterium
k__Bacteria;p__Firmicutes;c__Clostridia;o__Clostridiales;f__Lachnospiraceae;g__
k__Bacteria;p__Firmicutes;c__Clostridia;o__Clostridiales;f__Lachnospiraceae;g__Coprococcus
k__Bacteria;p__Firmicutes;c__Clostridia;o__Clostridiales;f__Peptococcaceae;g__rc4-4
k__Bacteria;p__Firmicutes;c__Clostridia;o__Clostridiales;f__Ruminococcaceae;g__
k__Bacteria;p__Firmicutes;c__Clostridia;o__Clostridiales;f__Ruminococcaceae;g__Faecalibacterium
k__Bacteria;p__Firmicutes;c__Erysipelotrichi;o__Erysipelotrichales;f__Erysipelotrichaceae;g__[Eubacterium]
k__Bacteria;p__Firmicutes;c__Erysipelotrichi;o__Erysipelotrichales;f__Erysipelotrichaceae;g__Allobaculum
k__Bacteria;p__Proteobacteria;c__Gammaproteobacteria;o__Enterobacteriales;f__Enterobacteriaceae;g__
k__Bacteria;p__Proteobacteria;c__Gammaproteobacteria;o__Enterobacteriales;f__Enterobacteriaceae;g__Salmonella

log2fold(MS/CTRL)	adjusted p value
-1.45	3.58E-03
-1.56	7.58E-03
-1.36	5.64E-03
-1.06	3.36E-02
-1.37	1.29E-02
1.92	8.70E-04
1.25	1.11E-02
3.28	1.17E-08
2.37	9.08E-06
-2.16	8.70E-04
-1.50	3.30E-02
2.15	8.70E-04
