# Supplementary material for: Multiple Sclerosis-Associated Changes in the Composition and Immune Functions of Spore-Forming Bacteria
Source: mSystems. 2018 Nov 6;3(6):e00083-18. doi: 10.1128/mSystems.00083-18 (PMC6222044; doi:10.1128/mSystems.00083-18)
Supplement: TABLE S5 [file sys006182286st5.pdf]

Supplementary Table 5. Differentially abundant OTUs in mice

OTU (GreenGenes v.13.8)	taxonomy (blue: more in CTRL, red: more in MS)
461524	k__Bacteria; p__Actinobacteria; c__Coriobacteriia; o__Coriobacteriales; f__Coriobacteriaceae; g__; s__
199403	k__Bacteria; p__Actinobacteria; c__Coriobacteriia; o__Coriobacteriales; f__Coriobacteriaceae; g__Adlercreutzia; s__
465433	k__Bacteria; p__Bacteroidetes; c__Bacteroidia; o__Bacteroidales; f__Rikenellaceae; g__; s__
4449518	k__Bacteria; p__Bacteroidetes; c__Bacteroidia; o__Bacteroidales; f__Rikenellaceae; g__; s__
173852	k__Bacteria; p__Bacteroidetes; c__Bacteroidia; o__Bacteroidales; f__S24-7; g__; s__
276509	k__Bacteria; p__Bacteroidetes; c__Bacteroidia; o__Bacteroidales; f__S24-7; g__; s__
197038	k__Bacteria; p__Bacteroidetes; c__Bacteroidia; o__Bacteroidales; f__S24-7; g__; s__
194709	k__Bacteria; p__Bacteroidetes; c__Bacteroidia; o__Bacteroidales; f__S24-7; g__; s__
196733	k__Bacteria; p__Bacteroidetes; c__Bacteroidia; o__Bacteroidales; f__S24-7; g__; s__
134762	k__Bacteria; p__Bacteroidetes; c__Bacteroidia; o__Bacteroidales; f__S24-7; g__; s__
206324	k__Bacteria; p__Bacteroidetes; c__Bacteroidia; o__Bacteroidales; f__S24-7; g__; s__
176760	k__Bacteria; p__Bacteroidetes; c__Bacteroidia; o__Bacteroidales; f__S24-7; g__; s__
3231096	k__Bacteria; p__Bacteroidetes; c__Bacteroidia; o__Bacteroidales; f__S24-7; g__; s__
264734	k__Bacteria; p__Bacteroidetes; c__Bacteroidia; o__Bacteroidales; f__S24-7; g__; s__
174004	k__Bacteria; p__Bacteroidetes; c__Bacteroidia; o__Bacteroidales; f__S24-7; g__; s__
193038	k__Bacteria; p__Bacteroidetes; c__Bacteroidia; o__Bacteroidales; f__S24-7; g__; s__
181589	k__Bacteria; p__Firmicutes; c__Bacilli; o__Bacillales; f__Staphylococcaceae; g__Staphylococcus; s__equorum
4312974	k__Bacteria; p__Firmicutes; c__Bacilli; o__Bacillales; f__Staphylococcaceae; g__Staphylococcus; s__succinus
588755	k__Bacteria; p__Firmicutes; c__Bacilli; o__Lactobacillales; f__Enterococcaceae; g__Enterococcus; s__
197706	k__Bacteria; p__Firmicutes; c__Clostridia; o__Clostridiales; f__; g__; s__
177742	k__Bacteria; p__Firmicutes; c__Clostridia; o__Clostridiales; f__; g__; s__
259846	k__Bacteria; p__Firmicutes; c__Clostridia; o__Clostridiales; f__; g__; s__
268063	k__Bacteria; p__Firmicutes; c__Clostridia; o__Clostridiales; f__; g__; s__
323614	k__Bacteria; p__Firmicutes; c__Clostridia; o__Clostridiales; f__; g__; s__
264155	k__Bacteria; p__Firmicutes; c__Clostridia; o__Clostridiales; f__; g__; s__
356073	k__Bacteria; p__Firmicutes; c__Clostridia; o__Clostridiales; f__; g__; s__
628218	k__Bacteria; p__Firmicutes; c__Clostridia; o__Clostridiales; f__; g__; s__
331747	k__Bacteria; p__Firmicutes; c__Clostridia; o__Clostridiales; f__; g__; s__
259631	k__Bacteria; p__Firmicutes; c__Clostridia; o__Clostridiales; f__; g__; s__
342786	k__Bacteria; p__Firmicutes; c__Clostridia; o__Clostridiales; f__; g__; s__
259556	k__Bacteria; p__Firmicutes; c__Clostridia; o__Clostridiales; f__; g__; s__
318818	k__Bacteria; p__Firmicutes; c__Clostridia; o__Clostridiales; f__; g__; s__
258232	k__Bacteria; p__Firmicutes; c__Clostridia; o__Clostridiales; f__; g__; s__
319219	k__Bacteria; p__Firmicutes; c__Clostridia; o__Clostridiales; f__; g__; s__
450399	k__Bacteria; p__Firmicutes; c__Clostridia; o__Clostridiales; f__; g__; s__
250270	k__Bacteria; p__Firmicutes; c__Clostridia; o__Clostridiales; f__; g__; s__
839215	k__Bacteria; p__Firmicutes; c__Clostridia; o__Clostridiales; f__; g__; s__
337494	k__Bacteria; p__Firmicutes; c__Clostridia; o__Clostridiales; f__; g__; s__
174770	k__Bacteria; p__Firmicutes; c__Clostridia; o__Clostridiales; f__; g__; s__
173739	k__Bacteria; p__Firmicutes; c__Clostridia; o__Clostridiales; f__; g__; s__
130335	k__Bacteria; p__Firmicutes; c__Clostridia; o__Clostridiales; f__; g__; s__
228043	k__Bacteria; p__Firmicutes; c__Clostridia; o__Clostridiales; f__; g__; s__
175193	k__Bacteria; p__Firmicutes; c__Clostridia; o__Clostridiales; f__; g__; s__
177195	k__Bacteria; p__Firmicutes; c__Clostridia; o__Clostridiales; f__; g__; s__
322776	k__Bacteria; p__Firmicutes; c__Clostridia; o__Clostridiales; f__; g__; s__
337336	k__Bacteria; p__Firmicutes; c__Clostridia; o__Clostridiales; f__; g__; s__
163997	k__Bacteria; p__Firmicutes; c__Clostridia; o__Clostridiales; f__; g__; s__
192777	k__Bacteria; p__Firmicutes; c__Clostridia; o__Clostridiales; f__; g__; s__
174824	k__Bacteria; p__Firmicutes; c__Clostridia; o__Clostridiales; f__; g__; s__
175758	k__Bacteria; p__Firmicutes; c__Clostridia; o__Clostridiales; f__; g__; s__
332865	k__Bacteria; p__Firmicutes; c__Clostridia; o__Clostridiales; f__; g__; s__
320490	k__Bacteria; p__Firmicutes; c__Clostridia; o__Clostridiales; f__; g__; s__
189476	k__Bacteria; p__Firmicutes; c__Clostridia; o__Clostridiales; f__; g__; s__
314963	k__Bacteria; p__Firmicutes; c__Clostridia; o__Clostridiales; f__; g__; s__
321195	k__Bacteria; p__Firmicutes; c__Clostridia; o__Clostridiales; f__; g__; s__
1857626	k__Bacteria; p__Firmicutes; c__Clostridia; o__Clostridiales; f__; g__; s__
176957	k__Bacteria; p__Firmicutes; c__Clostridia; o__Clostridiales; f__; g__; s__
194662	k__Bacteria; p__Firmicutes; c__Clostridia; o__Clostridiales; f__; g__; s__
537548	k__Bacteria; p__Firmicutes; c__Clostridia; o__Clostridiales; f__; g__; s__
342504	k__Bacteria; p__Firmicutes; c__Clostridia; o__Clostridiales; f__Clostridiaceae; g__; s__
3576174	k__Bacteria; p__Firmicutes; c__Clostridia; o__Clostridiales; f__Clostridiaceae; g__; s__
319708	k__Bacteria; p__Firmicutes; c__Clostridia; o__Clostridiales; f__Clostridiaceae; g__SMB53; s__
314374	k__Bacteria; p__Firmicutes; c__Clostridia; o__Clostridiales; f__Dehalobacteriaceae; g__Dehalobacterium; s__
274136	k__Bacteria; p__Firmicutes; c__Clostridia; o__Clostridiales; f__Lachnospiraceae; g__; s__
296041	k__Bacteria; p__Firmicutes; c__Clostridia; o__Clostridiales; f__Lachnospiraceae; g__; s__
354800	k__Bacteria; p__Firmicutes; c__Clostridia; o__Clostridiales; f__Lachnospiraceae; g__; s__
303479	k__Bacteria; p__Firmicutes; c__Clostridia; o__Clostridiales; f__Lachnospiraceae; g__; s__
191878	k__Bacteria; p__Firmicutes; c__Clostridia; o__Clostridiales; f__Lachnospiraceae; g__; s__
790663	k__Bacteria; p__Firmicutes; c__Clostridia; o__Clostridiales; f__Lachnospiraceae; g__; s__
214835	k__Bacteria; p__Firmicutes; c__Clostridia; o__Clostridiales; f__Lachnospiraceae; g__; s__
334619	k__Bacteria; p__Firmicutes; c__Clostridia; o__Clostridiales; f__Lachnospiraceae; g__; s__
259912	k__Bacteria; p__Firmicutes; c__Clostridia; o__Clostridiales; f__Lachnospiraceae; g__; s__
262104	k__Bacteria; p__Firmicutes; c__Clostridia; o__Clostridiales; f__Lachnospiraceae; g__; s__

258169	k__Bacteria; p__Firmicutes; c__Clostridia; o__Clostridiales; f__Lachnospiraceae; g__; s__
658370	k__Bacteria; p__Firmicutes; c__Clostridia; o__Clostridiales; f__Lachnospiraceae; g__; s__
305177	k__Bacteria; p__Firmicutes; c__Clostridia; o__Clostridiales; f__Lachnospiraceae; g__; s__
312062	k__Bacteria; p__Firmicutes; c__Clostridia; o__Clostridiales; f__Lachnospiraceae; g__; s__
352022	k__Bacteria; p__Firmicutes; c__Clostridia; o__Clostridiales; f__Lachnospiraceae; g__; s__
260666	k__Bacteria; p__Firmicutes; c__Clostridia; o__Clostridiales; f__Lachnospiraceae; g__; s__
334751	k__Bacteria; p__Firmicutes; c__Clostridia; o__Clostridiales; f__Lachnospiraceae; g__; s__
268620	k__Bacteria; p__Firmicutes; c__Clostridia; o__Clostridiales; f__Lachnospiraceae; g__; s__
173892	k__Bacteria; p__Firmicutes; c__Clostridia; o__Clostridiales; f__Lachnospiraceae; g__; s__
759751	k__Bacteria; p__Firmicutes; c__Clostridia; o__Clostridiales; f__Lachnospiraceae; g__; s__
4045882	k__Bacteria; p__Firmicutes; c__Clostridia; o__Clostridiales; f__Lachnospiraceae; g__; s__
317740	k__Bacteria; p__Firmicutes; c__Clostridia; o__Clostridiales; f__Lachnospiraceae; g__; s__
362539	k__Bacteria; p__Firmicutes; c__Clostridia; o__Clostridiales; f__Lachnospiraceae; g__; s__
366797	k__Bacteria; p__Firmicutes; c__Clostridia; o__Clostridiales; f__Lachnospiraceae; g__; s__
533969	k__Bacteria; p__Firmicutes; c__Clostridia; o__Clostridiales; f__Lachnospiraceae; g__; s__
336872	k__Bacteria; p__Firmicutes; c__Clostridia; o__Clostridiales; f__Lachnospiraceae; g__[Ruminococcus]; s__gnavus
273776	k__Bacteria; p__Firmicutes; c__Clostridia; o__Clostridiales; f__Lachnospiraceae; g__[Ruminococcus]; s__gnavus
330116	k__Bacteria; p__Firmicutes; c__Clostridia; o__Clostridiales; f__Lachnospiraceae; g__[Ruminococcus]; s__gnavus
279107	k__Bacteria; p__Firmicutes; c__Clostridia; o__Clostridiales; f__Lachnospiraceae; g__Anaerostipes; s__
195189	k__Bacteria; p__Firmicutes; c__Clostridia; o__Clostridiales; f__Lachnospiraceae; g__Coprococcus; s__
192878	k__Bacteria; p__Firmicutes; c__Clostridia; o__Clostridiales; f__Lachnospiraceae; g__Coprococcus; s__
273657	k__Bacteria; p__Firmicutes; c__Clostridia; o__Clostridiales; f__Peptococcaceae; g__rc4-4; s__
263895	k__Bacteria; p__Firmicutes; c__Clostridia; o__Clostridiales; f__Peptococcaceae; g__rc4-4; s__
97263	k__Bacteria; p__Firmicutes; c__Clostridia; o__Clostridiales; f__Ruminococcaceae; g__; s__
263252	k__Bacteria; p__Firmicutes; c__Clostridia; o__Clostridiales; f__Ruminococcaceae; g__; s__
275061	k__Bacteria; p__Firmicutes; c__Clostridia; o__Clostridiales; f__Ruminococcaceae; g__; s__
209845	k__Bacteria; p__Firmicutes; c__Clostridia; o__Clostridiales; f__Ruminococcaceae; g__; s__
353982	k__Bacteria; p__Firmicutes; c__Clostridia; o__Clostridiales; f__Ruminococcaceae; g__; s__
185923	k__Bacteria; p__Firmicutes; c__Clostridia; o__Clostridiales; f__Ruminococcaceae; g__; s__
261132	k__Bacteria; p__Firmicutes; c__Clostridia; o__Clostridiales; f__Ruminococcaceae; g__; s__
1111580	k__Bacteria; p__Firmicutes; c__Clostridia; o__Clostridiales; f__Ruminococcaceae; g__; s__
462727	k__Bacteria; p__Firmicutes; c__Clostridia; o__Clostridiales; f__Ruminococcaceae; g__; s__
309720	k__Bacteria; p__Firmicutes; c__Clostridia; o__Clostridiales; f__Ruminococcaceae; g__; s__
1111782	k__Bacteria; p__Firmicutes; c__Clostridia; o__Clostridiales; f__Ruminococcaceae; g__; s__
273648	k__Bacteria; p__Firmicutes; c__Clostridia; o__Clostridiales; f__Ruminococcaceae; g__; s__
110060	k__Bacteria; p__Firmicutes; c__Clostridia; o__Clostridiales; f__Ruminococcaceae; g__; s__
188348	k__Bacteria; p__Firmicutes; c__Clostridia; o__Clostridiales; f__Ruminococcaceae; g__; s__
4430499	k__Bacteria; p__Firmicutes; c__Clostridia; o__Clostridiales; f__Ruminococcaceae; g__; s__
188225	k__Bacteria; p__Firmicutes; c__Clostridia; o__Clostridiales; f__Ruminococcaceae; g__; s__
230421	k__Bacteria; p__Firmicutes; c__Clostridia; o__Clostridiales; f__Ruminococcaceae; g__; s__
334267	k__Bacteria; p__Firmicutes; c__Clostridia; o__Clostridiales; f__Ruminococcaceae; g__; s__
317135	k__Bacteria; p__Firmicutes; c__Clostridia; o__Clostridiales; f__Ruminococcaceae; g__; s__
179201	k__Bacteria; p__Firmicutes; c__Clostridia; o__Clostridiales; f__Ruminococcaceae; g__; s__
311961	k__Bacteria; p__Firmicutes; c__Clostridia; o__Clostridiales; f__Ruminococcaceae; g__Anaerotruncus; s__
339087	k__Bacteria; p__Firmicutes; c__Clostridia; o__Clostridiales; f__Ruminococcaceae; g__Faecalibacterium; s__prausnitzii
198194	k__Bacteria; p__Firmicutes; c__Clostridia; o__Clostridiales; f__Ruminococcaceae; g__Oscillospira; s__
187807	k__Bacteria; p__Firmicutes; c__Clostridia; o__Clostridiales; f__Ruminococcaceae; g__Oscillospira; s__
273724	k__Bacteria; p__Firmicutes; c__Clostridia; o__Clostridiales; f__Ruminococcaceae; g__Oscillospira; s__
274083	k__Bacteria; p__Firmicutes; c__Clostridia; o__Clostridiales; f__Ruminococcaceae; g__Oscillospira; s__
313274	k__Bacteria; p__Firmicutes; c__Clostridia; o__Clostridiales; f__Ruminococcaceae; g__Oscillospira; s__
581933	k__Bacteria; p__Firmicutes; c__Clostridia; o__Clostridiales; f__Ruminococcaceae; g__Oscillospira; s__
367176	k__Bacteria; p__Firmicutes; c__Clostridia; o__Clostridiales; f__Ruminococcaceae; g__Oscillospira; s__
277588	k__Bacteria; p__Firmicutes; c__Clostridia; o__Clostridiales; f__Ruminococcaceae; g__Oscillospira; s__
312455	k__Bacteria; p__Firmicutes; c__Clostridia; o__Clostridiales; f__Ruminococcaceae; g__Oscillospira; s__
182033	k__Bacteria; p__Firmicutes; c__Clostridia; o__Clostridiales; f__Ruminococcaceae; g__Oscillospira; s__
297111	k__Bacteria; p__Firmicutes; c__Clostridia; o__Clostridiales; f__Ruminococcaceae; g__Oscillospira; s__
274297	k__Bacteria; p__Firmicutes; c__Clostridia; o__Clostridiales; f__Ruminococcaceae; g__Oscillospira; s__
335550	k__Bacteria; p__Firmicutes; c__Clostridia; o__Clostridiales; f__Ruminococcaceae; g__Oscillospira; s__
1504042	k__Bacteria; p__Firmicutes; c__Clostridia; o__Clostridiales; f__Ruminococcaceae; g__Oscillospira; s__
304777	k__Bacteria; p__Firmicutes; c__Clostridia; o__Clostridiales; f__Ruminococcaceae; g__Oscillospira; s__
359120	k__Bacteria; p__Firmicutes; c__Clostridia; o__Clostridiales; f__Ruminococcaceae; g__Oscillospira; s__
178480	k__Bacteria; p__Firmicutes; c__Clostridia; o__Clostridiales; f__Ruminococcaceae; g__Ruminococcus; s__
238487	k__Bacteria; p__Firmicutes; c__Clostridia; o__Clostridiales; f__Ruminococcaceae; g__Ruminococcus; s__
591635	k__Bacteria; p__Firmicutes; c__Clostridia; o__Clostridiales; f__Ruminococcaceae; g__Ruminococcus; s__
145801	k__Bacteria; p__Firmicutes; c__Erysipelotrichi; o__Erysipelotrichales; f__Erysipelotrichaceae; g__; s__
830080	k__Bacteria; p__Firmicutes; c__Erysipelotrichi; o__Erysipelotrichales; f__Erysipelotrichaceae; g__; s__
548587	k__Bacteria; p__Firmicutes; c__Erysipelotrichi; o__Erysipelotrichales; f__Erysipelotrichaceae; g__[Eubacterium]; s__dolichum
274912	k__Bacteria; p__Firmicutes; c__Erysipelotrichi; o__Erysipelotrichales; f__Erysipelotrichaceae; g__Allobaculum; s__
258421	k__Bacteria; p__Firmicutes; c__Erysipelotrichi; o__Erysipelotrichales; f__Erysipelotrichaceae; g__Allobaculum; s__
4425571	k__Bacteria; p__Proteobacteria; c__Gammaproteobacteria; o__Enterobacteriales; f__Enterobacteriaceae; g__; s__
4384058	k__Bacteria; p__Proteobacteria; c__Gammaproteobacteria; o__Enterobacteriales; f__Enterobacteriaceae; g__Salmonella; s__
178331	k__Bacteria; p__Verrucomicrobia; c__Verrucomicrobiae; o__Verrucomicrobiales; f__Verrucomicrobiaceae; g__Akkermansia; s__muciniphila
359376	k__Bacteria; p__Verrucomicrobia; c__Verrucomicrobiae; o__Verrucomicrobiales; f__Verrucomicrobiaceae; g__Akkermansia; s__muciniphila
193829	k__Bacteria; p__Verrucomicrobia; c__Verrucomicrobiae; o__Verrucomicrobiales; f__Verrucomicrobiaceae; g__Akkermansia; s__muciniphila
